# Initial physical health assessment for psychosis in Australia and New Zealand: 2026 recommendations

**DOI:** 10.1177/00048674261435740

**Published:** 2026-04-26

**Authors:** Nicola Warren, Cullen O’Gorman, Frances Dark, Susanna Every-Palmer, Sean Halstead, Nicole Korman, Julia Lappin, Sharon Lawn, Brian O’Donoghue, Shuichi Suetani, Andrew Thompson, Samantha M. Loi, James G. Scott, Naomi Runnegar, Toby Pillinger, Robert A. McCutcheon, Graham Blackman, Thomas Skerlj, Jessemin Firman, Dan Siskind

**Affiliations:** 1 Medical School, The University of Queensland, Brisbane, QLD, Australia; 2 Queensland Centre for Mental Health Research, West Moreton, QLD, Australia; 3 Metro South Addiction and Mental Health Services, Brisbane, QLD, Australia; 4 Department of Neurology, Princess Alexandra Hospital, Woolloongabba, QLD, Australia; 5 Department of Psychological Medicine, University of Otago, Wellington, New Zealand; 6 Te Whatu Ora Health New Zealand, Wellington, New Zealand; 7 Discipline of Psychiatry and Mental Health, University of New South Wales, Sydney, NSW, Australia; 8 College of Medicine and Public Health, Flinders University, Adelaide, SA, Australia; 9 Lived Experience Australia, Adelaide, SA, Australia; 10 Department of Psychiatry, University College Dublin, Dublin, Ireland; 11 St Vincent’s University Hospital, Dublin, Ireland; 12 Institute for Urban Indigenous Health, Windsor, QLD, Australia; 13 Orygen, Parkville, VIC, Australia; 14 Centre for Youth Mental Health, University of Melbourne, Parkville, VIC, Australia; 15 Department of Psychiatry, University of Melbourne, Parkville, VIC, Australia; 16 Neuropsychiatry Centre, Royal Melbourne Hospital, Parkville, VIC, Australia; 17 Child and Youth Mental Health Services, Children’s Health Queensland, South Brisbane, QLD, Australia; 18 Child Health Research Centre, The University of Queensland, South Brisbane, QLD, Australia; 19 Department of Infectious Disease, Princess Alexandra Hospital, Woolloongabba, QLD, Australia; 20 South London and Maudsley NHS Foundation Trust, London, UK; 21 Department of Psychosis Studies, Institute of Psychiatry, Psychology and Neuroscience, King’s College, London, UK; 22 Department of Psychiatry, University of Oxford, Oxford, UK; 23 Oxford Health NHS Foundation Trust, Oxford, UK

**Keywords:** Psychosis, schizophrenia, organic, medical, guideline

## Abstract

**Objective:**

Initial presentations of psychosis require a thorough physical health assessment to identify comorbidities, establish treatment safety and exclude organic causes of psychosis. Despite clinical consensus that these assessments are essential, global guidelines are variable and outdated. This work aimed to synthesise current evidence to inform updated recommendations for physical assessments in psychosis, balancing thorough investigation with practical applicability.

**Methods:**

A scoping review of physical health disorders associated with psychosis was conducted using PubMed, Embase and CINAHL. Separately, a systematic review of international guidelines from 2000 to 2025 was performed, extracting physical health assessment recommendations for schizophrenia spectrum disorders. A narrative analysis evaluated the clinical utility of identified investigations.

**Results:**

Eighty-four physical health disorders with potential psychotic presentations were identified, mostly rare and typically associated with other neurological or systemic features. There was significant heterogeneity in investigations advised by the 25 identified guidelines, outside of the common consideration for metabolic screening. The majority of guidelines considered investigations for both the exclusion of organic causes of psychosis and identifying a physical health baseline or comorbidity. There was limited consistency around recommendations for neuroimaging or autoimmune screening. Clinical assessment remains central to determining appropriate investigations.

**Conclusion:**

Global inconsistency in assessment recommendations reflects the complexity of distinguishing organic psychoses from primary psychiatric disorders. Structured yet individualised assessments, informed by symptomatology and risk factors, are essential. A staged, context-sensitive approach is proposed to optimise diagnostic accuracy and avoid unnecessary testing. Updated, evidence-informed guidelines are critical for improving care for people with psychosis.

## Introduction

When an individual first presents with psychosis to a health service, it is an expectation that a thorough physical assessment is completed. This serves multiple purposes: to exclude organic causes of psychotic symptoms, to assess for physical health comorbidity, to document baseline measures prior to psychotropic initiation and to ensure these medications can be safely commenced. While individually tailored assessment and care are required, this should be informed by guideline-driven minimum recommendations, particularly as cognitive bias from health care providers and diagnostic overshadowing can lead to underappreciation of physical health disorders in individuals with psychiatric presentations (Halstead et al., 2024b). This is likely magnified in busy clinical services (Conigliaro et al., 2018). Equally, broad and undifferentiated screening for rare medical conditions is unlikely to be informative and may cause unnecessary alarm and delay in psychiatric care (Conigliaro et al., 2018; Gregory et al., 2004; McClellan and Stock, 2013; Sunshine and McClellan, 2023). As such, making recommendations for physical health assessment guidelines needs to balance individual needs and reflexive overtesting. An additional challenge is that the majority of psychiatric care is now provided in the community, meaning that outpatient clinics need to have the facilities or pathways to undertake these physical assessments and investigations.

Organic psychoses – psychotic illnesses caused by a physical health disorder – are thought to occur in around 5% of those presenting for the first time to health services (Blackman et al., 2024). Geographic location and age of population may influence the underlying pathology, with the most frequent cause of organic psychoses related to infections resulting in encephalomeningitis or delirium (Blackman et al., 2024). Other causes that need to be considered include seizure, autoimmune, metabolic, vascular, neurodevelopmental and genetic disorders, as well as central nervous system lesions and morphological changes (McClellan and Stock, 2013; Sunshine and McClellan, 2023; Blackman et al., 2024). Many of these disorders are themselves rare and uncommonly present with psychosis but are important to identify, as they may require alternative treatments or influence prognosis, illness course and psychiatric care. This may have significant impact on the person’s life, as well as flow-on effects to those who support them, including family, carers and kin.

Beyond exploring organic causes of psychosis, an initial physical health assessment should also identify conditions that require early medical intervention or impact psychiatric treatment. Individuals with primary psychotic disorders are known to have high levels of physical health comorbidity, compared to the general population, with elevated rates of physical illness seen from first presentation (Halstead et al., 2024a; Samele et al., 2007). Given the known physical adverse drug reactions (ADRs) associated with psychotropics (e.g. metabolic dysregulation), results of baseline measures may dictate early intervention and the frequency of review (Labad et al., 2020; Pillinger et al., 2020). In addition, choice, dose and titration of psychotropics may also be influenced by cardiac history, electrocardiogram (ECG) measures, as well as renal and liver function.

The Australian and New Zealand 2016 Schizophrenia guidelines recommendations for initial physical health assessment were based on Freudenreich et al.’s (2009) expert opinion and review of earlier guidelines (Freudenreich et al., 2009; Galletly et al., 2016). Due to substantial growth in our understanding of emerging topics, such as autoimmune encephalitis, there have been multiple recent updates to international guidelines. The aim of this work was to review (1) current understanding of organic psychoses, their epidemiology, presentations and how to differentiate from primary psychotic disorders; (2) global recommendations for assessment and investigations at initial presentation of psychosis and (3) utility of the investigations identified in both the review of organic psychoses and guidelines, in order to produce up-to-date and evidence-based recommendations for initial physical health assessment in individuals who present with psychosis to primary or secondary healthcare. This includes recommendations that may be specific to particular populations, such as children or older persons.

## Methods

The Preferred Reporting Items for Systematic Review and Meta-Analyses (PRISMA) and extension for scoping reviews (PRISMA-ScR) were followed for assessing organic psychosis and global guidelines, with a narrative approach to exploring the identified investigations (Page et al., 2021; Tricco et al., 2018). Complete methodology is contained in Supplemental Appendix 1 and summarised here.

**Figure 1.**
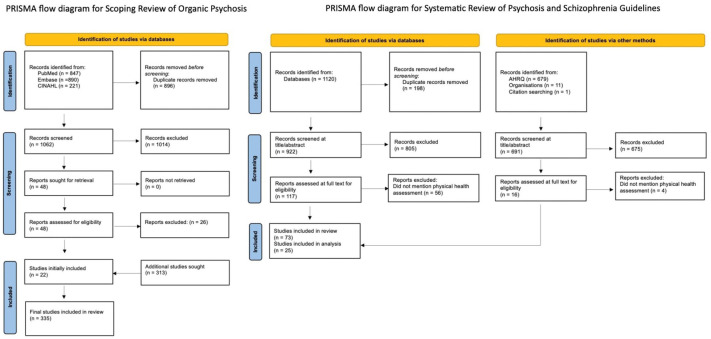
PRISMA flow diagrams.

First, a scoping review on organic psychoses was conducted using PubMed, Embase and CINAHL to identify reviews of physical health causes of psychosis (Figure 1). A list of causes was compiled with prevalence/incidence of the disorder, prevalence/incidence of psychosis within the disorder, differentiating features of psychosis or other symptoms/signs and relevant investigations (Supplemental Appendix 2). Where this information was missing from the initial scoping, a further literature review was conducted. Hierarchy of evidence and recency of publication were prioritised and noted (Supplemental Appendix 3). Rarity of a disorder was based on Orphanet categorisation (Orphanet, 2023). For this review, causes of nonorganic psychoses, such as other psychiatric illnesses or substances, were not included.

Second, a systematic process to identify current physical health assessment guidelines or recommendations in psychosis or schizophrenia was undertaken through a search of PubMed, Embase and CINAHL, time-limited to studies published from January 2000 to February 2025 (Figure 1). The grey literature was searched using the Agency for Healthcare Research and Quality database and handsearching of psychiatric association webpages. Where the same group or country had produced multiple guidelines, all those within the time frame were initially included to allow comparison over time (Supplemental Appendix 4). For all eligible studies, the recommendations for physical health screening and specific processes (need for fasting, timing of investigation, routine vs clinically indicated, modality preference) were extracted (Supplemental Appendix 5). Of note, quality assessment of the guidelines was felt not necessarily to represent the quality of the section on physical health assessment.

The investigations identified in the scoping and systematic reviews were collated, and a narrative analysis of their utility in initial physical health assessment for individuals presenting with psychosis was conducted. Review of all results informed consensus opinion on recommendations. The Reporting Items for Practice Guidelines in HealThcare (RIGHT) Checklist was followed (Chen et al, 2017).

## Results

### Scoping review of organic psychoses

There were 84 physical health disorders identified that may present with psychotic symptoms at some point in their course of illness (Table 1, Figure 1). This includes broad clusters of multiple pathologies, such as stroke, delirium and traumatic brain injury, as well as a list of physical health medications that may cause psychosis (Supplemental Appendix 2). The majority were rare disorders (prevalence < 5/10,000 persons) where psychosis had only been described in case reports. Most frequently, psychotic symptoms occurred in the context of neurological, cognitive or constitutional disturbance or when there was a known medical history, overtly identifying an organic cause. Where isolated psychosis presentations or initial misdiagnosis of a primary psychotic disorder had occurred, these were noted (Supplemental Appendix 2).

**Table 1.** Organic psychoses.

	Not rare/rare	Rate of psychosis		Not rare/rare	Rate of psychosis
Epilepsy		6%	Klinefelter syndrome		12%
Multiple sclerosis		4.3%	Turner syndrome		2–3%
Neuromyelitis optica		case reports	Retinitis pigmentosa/usher syndrome		case reports/23%
ADEM		case reports	22q11.2 deletion (velocardiofacial syndrome)		30–40%
Autoimmune encephalitis		40–60% (NMDA)	Prader–Willi syndrome		9–60%
Systemic lupus erythematosus		1.5–11.3%	Williams–Beuren syndrome		case reports
Sjogren’s syndrome		case reports	Corpus callosum agenesis		32%
Antiphospholipid syndrome		case reports	Friedreich ataxia		18%
Neurosarcoidosis		case reports	Fahr’s disease		40%
Acute infectious encephalitis		case reports	Tuberous sclerosis		case reports
Prion (CJD) disease		36.9%	Amyloidosis		case reports
Neuroborreliosis		case reports	Mitochondrial disorders		case reports
Syphilis		case reports	Megalencephalic leukoencephalopathy		case reports
HIV		0.2–15.2%	Ornithine transcarbamylase deficiency		case reports
Subacute sclerosing panencephalitis		case reports	Maple syrup urine disease		case reports
Normal pressure hydrocephalus		3.1%	Neuroferritinopathy		case reports
Acute stroke		6.7%	Propionic acidemia		case reports
Chronic subdural haematoma		case reports	Cobalamin C disease		case reports
Vasculitis		case reports	Tay-Sachs disease		30–50%
AV malformation		case reports	Metachromatic leukodystrophy		50%
Migraine		case reports	Niemann-Pick disease type C		case reports
Cerebral neoplasm		case reports	Gaucher disease		case reports
Traumatic brain injury		0.7–20%	α-Mannosidosis		case reports
Malformation of cortical development		case reports	Neuronal ceroid lipofuscinoses		case reports
Porencephaly		case reports	X-adrenoleukodystrophy late onset		case reports
Delirium		9–70%	Cerebrotendinous Xanthomatosis		case reports
Huntington’s disease		5–18%	Porphyrias		case reports
Alzheimer’s disease		41%	Wilson’s disease		1.4–11.3%
Parkinson’s disease		60%	Phenylketonuria		case reports
Lewy body disease		80%	B12/cobalamin deficiency		case reports
Vascular dementia		37–46%	Pellagra/niacin/B3 deficiency		case reports
ALS/frontotemporal dementia		10–55%	Wernicke-Korsakoff/thiamine/B1 deficiency		10%
Hyperthyroidism		case reports	Cyanide toxicity		case reports
Hypothyroidism		15%	Manganese toxicity		case reports
EAAT (autoimmune thyroid encephalopathy)		20%	Arsenic toxicity		case reports
Hypoparathyroidism		case reports	Mercury toxicity		case reports
Hyperparathyroidism		case reports	Toluene and other solvents toxicity		case reports
Cushing’s syndrome		8%	Lead toxicity		case reports
Adrenal insufficiency		case reports	Narcolepsy		80%
Phaeochromocytoma		case reports	Kleine–Levin syndrome		case reports
Down syndrome		13–43%	High altitude cerebral oedema		case reports
Fragile X syndrome		case reports			

See Appendix for further details and references. ADEM: acute disseminated encephalomyelitis; CJD: Creutzfeldt-Jakob disease; HIV: human immunodeficiency virus; AV: arteriovenous; ALS: amyotrophic lateral sclerosis; EAAT: encephalopathy associated with autoimmune thyroid disease.

### Systematic review of global schizophrenia guidelines

There were 73 identified guidelines published from 2000 to 2023 that made recommendations for schizophrenia spectrum disorders (Supplemental Appendix 4, Figure 1). After selection of the most recent guidelines, which made recommendations for physical health assessment, 25 remained. Guidelines were developed by international groups (Hasan et al., 2017; Kane et al., 2003; Keshavan et al., 2006; Skikic and Arriola, 2020; Stahl et al., 2013) and national organisations: Australia/New Zealand (2016) (Galletly et al., 2016), Canada (Addington et al., 2017), Chile (Gaspar et al., 2019), Finland (2020), France (Llorca et al., 2013), (DGPPN e.V., 2019), Greece (Hadjulis et al., 2018), India (Grover and Avasthi, 2019), Malaysia (Ministry of Health, 2021), Poland (Wichniak et al., 2019), South Africa (Emsley et al., 2013), Spain (Bobes-García et al., 2012), the United Kingdom (Barnes et al., 2020; Nice, 2014; SIGN, 2013) and the United States (Association, 2020; Jonokuchi et al., 2023; Marder et al., 2004; McClellan and Stock, 2013) (Supplemental Appendix 5). Most guidelines did not focus on physical health assessment. Recommendations were developed through the combination of a nonsystematic literature review, review of existing guidelines and expert opinion.

Baseline and routine measurement of body mass index, weight and vital signs was recommended by the majority (80%). Specific recommendation for neurological and/or cardiovascular examination was infrequently documented (16%). Investigations for baseline assessment and organic screening, conducted as routine or when clinically indicated, are noted in Table 2. There were five guidelines (20%) noting that neuroimaging should be by magnetic resonance imaging (MRI) modality and that this should be done routinely. No guideline recommended routine functional neuroimaging. Neurocognitive assessment was recommended in seven guidelines (8% routine, 20% as indicated).

**Table 2.** Guideline-recommended investigations.

Investigation	Guidelines recommended (routine: indicated, total 25)
**Baseline assessment**	
Fasting glucose/HbA1c	100% (24:1)
Lipids	96% (24:0)
Renal function	76% (19:0)
Liver function	76% (19:0)
Full blood count	72% (18:0)
Prolactin	60% (9:7)
Thyroid function	56% (13:1)
HIV	40% (5:5)
Syphilis	36% (4:5)
Pregnancy assessment	32% (5:3)
Nutritional/vitamin status	28% (3:2)
Hepatitis B/C	24% (2:4)
ECG	88% (12:10)
**Organic screening**	
CRP/ESR	24% (6:0)
Antineuronal antibodies	24% (1:5)
Copper	24% (2:4)
Heavy metals	20% (0:5)
Karyotyping	20% (0:5)
ANA	16% (1:3)
EEG	64% (3:13)
Neuroimaging	64% (7:9)
UDS	52% (10:3)
CSF	28% (0:7)

HIV: human immunodeficiency virus; ECG: electrocardiogram; CRP: C-reactive protein; ESR: erythrocyte sedimentation rate; ANA: antinuclear antibodies; EEG: electroencephalogram; UDS: urine drug screen; CSF: cerebrospinal fluid.

Where there had been updates to guidelines during the studied time frame, more recent guidelines had increased overall recommendations for physical health assessment (Barnes et al., 2020; Early Psychosis Guidelines Writing Group and EPPIC National Support Program 2016; Galletly et al., 2016; Hasan et al., 2017; McClellan and Stock, 2013; Association, 2020) and specifically added recommendations for ECG (Barnes et al., 2020; Hasan et al., 2017) and antineuronal antibodies (Barnes et al., 2020; Galletly et al., 2016), as well as metabolic (McClellan and Stock, 2013), pregnancy (Hasan et al., 2017) and prolactin (Barnes et al., 2020; Early Psychosis Guidelines Writing Group and EPPIC National Support Program 2016) testing. In addition, there was a more recent recommendation to consider cerebrospinal fluid (CSF) analysis when clinically indicated (Barnes et al., 2020; Early Psychosis Guidelines Writing Group and EPPIC National Support Program 2016; Hasan et al., 2017; McClellan and Stock, 2013).

### Overview of investigations

#### Blood tests

Although clinically significant laboratory results are uncommon in the majority of people presenting to psychiatric services, it is reasonable to consider initial broad screening investigations of electrolytes, renal, liver and haematological functioning (Conigliaro et al., 2018; Eskelinen et al., 2020; Gregory et al., 2004). This will guide further targeted assessment and is also required for safe pharmacotherapy decision-making (Cohen et al., 2004; Rüttimann et al., 1992; Telles-Correia et al., 2017).

Baseline and continued monitoring of metabolic parameters is also important (Cohn and Sernyak, 2006); however, these do not need to be collected in the fasting state for the majority of individuals. The maximal mean change in lipid results 1–6 hours after meals is not clinically significant, and nonfasted lipids are comparable to fasted in the prediction of cardiovascular disease (Nordestgaard et al., 2016). Compared to fasting glucose, glycated haemoglobin is similarly associated with the risk of diabetes and more strongly associated with the risk of cardiovascular disease (Selvin et al., 2010). Glycated haemoglobin is equally appropriate for diabetes and prediabetes diagnoses, as compared with fasting glucose or the 2-hour glucose tolerance test, except in the case of concurrent haematological conditions, pregnancy or human immunodeficiency virus (HIV) treatment (ElSayed et al., 2022).

Thyroid function tests are frequently indiscriminately ordered. Although psychotic symptoms have been shown in up to 15% of individuals with hypothyroidism, and less commonly in hyperthyroidism, the physical symptoms associated with thyroid dysfunction, as well as mood and/or anxiety disturbance typically occur in the weeks to months prior to the onset of psychosis (Brownlie et al., 2000; Feldman et al., 2013; Hall, 1983; Heinrich and Grahm, 2003). Retrospective studies of both inpatient psychiatric admissions and community-based populations demonstrate very low rates of clinically relevant abnormal thyroid results, many of which were already identified on physical assessment (Arce-Cordon et al., 2007; Eskelinen et al., 2020; Garnier et al., 2016; Lachman et al., 2012). Testing thyroid function is therefore only recommended when history and examination indicate clinical concern (prominent mood/anxiety, goitre, unexplained change in weight, heat sensitivity, sleep, menstrual cycle, hair/skin or heart rate). It should, however, be noted that there is emerging evidence showing an association with subclinical decreases in thyroid-stimulating hormone (TSH) and first episode psychosis, as well as subclinical TSH increase with chronic presentations (Dickerman and Barnhill, 2012; Lachman et al., 2012; Misiak et al., 2021; Toll et al., 2023). Recent studies have also demonstrated an association between subclinical elevations in thyroxine with certain psychosis phenotypes and cognitive performance (Chen et al., 2025; Gine-Serven et al., 2023; Saglam et al., 2024). Although universal thyroid function testing is not supported currently in routine care, this recommendation may change in the future if thyroid hormone assessment can be shown to be beneficial as part of a multiomic prognosis and personalised treatment decision tool (Guan et al., 2022).

Hyperprolactinaemia most commonly occurs as a consequence of antipsychotic use or a pituitary prolactinoma, with other less common causes including hypothyroidism, chest wall trauma, seizures and renal or liver failure (Cookson et al., 2012). In addition, adipose tissue is an extrapituitary source of prolactin, and elevated levels have been seen in those with obesity, insulin resistance and type 2 diabetes (Corona et al., 2022; Papazoglou et al., 2024). People have varying sensitivity to prolactin, but mild elevations (900–1500 mU/L) typically result in decreased libido, infertility and changes to the menstrual cycle, with amenorrhea, galactorrhea, gynaecomastia and hypogonadism seen with marked prolactin excess (>3000 mU/L) (Cookson et al., 2012). Long-term consequences are significant and include sexual dysfunction, decreased bone mineral density, increased risk of cardiovascular and metabolic disease, and subsequent nonadherence to medication (Cookson et al., 2012; Corona et al., 2022; Papazoglou et al., 2024). More recently, the association between prolactin-raising antipsychotics and risk of breast cancer in women has also been demonstrated (Solmi et al., 2024). Prolactin has also been shown to act as a pro-inflammatory cytokine (Borba et al., 2018). There is some evidence that prolactin may be mildly elevated in some antipsychotic-naive persons with first episode psychosis (Aymerich et al., 2023) and will later occur in around 40% to 50% of those on antipsychotics, varying on the type of antipsychotic, dose, duration of treatment, sex and age (Bushe and Shaw, 2007; Montgomery et al., 2004). Given the frequency and consequences of hyperprolactinaemia, expert consensus and more recent guidelines recommend prolactin testing at baseline, 3 months after initiation or changes to dose/drug and then annually or when indicated by abnormal menstrual, sexual or neurological assessment (Peveler et al., 2008).

Nutritional deficiencies as a cause of psychosis in Australia and New Zealand is uncommon, but broad nutritional deficits are seen in those with schizophrenia (Cui et al., 2021; Firth et al., 2017; Yanik et al., 2004) and deficiencies of folate, vitamin D and C have been demonstrated in individuals with first episode psychosis (Firth et al., 2018). In saying this, the need for specific nutritional assessment should be indicated from a broader dietary and lifestyle assessment, with guidance from examination and full blood count (Lambert et al., 2017; Rüttimann et al., 1992). In addition, it should be appreciated that for some nutrients, there are no consistent laboratory definitions of deficiency and that there are individual and temporal variabilities in requirements (Binkley and Carter, 2017).

C-reactive protein (CRP) and erythrocyte sedimentation rate (ESR) are nonspecific, insensitive markers of acute and chronic inflammation that can also be altered by numerous physiological and noninflammatory factors (Osei-Bimpong et al., 2007). They may be elevated in causes of psychosis such as delirium, infective or autoimmune encephalitis, systemic lupus erythematosus (SLE), vasculitis and other rheumatological conditions; however, these disorders are better identified through their nonpsychosis clinical features or other investigations. Antinuclear antibody (ANA) is a nonspecific but highly sensitive test for SLE that should be considered when clinically indicated (Wichainun et al., 2013). There may, however, be a role in the future for these markers of inflammation as prognostic or treatment biomarkers (Halstead et al., 2023).

Primary syphilis infection rates are rising in Australia, North America and Europe, particularly in women (King et al., 2022; Chen et al., 2023). There has also been recent concern about elevated rates of blood-borne viruses, such as HIV and Hepatitis C, in those with severe mental illness (Hughes et al., 2016). Screening for HIV, Hepatitis C and syphilis should be considered opportunistically, with consent, for all who are sexually active, methamphetamine-using or who inject drugs (Gazzard et al., 2008; Hutto, 2001; Lopez-Castroman et al., 2012). However, as a cause of psychosis, both HIV and syphilis are uncommon and present in the context of broader central nervous system (CNS) manifestations (Nutile, 2021; Singer and Thames, 2016). If syphilis or HIV is suspected as a cause of psychosis, due to known infection, older age of onset, neurological and/or cognitive symptoms, personality or significant affective symptoms, targeted testing with HIV and syphilis serology is required, and CSF testing may be indicated depending on serology results (Hutto, 2001).

Autoimmune encephalitis, in particular anti-*N*-methyl-d-aspartate (NMDA) receptor encephalitis, is an important cause of psychosis. Australian and New Zealand guidelines have previously recommended universal screening for serum NMDA, glutamic acid decarboxylase (GAD) and voltage-gated potassium channel (VGKC) antibodies (Galletly et al., 2016). The risks and benefits for this approach have been discussed elsewhere, with new recommendations not to pursue universal screening but rather for clinical assessment to be guided by decision-making tools, to detect higher probability presentations that should proceed with serum antibody testing and further to this consideration of paired CSF analysis (Warren et al., 2024a). This approach balances the need for early detection and commencement of alternative treatment with the low prevalence and complexities of serum and CSF antibody testing processes. A current list of recommended antibodies has been reported by Pollak et al. (2020).

#### CSF

Outside of autoimmune encephalitis, CSF analysis should be considered when there are concerns about infectious encephalitis/meningitis or other neurological conditions. A range of CSF parameters may be abnormal in psychosis, indicating alterations to blood-brain barrier function or permeability, as well as neuroinflammation (Warren et al., 2024b). Further research is required to determine their utility in routine assessment.

#### Neuroimaging

The evidence on the use of routine neuroimaging screening in patients presenting with psychosis is divided (Schmidt and Borgwardt, 2020). The clinical benefits need to be weighed against logistical issues, costs, clinical yield and potential harm caused by early exposure to ionising radiation, particularly with computed tomography (CT) (Dorney and Murphy, 2021; Pearce et al., 2012; Schmidt and Borgwardt, 2020). A systematic review assessing the frequency of clinically relevant imaging abnormalities using MRI or CT found that only 2 of 16 international studies had sufficient evidence to support routine imaging in patients with first episode psychosis and only 0.4% of the 2312 patients had an established causality between the abnormality and psychosis (Forbes et al., 2019). Similarly, the American College of Radiology guidelines indicate a low yield in detecting pathology responsible for psychotic symptoms or that leads to a significant change in management in those with no neurological deficit – ranging from 0% to 1.5% for CT and 0% to 2.7% for MRI (Luttrull et al., 2019). In contrast, Blackman et al.’s meta-analysis found the frequency of clinically relevant abnormalities on MRI in first episode psychosis was higher (5.9% pooled prevalence) (Blackman et al., 2023). Of note, Blackman et al.’s definition of clinically relevant abnormalities included structural changes that may have prognostic value but may not change management (Blackman et al., 2023).

Neuroimaging with MRI is recommended in the case of suspected intracranial pathology, with potential clinical features including a history of headaches, nausea/vomiting, seizure-like activity, childhood (<14 years) or older age (>40 years) onset of symptoms, rapid onset or fluctuation of psychosis, significant neurocognitive deficits, catatonia and/or neurological examination findings. For individuals with a first episode psychosis and no atypical features, neuroimaging is less clearly indicated and should be guided by collaborative discussion with the individual and their family/carers, with repeat consideration during future physical health assessments. There may be a future role for advanced neuroimaging, such as functional MRI, in a suite of investigations, to monitor progression of structural and functional changes and guide personalised treatment (Matéos et al., 2023; Soldevila-Matías et al., 2020).

#### EEG

There is evidence for a variety of changes to electroencephalogram (EEG) indices in at-risk-for-psychosis cohorts and in early stages of schizophrenia. These include alterations in gamma and delta power, impaired sensory gating and mismatch negativity (Perrottelli et al., 2021). However, these are not routine clinical EEGs, and results are inconsistent, with no specific indices shown to be a clear marker of transition to psychosis or outcomes (Perrottelli et al., 2021). EEG may be of benefit when there is a history of seizure-like activity or to differentiate cases of encephalitis and delirium from primary psychotic disorders but should not be requested without clinical indication (Wilson et al., 2021).

#### ECG

Clinical indications for ECG include evidence of hypertension or other abnormalities on cardiovascular examination, a history of cardiovascular disease, a family history of arrhythmia/sudden cardiac death and prior to the commencement of specific antipsychotics, such as paliperidone, amisulpride, olanzapine, droperidol and sertindole (Elsayed et al., 2021; Pringsheim et al., 2017). Given the broad-ranging indications and the challenges of conducting an ECG in the community, a baseline ECG during presentation or initial admission is recommended (Philip and Dratcu, 2021).

## Discussion

Globally, the recommended assessments and investigations to identify organic psychoses and examine baseline physical health are heterogeneous. Guidance around physical examination was frequently omitted, and many schizophrenia guidelines focused on specific concerns, such as metabolic syndrome. Although the more recent guidelines provided greater physical health considerations. This perhaps reflects the complexity in assessing the extensive list of organic psychoses, the majority of which are rare disorders that rarely have psychotic symptoms, particularly in the absence of encephalopathy, focal neurological or cognitive deterioration. Although this should not lessen the importance of being aware of these differentials, especially when taking into account the acute and long-term risks of misdiagnosis, such as pharmacotherapy-induced metabolic syndrome and movement disorders, stigma and self-stigma (Carbon et al., 2018; Dubreucq et al., 2021; Rojo et al., 2015). In addition, when considering investigations, careful application is required, as opposed to indiscriminate ordering. In acute settings, often medical stability and physical assessment focused on ensuring safe mental health admission are prioritised. A subsequent detailed assessment, inclusive of relevant collateral from family/carers and external health practitioners, should be conducted by psychiatric staff when allowable during the initial episode of care (Table 3), and this should guide further investigations.

**Table 3.** Initial minimum physical health assessment.

**Medical history:** - Past/current medical diagnoses, family history, development (motor and cognitive), neurological symptoms (gait, seizure-like episodes, abnormal movements, sensory change and migraine), systemically unwell/recent illness (rapid weight change, cough/flu like illness, palpitations, fever and headache), lifestyle (inactivity, nutrition, oral health and smoking/substances) and sexual health **Physical examination:** vitals, anthropometrics, neurology, cardiovascular, endocrine and eye exam **Baseline investigations:** - FBC, Electrolytes, Renal function, Liver function, Prolactin, HbA1c and Lipids - Urine drug screen - ECG - Syphilis, HIV and Hepatitis C serology for anyone sexually active, using methamphetamines or people who inject drugs - Pregnancy test for anyone who could be pregnant - Consider MRI brain + Very early onset of psychosis or history of significant developmental delay or intellectual impairment: neuropsychology assessment, MRI, consider karyotyping and genetic screen, metabolic disorder testing + Late onset of psychosis or significant history of cognitive decline: cognitive assessment, syphilis serology, MRI, consider CSF + Atypical psychosis (catatonia, rapid onset^a^, fluctuating/episodic, cognitive deficits^b^, neurological symptoms/signs, early or excessive EPSE, systematically unwell): serum neuronal antibodies^c^, EEG, MRI, consider CSF (plus serum paired CSF neuronal antibodies) + Occupational exposure (construction/manufacturing/mining/farming): consider heavy metal testing

a Rapid onset: ⩽3 months.

b Cognitive deficits: attention, learning and memory, executive functions.

c Neuronal antibodies: NMDA, LGI1, CASPR2, consider further AMPAR, GABA_A_R, GABA_B_R, Hu, Ma2, CRMP5, DR2, DPPX, MGluR5.

There may be a benefit in considering physical health assessment differently depending on age. It is estimated that around 60% of new psychosis in older persons (over 60–65 years) is associated with a nonprimary psychotic cause, although this is inclusive of affective psychosis presentations (Reinhardt and Cohen, 2015). In particular, delirium and dementia are key contributing factors, and a workup for these should occur for anyone presenting for the first time over the age of 40 years (Seritan, 2023). Continued consideration of such after initial presentation is also important with an increased rate of developing dementia after psychosis, which is highest in the year following diagnosis (Kørner et al., 2009; Stafford et al., 2023). Peri-menopause and menopause may influence presentation and treatment of psychosis, and this should be taken into account during assessment (Nombora et al., 2024). Higher rates of organic causes for psychosis have been seen in children with very early onset of psychosis (⩽12 years of age), with up to 12.5% of first episode psychosis presentations being caused by epilepsy, head trauma, neoplastic disease, autoimmune, inborn errors of metabolism and other genetic disorders (Giannitelli et al., 2018; Merritt et al., 2020). Assessing for developmental delays, intellectual and learning difficulties, dysmorphic features, cardiac defects and neurological deficits will guide the need for karyotyping and other specific testing (Addington et al., 2017).

There may also be a benefit in identifying clinical presentations that are atypical for, or less common in, primary psychotic disorders. German guidelines provide a set of symptoms that indicate increased risk of an organic pathology, which are similar to those in clinical screening tools for autoimmune encephalitis and adapted to those in Figure 1 (DGPPN e.V., 2019; Warren et al., 2020). What is most important is continuing to be aware of abnormal symptoms or physical examination findings and following through when these are identified, including maintaining communication with general practitioners and allied health care professionals (McBride, 2016). Later reconsideration of organic differential diagnoses in people with more established psychosis should occur in the setting of refractory symptoms, new or worsened physical health, especially neurological concerns, cognitive decline and greater than expected extrapyramidal symptoms. Difficulties in professional and patient interaction, including impaired health literacy, stigma, communication challenges, motivation, cognitive deficits and symptoms such as aggression and paranoia, may impact both initial comprehensive assessment and continued attendance for review and further investigations (Mitchell et al., 2009). This complexity may draw attention away from also being alert to physical health care needs, a likely common occurrence, with a recent survey indicating that only 20% of individuals are asked about their physical health by their mental health clinician (Kaine et al., 2022). Ideally, physical health should be considered at every psychiatric appointment.

There are limitations to consider with reference to these reviews and recommendations. Categorisation of pathologies and investigation types was necessary to make this a useful resource, but it may oversimplify information. The systematic review of guidelines contained those focused on a first presentation of psychosis, as well as more established schizophrenia, and this may have impacted the physical health assessment detailed. All were considered for inclusion, as the aim of this study was the most complete understanding of global guidelines. The recommendations here are intended as a guide, and the importance of medical expertise in conducting individualised assessments cannot be overemphasised. These recommendations are not intended to be an algorithm or set of rules to be followed dogmatically, but rather serve to guide the clinician’s thinking about physical health (Greenhalgh et al., 2014). People’s presentations and views may change over time, and investigations may need to be revisited. The individual’s unique presentation and wishes, and those of family, carers and kin should be foremost. The indicated investigations should not be used as a barrier to providing treatment in the most appropriate environment.

Although a structured and systematic approach was taken to assess the evidence behind the recommendations, frameworks such as Appraisal of Guidelines for Research Evaluation (AGREE II) were not applied, as it was felt that most identified guidelines focused on pharmacotherapy and other treatment recommendations and that scoring would not reflect their section on physical health assessment. The recommendations here are consensus expert opinion informed by a range of background evidence. Further work to strengthen these recommendations should occur, such as a Delphi consensus and testing of these recommendations against current practice. Finally, as these recommendations were developed in Australia and New Zealand, this context should be considered, as there may be resource allocation and differences in organic psychoses and comorbidity prevalence elsewhere.

## Conclusion

The exclusion of organic differential diagnoses from initial nonspecific screening and insensitive investigation results without comprehensive clinical assessment can lead to false reassurance and failure to consider treatable medical causes of psychosis. Yet ensuring that individually tailored care occurs in complex and busy clinical systems can be challenging and relies on evidence-driven, structured processes that should be led and reviewed by psychiatrists. These recommendations may be used to guide appropriate physical health assessment for those individuals with an initial presentation of psychosis. This includes a tailored clinical history and physical examination, baseline bloods to assess organ and metabolic function, prolactin and key infectious disease, ECG, urine drug screen (UDS) and consideration of an MRI brain. Other investigations should follow clinical criteria. Such a physical health assessment is critical in both excluding pertinent organic causes and identifying potential physical comorbidities.

## Supplemental Material

sj-docx-1-anp-10.1177_00048674261435740 – Supplemental material for Initial physical health assessment for psychosis in Australia and New Zealand: 2026 recommendations Supplemental material, sj-docx-1-anp-10.1177_00048674261435740 for Initial physical health assessment for psychosis in Australia and New Zealand: 2026 recommendations by Nicola Warren, Cullen O’Gorman, Frances Dark, Susanna Every-Palmer, Sean Halstead, Nicole Korman, Julia Lappin, Sharon Lawn, Brian O’Donoghue, Shuichi Suetani, Andrew Thompson, Samantha M. Loi, James G. Scott, Naomi Runnegar, Toby Pillinger, Robert A. McCutcheon, Graham Blackman, Thomas Skerlj, Jessemin Firman and Dan Siskind in Australian & New Zealand Journal of Psychiatry
