# Supplementary material for: Initial physical health assessment for psychosis in Australia and New Zealand: 2026 recommendations
Source: Aust N Z J Psychiatry. 2026 Apr 26;60(6):575–88. doi: 10.1177/00048674261435740 (PMC13191067; doi:10.1177/00048674261435740)
Supplement: sj-docx-1-anp-10.1177_00048674261435740 – Supplemental material for Initial physical health assessment for psychosis in Australia and New Zealand: 2026 recommendations [file sj-docx-1-anp-10.1177_00048674261435740.docx]

**Appendices**

1. **Complete methodology**
2. **Organic Psychoses**
3. **Type of Evidence used in Organic Psychoses Scoping Review**
4. **Included and Excluded Guidelines**
5. **Included Guideline findings**

**Appendix 1: Complete Methodology**

Initial physical health assessment for individuals presenting with psychosis recommendations were developed by an authorship group with expertise in psychiatry and medical specialties including neurology and infectious disease. Sub-speciality expertise in child and adolescent, youth, rehabilitation, older persons and neuropsychiatry was included. The authorship group contained experts with lived and living experience of mental health. The group also contained psychiatry trainee and junior doctor members. Declaration of conflicts of interest were made with nil identified impacting this work.

*Scoping Review of Organic Psychoses*

The Preferred Reporting Items for Systematic Review and Meta-Analyses extension for scoping reviews (PRIMSA-ScR) were followed [1]. The protocol for this scoping review was not pre-registered. PubMed, Embase, and CINAHL were searched to identify relevant studies from inception to January 2025 using the terms: “organic” OR “secondary” OR “medical” OR “physical” OR “health” AND “psychosis” OR “psychotic” OR “schizophrenia” OR “schizoaffective” OR “schizophreniform” OR “delusional disorder” OR “delusion” OR “hallucination”, limited to reviews. Search and screening at title, abstract and full text was performed independently by two authors (NW, CO) and any divergence resolved by the senior author. Reference lists of included articles were also searched for additional studies.

Studies were included if they contained a list or discussion of organic psychoses. For the purpose of this review organic psychoses was defined as any physical health disorder or condition that resulted in psychotic symptoms at any point in the course of illness. Substance intoxication, withdrawal and induced psychosis were not included. Other psychiatric disorder causes of secondary psychosis were not included.

Data extracted included: physical health disorder/condition, prevalence or incidence of the condition in the general population, rate of psychosis occurring within the condition, presenting features of psychosis, differentiating physical health symptoms or signs, relevant history, examination and investigations for the condition. Where this information was not contained within the identified studies, further literature review on the individual cause of organic psychoses was conducted with prioritisation of data based on hierarchy of evidence (e.g. meta-analysis > systematic review > large cohort) and recency of publication.

Narrative analysis was conducted. Organic psychoses were clustered depending on pathology, with the acknowledgement that some disorders may cross categories, e.g. vascular dementia may be considered both a vascular and neurodegenerative disorder. Some disorders were inclusive of multiple rare conditions e.g. migraine inclusive of familial hemiplegic migraine and undifferentiated migraine. Expert guidance was sought to assist with such to allow for utility and understandability of the results. For the purpose of display, rarity of a disorder was based on Orphanet categorisation of rare diseases, with a disorder typically classified as rare if prevalence was less than 5/10, 000 persons.

*Systematic Review of Psychosis and Schizophrenia Guidelines*

The Preferred Reporting Items for Systematic Review and Meta-Analyses (PRISMA) guidelines were followed [2]. The protocol for this systematic review was not pre-registered. PubMed, Embase, and CINAHL were searched to identify relevant studies from January 2000 to January 2025 using the terms: “psychosis” OR “psychotic” OR “schizophrenia” OR “schizoaffective” OR “schizophreniform” OR “delusional disorder” AND “guideline” OR “recommendation” OR “consensus”. This time period was chosen to capture the most recent guidelines. Search and screening at title, abstract and full text was performed independently by a pair of two authors (TS, JF, NW, CO) and reference lists of included articles were also searched for additional studies. Gray literature search was performed using databases – Agency for Healthcare Research and Quality and handsearching of psychiatric association webpages. Any divergence was resolved by the senior author. No language restrictions applied, and where possible, translations in English were located for screening. Duplicates were reviewed and removed.

Studies were included if there was evidence of a guideline, recommendation or consensus statement for any physical health assessment of schizophrenia spectrum disorders (SSD). SDD included any diagnoses of first-episode psychosis (FEP), schizophreniform, schizophrenia, schizoaffective and delusional disorder. Physical health assessment was considered to be for the purpose of excluding medical causes of psychosis, assessment for common comorbid physical health conditions and/or baseline measures prior to commencement of antipsychotics.

Opinion pieces without guidelines and assessment of guidelines were excluded. Guidelines for physical health of broader psychiatric conditions or presentations, not specific to psychosis, were excluded. Where the same group or country had produced multiple guidelines, all were initially included to allow comparison over time, with the data from the most recent considered for final analysis. Guidelines that made general recommendations without identifying specific assessments, were initially included but removed from final analysis.

Data extraction was completed independently by pairs of two authors (TS, JF, NW, CO) with any divergence resolved by the senior author. For all eligible studies, data extracted included the following variables: location and date, authorship group or body, recommendations for physical health screening (inclusive of physical examination and vitals, bloods, cerebrospinal fluid (CSF), urinalysis, neuroimaging, electrocardiogram, electroencephalogram (EEG), beta- human chorionic gonadotropin (BHCG). Recommendations for specific processes were collected such as fasting status or timing of investigations. Whether the investigation was recommended as routine for all presentations or only when clinically indicated was also noted. If a modality preference for a specific investigation was indicated, then this was also noted, such as in the case of neuroimaging.

Descriptive statistics and narrative synthesis were utilised to report on the findings.

Final recommendations were devised through authorship group review of the results and consensus opinion.

**References**

1. Tricco, A.C., et al., *PRISMA Extension for Scoping Reviews (PRISMA-ScR): Checklist and Explanation.* Ann Intern Med, 2018. **169**(7): p. 467-473.

2. Page, M.J., et al., *PRISMA 2020 explanation and elaboration: updated guidance and exemplars for reporting systematic reviews.* BMJ, 2021. **372**: p. n160.

**Appendix 2. Organic Psychoses**

**Epilepsy:** Lifetime prevalence of epilepsy is thought to be around 7/1000 persons, higher in low to middle income countries [3], and the prevalence of psychosis in epilepsy is around 5.6% [4]. Individuals with schizophrenia have a 2-3 fold increased risk of developing epilepsy [5]. When there has been recent seizure activity, a known diagnosis of epilepsy or concurrent anti-epileptic medications, a possible association is more straightforward and should be directly discussed with neurology. It is uncommon for psychotic symptoms to be the first presentation of epilepsy or occur without overt seizure activity [6, 7]. Pre-ictal psychosis or aura will be of rapid onset and episodic, lasts for minutes to hours, and most commonly includes perceptual disturbance associated with dissociative symptoms, anxiety and euphoria [6, 8]. Ictal psychosis in focal status epilepticus with impaired consciousness, can present similarly to schizophreniform psychosis with perceptual disturbance, although usually this has a rapid onset, is episodic in nature and occurs in combination with other identifying symptoms such as confusion, perplexity, inaccessibility, anxiety, agitation and automatisms [6, 7, 9]. Psychosis may also be associated with anti-epileptic medications.

**Demyelinating disorders:**

Multiple Sclerosis (MS): Prevalence varies with latitude and ranges from 5-370/100, 000 (Australia 96/100, 000) and around 3X more common in females, with onset typically between 20-40 years [10, 11]. Neurological symptoms vary from blurred vision, optic pain, partial myelitis, focal peripheral or central sensory disturbance. The prevalence of psychosis in MS is reported at 4.3% [12], mean age 34.4 years with female predominance [13]. There are 33 case reports of psychosis preceding or at the time of MS diagnosis with a mean delay to MS diagnosis of 2.7 years (SD 3), all with a poor response to antipsychotics [14].

Neuromyelitis optica (NMO): Global prevalence is between 0.3-10/100, 000 persons with female preponderance, onset typically in middle age with optic neuritis and transverse myelitis [15]. There are case reports of psychosis occurring in the context of acute relapse with rapid onset of catatonia, perceptual disturbance, affective and neurocognitive symptoms [16, 17].

Anti-MOG (Myelin oligodendrocyte glycoprotein) antibody associated disease: May present with encephalitis, transverse myelitis, or optic neuritis. Prevalence thought to be between 0.5-3.4/100, 000 persons with a high rate of comorbid autoimmune disorders (including NMDA encephalitis) [18]. There are case reports (3) of psychosis occurring in the context of an acute encephalitis presentation [19-22].

Acute disseminated encephalomyelitis (ADEM): Rare immune mediated inflammatory disorder that can follow infection or vaccination, most commonly post-viral infections such as measles and therefore more common in children [23]. Typically presentation is with gait disturbance, seizures, headache, other motor or cranial nerve deficits or a broader encephalopathy [23]. There are case reports (10) of ADEM presenting with psychotic symptoms, with acute onset of behavioural disturbance, agitation, perceptual disturbance with associated cognitive deficits and rapid progression of other neurological symptoms [24].

**Autoimmune/Inflammatory:**

Autoimmune Encephalitis: The most common autoimmune encephalitis is anti-NMDA receptor encephalitis with an estimated incidence of 1.5/million/year; this is also the most common autoimmune encephalitis that can result in psychotic symptoms, seen in 40-60% of cases [25, 26]. Typically, individuals will present with a rapid onset of psychiatric symptoms and will have associated cognitive, speech, movement disorder, seizures and autonomic dysfunction [26]. Isolated psychosis, without neurological deterioration, is rare and thought to occur in between 0-5% of NMDA encephalitis cases [27]. Other autoimmune antibodies that can present with encephalitides and psychosis include: CASPR2, LGI1, AMPA, GABA, GAD, Hu, Ri, Yo, CV2/CRMP5, Ma2 (Ta), Ampiphysin, DPPX, mGluR5, Neurexin-3-alpha, IgLON5, Glycin-R [25]. These disorders are very rare and psychosis is a less common symptom of their presentation. Many of these antibodies may occur as part of a paraneoplastic syndrome and should be considered in someone presenting with psychosis and a relevant cancer diagnosis. CSF is required for diagnosis.

Antibody negative Autoimmune Encephalitis is a recent construct for clinically suggestive presentations of encephalitis without demonstration of antibodies, or only serum antibody positivity, although significant caution should be applied due to high risk of over and mis-diagnosis [28]. This may be considered with rapid onset psychosis in association with other atypical features such as catatonia, neurological symptoms, autonomic features and cognitive deficits [29, 30]. Repeated antibody testing, MRI, further CSF analysis and EEG are recommended [29].

Systemic Lupus Erythematosus (SLE): The peak age of onset is 40-70 years, more common in females, with incidence higher in people of Black ethnicity [31]. The highest prevalence is reported in North America of 241/100, 000 people [31]. Psychosis secondary to CNS involvement is seen in SLE cases between 1.5 - 11.3% [32-35]. Psychosis may be seen on first presentation or early in the course of illness, however, the majority have other neurologic, systemic or cognitive symptoms or have atypical psychotic features such as rapid or episodic onset [32-35]. ANA and anti-phospholipid antibodies may assist diagnosis, whereas CSF and MRI are frequently normal [32, 34, 35]. Steroid use for SLE can also result in psychosis [32].

Sjogren’s syndrome: Prevalence demonstrated at 60.8/100, 000 persons, typically presenting with slowly progressive oral and eye dryness in middle aged women, but can have other systemic features such as joint pain and fatigue [36]. There are case reports of psychosis with acute onset in the context of confusion, neurological symptoms, cognitive, agitation and behavioural disturbance that have improved with immune suppression, although in general affective and anxiety symptoms are more commonly seen in this disorder than psychosis [37-39].

Antiphospholipid syndrome: Rare disorder seen in 10-15% of women with recurrent miscarriage (recurrent miscarriage occurs in 1% of the population), 40% of those with SLE and 20% of those with stroke prior to the age of 50 years [40]. Typical presentation is with thrombosis (DVT, stroke, migraine) and fertility concerns, less commonly with seizures, vascular dementia and cardiac disease [40]. There are case reports (23) with psychosis, often with affective symptoms, catatonia, neurological and cognitive symptoms, as well as, fever and systemic symptoms [41]. SLE associated with 30% of these cases.

Neurosarcoidosis: Prevalence of sarcoidosis is 2-160/100, 000 persons, although only 2-10% of those will develop neurosarcoidosis [42]. There are case reports of psychosis (delusions, hallucinations, catatonia) in association with neurological and cognitive symptoms, as well as systemic symptoms (respiratory most common) [43-46].

**CNS Infections:**

Acute infectious (viral, bacterial, fungi, parasitic) encephalitis and meningitis: The incidence and type of infectious encephalitis varies on location, time of year, and population (e.g. immunocompromised) but thought to be between 1.4-13.8/100, 000 cases/year for encephalitis and 0.5-207/100, 000 for meningitis, peaking in infants and older persons [47, 48]. Herpes simplex virus type 1 and varicella zoster virus are the most common causes, with presentations consistent with rapid onset of neurological symptoms (headache, focal deficits, seizures, gait disturbance, disturbance of consciousness), neurocognitive symptoms, and systemic features such as fever and malaise [47]. There are multiple case reports of rapid onset of psychosis occurring in the context of acute or recent HSV encephalitis (neurological and cognitive concurrent symptoms), some also in the context of seizure and/or associated anti-NMDA encephalitis [49]. There are also case reports of psychosis occurring with EBV, CMV, influenza, coxsackie, Eastern equine, SarsCoV, Japanese encephalitis [50-52], bacterial encephalitis [53], including mycoplasma pneumoniae [54-56] and tuberculosis [57, 58], fungal (cryptococcal) [59, 60] and parasitic infections (neurocysticercosis, malaria, toxoplasmosis) [61, 62].

Prion disease: Most prion disease is Creutzfeldt-Jakob disease (around 90% occurring sporadically, sCJD) with an incidence of 1-1.5 people per million per year [63]. In those with sCJD, 36.9% have psychotic symptoms at some stage during their illness course and 0.78-3.1% at initial presentation, most commonly with visual perceptual phenomena [64, 65]. Cases with psychosis, early in the illness course, frequently had concurrent sleep disturbance, personality change, neurocognitive symptoms, speech, vision and movement deficits. Delusions were noted less frequently in large cohorts and, when present, tended to be simple in nature [64]. The likelihood of hallucinations increase with continued cognitive impairment [64].

Neuroborreliosis/Lyme disease: incidence of Lyme disease varies by location (United States of America – 18.9/100, 000/year), of which around 12% will have neuroborreliosis [66]. There are case reports of psychosis occurring in this context, with a wide variety of psychotic symptoms, as well as rash, arthralgia, neurological symptoms consistent with subacute/chronic meningitis or encephalomeningitis and history of a recent tick bite in endemic areas [67-72]. Endemic areas include northeast and upper Midwest United States of America and Scandinavian countries [66].

Syphilis: The incidence of syphilis primary infection varies globally, with rates rising in Australia to around 6.7/100, 000/year, and globally since late 1990s [73]. Tertiary syphilis (that occurs 5-20 years after primary infection) occurs in 10-15% of cases, and of those around 20% will develop neurosyphilis, the risk greater with associated HIV infection [74]. There are multiple case reports of psychosis (delusions and hallucinations) occurring with neurosyphilis, typically in association with neurologic and cognitive (confusion, disorientation, memory) symptoms [75, 76].

HIV: There has been a 46% decline in HIV notifications since 2013 in Australia, with 555 new diagnoses in 2022 [73]. Rarely (case reports) psychosis can serve as a presenting manifestation of HIV infection with rapid onset and associated affective and behavioural disturbance and cognitive impairment [77-79]. Psychosis in those with known HIV occurring at rates between 0.23-15.2% [77]. In those with known HIV diagnosis, new onset psychosis most commonly occurs with paranoid delusions, hallucinations, catatonia and associated cognitive impairment, which may be related to a range of pathology from HIV encephalopathy, opportunistic infection, subcortical neurodegeneration, CNS lymphoma, associated antineuronal antibodies or from antiretrovirals [77, 80, 81].

Subacute sclerosis panencephalitis (post measles): Rare disorder with an incidence varying from 0.01-21 cases/million/year, higher in measles endemic areas, presenting in children (mean 8-12 years) typically with myoclonus and progressive cognitive symptoms [82, 83]. There are case reports (15) of psychosis as part of the presentation and in a 10 year cohort there were 20 patients incorrectly diagnosed as having a psychotic disorder prior to this diagnosis [82, 84].

**Neurovascular:**

Intracranial hypertension: There may be multiple causes for intracranial hypertension such as haemorrhage and tumours and there are case reports of psychosis as the predominant symptom, although headache, visual changes, confusion and other cognitive symptoms co-occur [85, 86]. However, there were no clear case reports found for psychosis occurring in the context of idiopathic intracranial hypertension [87-89].

Normal pressure hydrocephalus (NPH): The prevalence ranges from 10-22/100, 000 persons, although increased to 5.9% in those over 80 years for idiopathic NPH [90]. Cohort studies and systematic review have demonstrated a 3X increased rate of psychotic disorders (3.1%) in idiopathic NPH compared to the general population [91]. These cases were in older adults and most commonly had concurrent impaired gait, cognition and urinary continence [91].

Acute stroke: The prevalence of stroke is around 1-3% of the population globally, the majority over the age of 50 and the majority with ischaemic stroke (87%), although 10-15% of stroke occurs in those aged 18-50 years [92]. Systematic review has estimated the prevalence of delusions at 4.7% (most commonly persecutory) and hallucinations at 5.1% (most common auditory) post stroke [93]. Most cases were within the first few days following ischaemic stroke in older adults [93]. The majority presented with concurrent neurological and neurocognitive symptoms, but there were a small number (21 cases) with sudden onset isolated psychosis and no neurological symptoms [93]. There is minimal literature on long term incidence of stroke with one study, finding a psychosis incidence of 6.7% in the 12 years following stroke [94].

Chronic subdural haematoma: Overall incidence is 1.7-20.6/100, 000/year, more common in elderly, typically presenting with headache nausea/vomiting, seizures and acute neurology [95]. There are case reports of psychosis as a prominent symptom in association with neurologic and cognitive symptoms, affective or disorganised behaviour and history of recent trauma/injury [96-98].

Vasculitis: Vasculitis covers multiple different uncommon disorders, that can affect any age group (Kawasaki: young children, Giant Cell Arteritis: older persons) and small, medium and large vessels (also includes polyarteritis nodosa, Behçet’s disease, ANCA associated) [99]. There are case reports with psychosis as a presenting feature, typically acute in onset with associated neurological, headache and cognitive features, as well as, systemic symptoms of vasculitis [100-103].

Arterio-venous malformation: Most AV malformations are asymptomatic, with a detection rate of symptomatic lesions thought to occur at 0.94/100, 000/year, typically presenting with haemorrhage [99]. There are case reports with associated psychosis, particularly prominent hallucinations, in association with behavioural, headache, neurologic and cognitive symptoms [104-106]. Psychosis may be related to a lesional effect or with seizures [107, 108].

Migraine: The best estimate for global prevalence is 14-15%, acknowledging there are also 200 other headache disorders [109]. There are case reports of visual and auditory hallucinations and other phenomena, such as reduplicative phenomena in associated with headache, confusion and temporary neurological symptoms that are reported more frequently in familial hemiplegic migraine than other types of migraine [110-114]. Less commonly, may also occur with migraine aura prior to headache [115].

**CNS lesions:**

Tumours/Neoplasm: There are over 100 types of brain and other CNS tumours with an incidence estimated at 12.7/100, 000/year and 7.4/100, 000/year for CNS malignancies [116]. Most brain tumours will present with neurological symptoms or signs due to site of tumour or mass effect, but there are case reports (30) that have described tumours where there is a presentation of psychosis [117]. Most cases with psychosis will still have concurrent or soon to develop focal or generalised neurological symptoms [118]. The tumours were found in cerebral cortical, pituitary, pineal and posterior locations [118].

Traumatic Brain Injury: Incidence estimates for United States of America are 506.4/100, 000/year (although varies based on source e.g. hospital admission, ED presentation) and severity and cause (injury, surgery, infection) varies significantly [119]. It is estimated that around 40% of those who survive TBI hospitalisation will have a related disability at 1 year [119]. The risk of developing long term psychotic disorders, compared to the general population, is thought to be increased two-fold, with incident rate of psychosis post head injury varying from 0.7-20% [120-126]. Risk of psychosis is greater when injury is severe, diffuse and/or involves the frontal and temporal lobes [121]. Most commonly presents with persecutory delusions, auditory hallucinations, less common to have negative symptoms [120]. Studies have shown that up to half the patients with psychosis also have seizures and up to 70% with EEG abnormalities [120]. The time from TBI to onset of psychosis varies widely, with around 50% developing psychosis within the first two years with a range of 0-34 years [120].

Malformation of cortical development: This group of rare disorders includes disorders of abnormal neurogenesis, neuronal migration and migration arrest, with clinical presentations that vary widely, ranging from mild to severe neurological deficits, with both genetic and in-utero environment causes [127]. Typically, presentation is with seizures, neurologic, developmental and learning difficulties, but there are a minority of case reports of psychosis as an associated symptom with neurological features [128, 129]. Of note, some classification systems include tuberous sclerosis in this group of disorders, listed below, and given the frequency of focal cortical dysplasia with epilepsy, consider the earlier description also.

Porencephaly: Rare disorder with cystic CNS formations that can develop congenitally (3.5/100, 000 live births) or post infection, haemorrhage or trauma neonatally, typically presenting with developmental delay, neurological and cognitive features [130]. There are case reports (5) of psychosis, associated with cysts in frontal and limbic areas, often including malformations of cortical development [131].

**Delirium:** Delirium is common but prevalence varies significantly dependent on setting and population group, for example, estimated at 23% of medical inpatients and between 4-38% of those in nursing homes [132]. Delusions (9-70%) and hallucinations (25-70%) are common symptoms in delirium, more frequent with hyperactive delirium [133-135]. Visual hallucinations are more common than auditory and tactile [135]. Delusions are most frequently paranoid in nature [134, 135]. Causes are variable, including: major organ derangement, fluid and electrolyte disturbance, hypo/hyperglycaemia and infections.

**Neurodegenerative/Neurocognitive:**

Huntington’s Disease: The prevalence of Huntington’s is thought to be around 5-10/100, 000 persons [136]. Large Huntington’s disease cohorts indicate prevalence of psychosis is between 5-18% [136-140]. Patients with psychosis tend to have less chorea but are of younger age with worse cognitive, functional and behavioural disturbance, than those with no psychosis [137, 138]. They also have a higher rate of family members with psychosis [139]. Psychosis may occur at the time of neurological symptom onset or many years later but not typically before, whereas, mood, anxiety and personality change symptoms may pre-date neurological features [136, 139, 140]. Psychosis is often accompanied by cognitive and affective symptoms [136]. Around 10% of Huntington’s patients have juvenile HD (before age 20 years) and are thought to have higher rates of seizures, dystonia, parkinsonism and cognitive disturbance – there have been rare case reports of psychosis within this cohort [141, 142].

Alzheimer’s Disease: Alzheimer’s is the most common dementia, with 3% of those aged 65-74 year affected, rising to 50% in those over 85 years [143]. Psychotic symptoms typically occur in middle stages and may no longer be communicated in more advanced stages, occurring in around 41% of patients [144, 145]. Delusions (15-30% of individuals with Alzheimer’s) are typically of paranoid, money/theft, somatic, misidentification type and may occur in disease of mild severity [146, 147]. Hallucinations (5-15% of individuals) are predominately visual in nature but auditory can also occur [133, 147]. Hallucinations are more common with mixed Alzheimer’s and Lewy Body Dementias [148]. Psychosis is associated with agitation, more severe impairment and rapid decline in cognitive function [145, 147].

Parkinson’s Disease: Parkinson’s Disease prevalence increases with age, from 41/100, 000 at 40-49 years, to 1.1% at 70-79 years, affecting higher rates of males at younger ages [149]. Psychosis occurs in around 3% at the time of diagnosis, 10% at 2 years and within 12 years up to 60% of those with Parkinson’s Disease have had some psychotic symptom [150]. The overall pooled prevalence of psychosis is 20.7% [151]. There are much higher rates of psychosis in those with Parkinson’s Disease Dementia and more severe Parkinson’s, but psychosis still occurs in those without cognitive decline. Typically, visual hallucinations occur earlier in the disease course, as well as passage and presence phenomena [150]. In later stages of Parkinson’s, non-visual hallucinations and delusions occur [150].

Lewy Body Disease: There is considerable overlap with Parkinson’s Disease Dementia, with Lewy Body Dementia accounting for 15-20% of people with late onset dementia and with an estimated prevalence of 4.2-7.5% of the population [152, 153]. Between 30-50% of those with Lewy Body Dementia have neuroleptic sensitivity, and over 80% have complex, well-formed and detailed visual hallucinations [152]. Other common psychotic symptoms include misidentification, reduplication and presence hallucinations/delusion [154]. Features most frequently associated with psychosis include fluctuating cognition, variation in attention and alertness, REM sleep behaviour disorder, parkinsonism, sleep changes, dysautonomia [152].

Vascular dementia: Vascular dementia, inclusive of cortical and subcortical multi-infarct, hypoperfusion and hereditary types (CADASIL), accounts for up to 15% of dementias, with risk of developing such doubling every 5.3 years [155]. Large cohorts indicate rates of psychosis at around 37-46% with variable rates of delusions, hallucinations and delusional misidentification [155, 156]. Persecutory delusions are common and visual hallucinations were associated with increased cognitive decline [156]. Concurrent neurological and cognitive deficit were present.

Amyotrophic lateral sclerosis (ALS/Motor Neuron Disease) overlaps with frontotemporal dementia (FTD): The prevalence of ALS is 4.2/100, 000 typically incidence peaking at ages 60-70 years, where as FTD is seen to affect 15-22/100, 000 persons and is the second most common early onset dementia [157, 158]. There is emerging evidence that these two disorders share a common pathology and genetics and are now considered to be a continuum of overlapping diseases [159]. Psychosis is more common in those with C9orf72 expansion (less common with GRN and MAPT mutations), may occur years prior to motor or cognitive features, and is more common with poorer cognition ?at baseline [160, 161]. Psychotic symptoms are common in ALS (10-22%), FTD (24- 39%) and ALS/FTD overlap (55%) [160-163]. Both delusions and hallucinations are seen, with somatic based delusions noted more commonly [160]. Literature review indicates up to 4-6% of those later diagnosed with FTD, were initially treated for schizophrenia (aged 28-43 years), with an average of 10 years prior to FTD diagnosis [164]. Psychosis prior to ALS diagnosis is less common and typically occurs in the context of significant motor decline [165, 166].

**Endocrine:**

Hyperthyroidism: The prevalence of overt hyperthyroidism (in iodine sufficient parts of the world) is between 0.2-1.3%, with Grave’s disease being the most common cause [167]. There are case reports of psychosis and hyperthyroidism (seen less commonly than with hypothyroidism), most commonly with associated affective symptoms, confusion/delirium like picture and systemic features of thyrotoxicosis [168-170].

Hypothyroidism: Iodine deficiency and Hashimoto’s thyroiditis are the most common causes of primary hypothyroidism, which affects 1-2% globally, increasing to 7% over the age of 85 years [167]. Psychotic symptoms have been shown in up to 15% of individuals with hypothyroidism, however, the physical symptoms associated with thyroid dysfunction and a predominant mood and/or anxiety disturbance typically occur in the weeks to months prior [171-173].

Hashimoto’s thyroid disease encephalitis, Graves disease encephalitis, Encephalopathy associated with autoimmune thyroid disease (EAAT, sometimes called SREAT – steroid responsive): rare disorder (around 250 case reports) that may have euthyroid status (or sub clinically elevated or reduced) with high circulating levels of anti-thyroid antibodies, presents with subacute confusion, headache, neurological symptoms, affective and cognitive and rarely psychosis [174-177]. Systematic review identified 61 cases with persecutory delusions [178].

Hypoparathyroidism: The prevalence of hypoparathyroidism is estimated at 22-37/100, 000 persons and may result from surgery, autoimmune and rarely genetic causes [179]. Psychosis is a rare occurrence (case reports) with significant hypocalcaemia (fatigue, muscle cramps, tetany, seizures, arrythmias, gastrointestinal disturbance) in the setting of a delirium like picture [180-182].

Hyperparathyroidism/ and other causes of hypercalcaemia: Primary hyperparathyroidism is common, with a prevalence of 233/100, 000 in women and 85/100, 000 in men [183]. The most common psychiatric associated symptom is depression and confusion but there are multiple case reports/series of psychosis, typically paranoid or catatonic with concurrent neurocognitive symptoms [184-186]. Fatigue, disturbance of sleep, gastrointestinal, renal and skeletal symptoms may be present [185]. In general, the level of mental state disturbance is correlated with calcium level, although cases of psychosis have been present with mild hypercalcaemia [187].

Cushing’s syndrome with excess adrenal glucocorticoid: Incidence of Cushing’s Syndrome ranges between 1.8-3.2/million/year, with around 60% caused by Cushing’s Disease [188]. Central weight gain, abdominal striae, easy bruising, facial plethora, muscle atrophy, headache, hypertension and other cardiovascular concerns, electrolyte disturbance are the most common symptoms [189]. Psychiatric concerns are typically affective and confusion, less commonly (case reports) there is psychosis described [190]. Large systematic reviews and cohorts indicate psychotic symptoms in around 8% of cases, more common with adrenal carcinomas [191, 192]. Psychosis is typically associated with sleep disturbance, affective symptoms, confusion and other neurocognitive symptoms [189, 190].

Addison’s disease/adrenal insufficiency: Primary adrenal insufficiency prevalence ranges from 0.4-22/100, 000 people and secondary from 14-28/100, 000 and acute adrenal crisis will occur in around 11/100 persons/year [193]. Psychosis is not common (compared to excess glucocorticoid states) but there are a few case reports of psychosis as an initial presenting symptom in the context of an Addisonian crisis [194-197]. Associated affective symptoms, sleep disturbance, confusion and other neurocognitive symptoms are frequently seen. Other symptoms may include weakness, fatigue, weight loss, muscle/joint pain, anorexia, hypotension and electrolyte disturbance [195].

Phaeochromocytoma and paraganglioma: Incidence of these rare disorders range from 0.04-0.95/100, 000/year [198]. There are case reports of psychosis, episodic in nature with associated hypertension, headache, palpitations and anxiety [199].

**Genetic:**

Down Syndrome: Down Syndrome occurs in 1.2:1000 pregnancies of which around 78% are live births, with a prevalence in adults around 5.9/10, 000 [200]. Rates of psychosis have been seen in between 13-43% of cohorts, often associated with depressed mood, increasing with age and dementia [201]. Although, there are large cohorts with no described psychosis, so the true rate is unclear, and may be confounded by difficulties in assessment [200]. There are also high rates of catatonia, not always associated with clear mood or psychotic features [202].

Fragile X Syndrome: 1:2000-4000 males and 1:4000-8000 females will have the full mutation and be affected, typically with no dysmorphic facial features but may have macro-orchidism, epilepsy, intellectual impairment, ASD and ADHD [203]. There are case reports of psychosis with catatonia and other behavioural disturbance in males [203-205].

Klinefelter syndrome: Found in 1:660 males, thought to be under diagnosed and often diagnosed late in life with hypogonadism, gynaecomastia, infertility, diabetes, seizures, tremor, hypotonia, psychomotor delay (language > motor), academic difficulties, ADHD and BPAD [206]. Swedish population based studies have demonstrated a 3.6 times higher rate of schizophrenia than general and a psychosis prevalence of 12% seen in large cohort studies [207].

Turner syndrome: Incidence ranges from 1:2000 to 1:2500 live born females/year, typically with short stature, failure or reduced secondary sexual characteristics, neck webbing, hair loss, heart defects, kidney defects and learning difficulties [208]. Swedish national register studies have demonstrated a 1.98 X the risk of psychosis [208]. Women with schizophrenia have a 3 fold increase in Turner’s syndrome especially higher risk of mosaic 45X, 46XX [209, 210].

Retinitis pigmentosa: Prevalence is between 1:3000-7000 presenting typically with progressive deterioration in visual function (night blindness first) and may include syndromes such as Usher’s Syndrome (associated with bilateral sensorineural hearing loss) [211]. Case reports of psychosis have been reported, especially higher in those with Usher’s Syndrome, where up to 23% of some cohorts have psychosis [211-213]. Since there are D4 receptors in retina rods, care should be taken with antipsychotic choice [214].

22q11.2 deletion/Di George/Velocardiofacial Syndrome: Prevalence is estimated at 1:1600 persons [215]. There is a 20 fold increased risk of psychosis, with psychosis seen in 30-40% and around 25% diagnosed with schizophrenia [216-218]. Given the greater rate of cardiac arrhythmias, seizures and movement disorders influencing pharmacotherapy choice, karyotyping should be considered with a history of developmental delay, dysmorphic facial features, cleft palate, hypernasal speech, cardiac defects, intellectual or learning difficulties [219, 220]. There is also an increased risk of ADHD and bipolar disorder [215].

15q11-q13 deletion/Prader-Willi Syndrome: Estimated to occur in 1:10, 000-15, 000 live births characterised by mild to moderate intellectual disability, obesity associated with hyperphagia, hypogonadism, hypotonia, epilepsy, higher rates of ASD and ADHD [221]. Psychosis is thought to occur in between 9-60%, more common with chromosome 15 maternal uniparental disomy (mUPD) genetic sub-type [222]. Psychosis most commonly presents with an acute onset of hallucinations and delusions accompanied by affective symptoms, as well as, confusion, anxiety and catatonic motor symptoms, occurring in an episodic nature [222].

7q11.23 deletion/Williams-Beuren syndrome: Rare disorder (1:20, 000 live births) with facial dysmorphic features (elfin like), cardiovascular, connective tissue and endocrine abnormalities, anxiety, distinctive cognitive pattern (severe visuospatial deficit, strength in vernal memory, language and music) [223]. There are case reports of associated psychosis, with larger reviews reporting a rate of 5-6% [223-225].

Other Copy Number Variation (CNV) associated genomic disorders that are rare and with possible association with psychosis include: Deletion at 1q21.1, 2p 16.3 (NRXN1), 3q29, 15q11.2, 15q13.3, 16p12.1, 16p11.2, 17p12 (HNPP), 17q12 and duplication at 1q21.1, 7q11.23, 15q11-q13, 16p13.11, 16p11.2  [226-230]. Typically there is associated intellectual impairment, developmental delay, dysmorphic features, cardiac defects and seizures [230].

Corpus callosum agenesis (including Andermann Syndrome): Rare (less than 1 per million) disorder, with some case series indicating higher rates of psychosis (20 cases/62) as a feature in association with intellectual impairment, developmental delay, epilepsy, dysmorphic features and neurologic deficits [231].

Friedreich’s ataxia: Estimated prevalence is between 1-4/100, 000 typically presenting with neurological (cerebellar and sensory ataxia), cardiac (hypertrophic cardiomyopathy) and skeletal abnormalities (scoliosis), with many having impaired glucose tolerance and visual and hearing loss [232]. There are case reports of psychosis (18% of 175 patients after 20 years disease duration) typically in the setting of a long history of the disorder with visual, hearing, tactile and proprioceptive loss [232, 233].

Primary familial brain calcification/Fahr’s disease: Rare disorder (<1/million prevalence) that typically occurs after the age of 40 years and presents with neuropsychiatric and movement symptoms. Most commonly extrapyramidal symptoms will be the first presentation of the disease, but may also have associated cognitive and mood symptoms with psychiatric features seen in about 40% of cases [234, 235]. Psychotic symptoms, seen in only a few case reports, include auditory and visual hallucinations, perceptual distortions, delusions and fugue state [234, 236].

Tuberous Sclerosis: Estimate prevalence is between 6.8 and 12.4/100, 000 people, typically with presentations of epilepsy as well as dermatological, cardiac, renal and pulmonary manifestations and higher rates of autism (40-50%), ADHD (30-50%) and other behavioural disturbance termed TAND (Tuberous-sclerosis Associated Neuropsychiatric Disease) [237, 238]. Psychosis occurs about the same frequency as the general population but there are higher rates of affective and anxiety disorders and psychosis may also relate to seizures [237].

Amyloidosis including Hereditary transthyretin (ATTR) amyloidosis: Rare disorders (1/100, 000 persons) characterised by multiorgan involvement – typically cardiac, liver and renal [239]. Leptomeningeal amyloidosis may present with psychotic symptoms but these are most often in association with cognitive, neurological, intracranial haemorrhage and other somatic symptoms [239, 240].

Mitochondrial disorders including MELAS: These are rare disorders (1-12/100, 000 but difficult to define epidemiology accurately) with variable clinical presentations that may include short stature, cardiomyopathy, stroke like episodes, hearing loss, type 1 diabetes [241, 242]. There are case reports of psychosis associated with progressive dementia and other neurological or acute cognitive symptoms such as seizures [241, 243].

Megalencephalic leukoencephalopathy/Van der Knaap disease: This rare slowly progressive neurodegenerative disorder of children present with cerebellar ataxia, seizures and muscle spasticity. There is a case report of psychosis in someone with known disorder, with microencephaly and then neurological and cognitive symptoms [244]. Of note, other leukodystrophies, with associated psychosis, are included under inborn errors of metabolism.

Inborn errors of metabolism (IEM): rare disorders that are most often identified in newborn screening, early in neonatal period or with a family history. Commonly there is intellectual impairment, neurological symptoms, cardiac defects, liver and other major organ impacts, hypotonia and eye disease. There are cases reports of psychosis in these disorders, typically in the setting of mild disease, late onset or episodic following exposure or an event, most often associated with an encephalopathic presentation:

- Urea cycle disorders, including Ornithine transcarbamylase (OTC) deficiency [245-247]
- Maple syrup urine disease [248]
- Neuroferritinopathy [249, 250]
- Propionic acidemia [251]
- Cobalamin C disease: late onset may just have neurologic and psychiatric features [252]
- Lysosomal storage disorders, including [253]:
  - Tay-Sachs disease: in late onset type (1.5-42 years) psychiatric signs may be present for many years prior to onset of neurological deterioration with motor decline, seizures, choreiform movement and dementia [254]. Psychosis is reported in 30-50% of cases, often misdiagnosed with schizophrenia, but associated dysarthria and progressive speech loss, as well as, regression in behaviour was often also present [254]. Cases with psychosis frequently had transient symptoms and were not improved with antipsychotics [254].
  - Metachromatic leukodystrophy: There are a higher number of case reports (129) with about 50% of those with the disorder reporting some psychotic symptoms typically also in association with intellectual impairment and further progressive cognitive decline [255-257]. This is a rate much higher than with other IEM that have neurological complications and thought to be related to frontal white matter disease.
  - Niemann-Pick Disease Type C: case reports of psychosis often associated with agitation, sleep disturbance, hyperactivity and affective symptoms [258-260]. In a large case series, 2/52 cases of Niemann-Pick disease had an initial psychosis presentation 5 and 15 years prior to diagnosis, both associated with significant sleep and neurological symptoms and had significant sensitivity to antipsychotics [258]. In a systematic review of cases, 62% had psychosis at some point during their illness [261].
  - Gaucher disease: case reports of psychosis have been described [262-264]. In systematic review psychosis is noted often in association with Parkinsonism and Lewy Body Dementia, but a prevalence not given [265].
  - α-Mannosidosis: case reports of psychosis occurring in association with intellectual impairment [266, 267].
  - Neuronal ceroid lipofuscinoses: case reports (2/27 in case series) of psychosis (typically visual hallucinations) with intellectual impairment, behavioural and sleep disturbance and neurocognitive deterioration have been reported, although affective and anxiety symptoms are more common [268].
- X-Adrenoleukodystrophy late onset: There are case reports of psychosis, often with affective symptoms and neurological (abnormal movements) [269, 270].
- CerebroTendinous Xanthomatosis: Case series of psychosis, typically in association with dementia, neurological symptoms (pyramidal and cerebellar, seizures), cardiovascular, osteoporosis, cataracts and tendon xanthomas have been described [271, 272].
- Porphyrias: acute and episodic psychosis can occur (case reports), although mood and anxiety disturbance are more common [273-275]. Abdominal pain occurs in 90-95% of attacks, with peripheral neuropathy and autonomic features also frequently reported, and there is often a triggering event/exposure such as recent illness or medication [273, 275].
- Wilson’s disease: Rare autosomal recessive disorder with rates of psychosis that vary from 1.4-11.3% and can occur prior to or after other symptoms, which include movement disorder, neurocognitive features and signs of liver impairment/failure [276]. Cases with psychosis often have high rates of associated mood, anxiety and OCD with extra sensitivity to antipsychotics [276]. Kayser-Fleischer rings occur in 90-100% of neurologic patients [277]. Low ceruloplasmin is not sensitive to the disease and thus screening via such is not recommended [278].
- Phenylketonuria: newborn screening has been present since 1963, but there are subsequent cohorts of psychosis in the context of exposure in known disorder [279-281]. Even with early treatment, there are noted higher rates of intellectual impairment, ADHD, anxiety and mood disturbance [279-281].

**Nutritional:** Of note, nutritional deficiencies are often multi-nutritional**.**

B12/Cobalamin deficiency: Psychosis described in case reports with paranoia and perceptual disturbance, in association with neurocognitive changes, delirium and neurological symptoms [252, 282]. This may occur in vegans, pernicious anaemia, post gastric surgery, use of metformin and PPIs or various inborn errors of cobalamin metabolism [252]. Severe deficiency that may result in neurological concerns is uncommon, however subclinical deficiency affects between 2.6% and 26% of the population, varying with geographic location and age [283].

Pellagra (Niacin/B3 deficiency): niacin deficiency is rare in developed countries (may occur in context of chronic alcoholism, chronic inflammatory conditions or antiepileptic medications) and presents with gastrointestinal symptoms (diarrhoea), dermatitis and cognitive deterioration. Psychosis described in case reports associated with encephalopathy [284, 285]

Wernicke-Korsakoff (Thiamine/B1 deficiency): Prevalence is estimated at 0.4-2.8%, with recent population wide studies demonstrating incidence of Wernicke’s at 1.2(female) to 3.7 (male)/100, 000/year [286]. Symptoms of Wernicke encephalopathy include loss of appetite, gastrointestinal symptoms, cerebellar symptoms, fatigue, diplopia, nystagmus and ophthalmoplegia, insomnia, peripheral neuropathy, anxiety, cognitive symptoms (episodic memory) and seizures [287, 288]. There may also be associated history of alcoholism, anorexia and other chronic nutritional disorders. The classic triad of signs (confusion, gait ataxia and ophthalmoplegia) do not occur in many patients [289]. Psychotic symptoms can occur acutely, typically hallucinations [288]. With ongoing or untreated chronic deficiency Korsakoff syndrome will occur in between 56-84% with irreversible cognitive symptoms (memory, executive, confabulation, apathy) and psychosis can again occur in this context, estimated at around 10% of cases [286, 287, 290]. Often there is associated mood disturbance and agitation [290].

**Toxin:** Heavy metals exposure: Heavy metal exposure can in a range of settings such as industrial, mining, fertiliser production and from paint, contaminated soil and herbal product exposure. Typically, there will be encephalopathy and a range of neurologic and cognitive symptoms. There are case reports of psychosis occurring from toxin exposure to:

- Cyanide [291]
- Manganese [292, 293].
- Arsenic [294]
- Mercury [295, 296].
- Toluene and other solvents [297, 298]
- Lead [299, 300]

**Medications**: A range of medications can result in psychosis including: [301-303]

- Anti-Parkinson’s: levodopa, bromocriptine, amantadine
- Anticholinergics (including tricyclics)
- Histamine blockers: cimetidine
- Anti-cancer agents: cisplatin, cyclophosphamide,
- Anti-epileptics: phenytoin, carbamazepine
- Indomethacin
- Opioid analgesics
- Corticosteroids
- Digoxin
- Antiarrhythmics
- Interferon
- Isoniazid
- Bupropion

**Other:**

**Narcolepsy:** The prevalence is thought to be 20-50/100,000 globally, most commonly diagnosed in 20s, with approximately 36% with narcolepsy type 2 (no cataplexy) [304]. Systematic review indicates psychotic symptoms are common in narcolepsy, with an increased comorbidity with schizophrenia, a psychotic form of narcolepsy with vivid REM sleep behaviour disturbance rationalised in a delusional manner, and visual hallucinations/illusions at the sleep-wake transition [305]. There is typically overt sleep disturbance, daytime sleepiness and cataplexy [305]. Most common psychotic symptom are hallucinations occurring in around 80% of those with narcolepsy, most hypnogogic/hypnopompic and mostly a combination of auditory/visual/tactile [306].

**Kleine-Levin Syndrome:** Rare disorder with around 186 case reports in the literature, mostly of adolescent men with recurrent episodes of hypersomnia, cognitive symptoms (confusion), binge eating or increased craving for food, abnormal speech, mood disturbance, and within this context less commonly there are delusions, hallucinations and dissociative symptoms [307]. There is frequently a trigger such as systemic infection or head injury [307].

**High altitude cerebral oedema:** Cerebral oedema occurs at altitudes >2500m within 1-5 days, typically proceeded by acute mountain sickness, with a prevalence of <0.2% in general mountaineering population [308]. Psychosis can occur as an isolated symptom with hallucinations and/or delusions, related to hypoxia and/or cerebral oedema [309].

**References**

1. Fiest, K.M., et al., *Prevalence and incidence of epilepsy: a systematic review and meta-analysis of international studies.* Neurology, 2017. **88**(3): p. 296-303.

2. Clancy, M.J., et al., *The prevalence of psychosis in epilepsy; a systematic review and meta-analysis.* BMC Psychiatry, 2014. **14**: p. 75.

3. Wotton, C.J. and M.J. Goldacre, *Coexistence of schizophrenia and epilepsy: record-linkage studies.* Epilepsia, 2012. **53**(4): p. e71-4.

4. Agrawal, N. and M. Mula, *Treatment of psychoses in patients with epilepsy: an update.* Ther Adv Psychopharmacol, 2019. **9**: p. 2045125319862968.

5. Mula, M., *Epilepsy-induced behavioral changes during the ictal phase.* Epilepsy Behav, 2014. **30**: p. 14-6.

6. Perven, G. and N.K. So, *Epileptic auras: phenomenology and neurophysiology.* Epileptic Disorders, 2015. **17**(4): p. 349-362.

7. Elliott, B., E. Joyce, and S. Shorvon, *Delusions, illusions and hallucinations in epilepsy: 2. Complex phenomena and psychosis.* Epilepsy Research, 2009. **85**(2): p. 172-186.

8. Forouhari, A., et al., *Multiple sclerosis epidemiology in Asia and Oceania; A systematic review and meta-analysis.* Multiple Sclerosis and Related Disorders, 2021. **54**: p. 103119.

9. Pugliatti, M., S. Sotgiu, and G. Rosati, *The worldwide prevalence of multiple sclerosis.* Clinical neurology and neurosurgery, 2002. **104**(3): p. 182-191.

10. Marrie, R.A., et al., *The incidence and prevalence of psychiatric disorders in multiple sclerosis: a systematic review.* Mult Scler, 2015. **21**(3): p. 305-17.

11. Camara-Lemarroy, C.R., et al., *The varieties of psychosis in multiple sclerosis: A systematic review of cases.* Mult Scler Relat Disord, 2017. **12**: p. 9-14.

12. Sabe, M. and O. Sentissi, *Psychotic symptoms prior or concomitant to diagnosis of multiple sclerosis: a systematic review of case reports and case series.* Int J Psychiatry Clin Pract, 2022. **26**(3): p. 287-293.

13. Papp, V., et al., *Worldwide incidence and prevalence of neuromyelitis optica: a systematic review.* Neurology, 2021. **96**(2): p. 59-77.

14. Alam, A., et al., *Neuromyelitis optica presenting with psychiatric symptoms and catatonia: a case report.* General Hospital Psychiatry, 2015. **37**(3): p. 274.e1-274.e2.

15. Moore, P., et al., *Cognitive and psychiatric comorbidities in neuromyelitis optica.* Journal of the Neurological Sciences, 2016. **360**: p. 4-9.

16. Hor, J.Y. and K. Fujihara, *Epidemiology of myelin oligodendrocyte glycoprotein antibody-associated disease: a review of prevalence and incidence worldwide.* Frontiers in Neurology, 2023. **14**: p. 1260358.

17. Zhou, J., et al., *An unusual case of anti-MOG CNS demyelination with concomitant mild anti-NMDAR encephalitis.* J Neuroimmunol, 2018. **320**: p. 107-110.

18. von Zedtwitz, K., et al., *Anti-MOG autoantibody-associated schizophreniform psychosis.* Acta neuropsychiatrica, 2022. **34**(1): p. 47-54.

19. Zahid, A., et al., *First Episode Psychosis and Status Epilepticus as Presenting Symptom of FLAIR-hyperintense Lesions in Anti-MOG-associated Encephalitis with Seizures (FLAMES).* Neurology, 2024. **103**(7_Supplement_1): p. S47-S47.

20. Cheng, X., et al., *Anti-Myelin Oligodendrocyte Glycoprotein Antibody-Associated Meningitis with Psychotic Symptoms.* Authorea Preprints, 2024.

21. Li, K., et al., *Clinical presentation and outcomes of acute disseminated Encephalomyelitis in adults worldwide: systematic review and meta-analysis.* Frontiers in Immunology, 2022. **13**: p. 870867.

22. Nasr, J.T., M.R. Andriola, and P.K. Coyle, *Adem: literature review and case report of acute psychosis presentation.* Pediatric Neurology, 2000. **22**(1): p. 8-18.

23. Endres, D., et al., *Autoimmune encephalitis as a differential diagnosis of schizophreniform psychosis: clinical symptomatology, pathophysiology, diagnostic approach, and therapeutic considerations.* European archives of psychiatry and clinical neuroscience, 2020. **270**: p. 803-818.

24. Dalmau, J., et al., *An update on anti-NMDA receptor encephalitis for neurologists and psychiatrists: mechanisms and models.* The Lancet Neurology, 2019. **18**(11): p. 1045-1057.

25. Kayser, M.S., et al., *Frequency and characteristics of isolated psychiatric episodes in anti–N-methyl-d-aspartate receptor encephalitis.* JAMA Neurol, 2013. **70**(9): p. 1133-9.

26. Dalmau, J. and F. Graus, *Diagnostic criteria for autoimmune encephalitis: utility and pitfalls for antibody-negative disease.* The Lancet Neurology, 2023. **22**(6): p. 529-540.

27. Steiner, J., et al., *Autoimmune encephalitis with psychosis: Warning signs, step-by-step diagnostics and treatment.* The World Journal of Biological Psychiatry, 2020. **21**(4): p. 241-254.

28. Graus, F., et al., *A clinical approach to diagnosis of autoimmune encephalitis.* Lancet Neurol, 2016. **15**(4): p. 391-404.

29. Rees, F., et al., *The worldwide incidence and prevalence of systemic lupus erythematosus: a systematic review of epidemiological studies.* Rheumatology, 2017. **56**(11): p. 1945-1961.

30. Appenzeller, S., F. Cendes, and L.T.L. Costallat, *Acute psychosis in systemic lupus erythematosus.* Rheumatology international, 2008. **28**: p. 237-243.

31. Pego-Reigosa, J.M. and D.A. Isenberg, *Psychosis due to systemic lupus erythematosus: characteristics and long-term outcome of this rare manifestation of the disease.* Rheumatology, 2008. **47**(10): p. 1498-1502.

32. Abrol, E., et al., *Psychosis in systemic lupus erythematosus (SLE): 40-year experience of a specialist centre.* Rheumatology, 2021. **60**(12): p. 5620-5629.

33. Hanly, J.G., et al., *Psychosis in systemic lupus erythematosus: results from an international inception cohort study.* Arthritis & rheumatology, 2019. **71**(2): p. 281-289.

34. Qin, B., et al., *Epidemiology of primary Sjögren's syndrome: a systematic review and meta-analysis.* Annals of the rheumatic diseases, 2015. **74**(11): p. 1983-1989.

35. Hammett, E.K., et al., *Adolescent Sjogren’s syndrome presenting as psychosis: a case series.* Pediatric Rheumatology, 2020. **18**: p. 1-8.

36. Pt, S., et al., *Primary Sjogren's syndrome presenting as acute psychosis with catatonic symptoms.* Indian Journal of Psychiatry, 2022. **64**(Suppl 3).

37. Lin, C.-E., *One patient with Sjogren’s syndrome presenting schizophrenia-like symptoms.* Neuropsychiatric Disease and Treatment, 2016: p. 661-663.

38. Ruiz-Irastorza, G., et al., *Antiphospholipid syndrome.* The Lancet, 2010. **376**(9751): p. 1498-1509.

39. Hallab, A., et al., *Association of psychosis with antiphospholipid antibody syndrome: A systematic review of clinical studies.* General Hospital Psychiatry, 2018. **50**: p. 137-147.

40. Rossides, M., et al., *Sarcoidosis: Epidemiology and clinical insights.* Journal of internal medicine, 2023. **293**(6): p. 668-680.

41. Van Hoye, G., et al., *Case report: Psychosis with catatonia in an adult man: a presentation of neurosarcoidosis.* Frontiers in Psychiatry, 2024. **15**: p. 1276744.

42. Blanco, L.S., et al., *Psychotic disorder of organic etiology, in the context of sarcoidosis. A case report.* European Psychiatry, 2017. **41**(S1): p. s836-s837.

43. Bona, J.R., et al., *Neurosarcoidosis as a cause of refractory psychosis: a complicated case report.* American Journal of Psychiatry, 1998. **155**(8): p. 1106-1108.

44. Celebi, A., et al., *A case of isolated neurosarcoidosis associated with psychosis.* Neurosciences Journal, 2013. **18**(1): p. 70-73.

45. Duerlund, L.S., H. Nielsen, and J. Bodilsen, *Current epidemiology of infectious encephalitis: a narrative review.* Clinical Microbiology and Infection, 2024.

46. Zunt, J.R., et al., *Global, regional, and national burden of meningitis, 1990–2016: a systematic analysis for the Global Burden of Disease Study 2016.* The Lancet Neurology, 2018. **17**(12): p. 1061-1082.

47. Oommen, K.J., P.C. Johnson, and C.G. Ray, *Herpes simplex type 2 virus encephalitis presenting as psychosis.* The American Journal of Medicine, 1982. **73**(3): p. 445-448.

48. Torrey, E.F., *Functional psychoses and viral encephalitis.* Integrative Psychiatry, 1986. **4**(4): p. 224-230.

49. Ariza-Varón, M., et al., *Psychosis associated with suspected SARS-CoV-2 encephalitis with response to steroids: a case report.* Infectious Diseases, 2022. **54**(2): p. 152-156.

50. Monnet, F.P., *Behavioural disturbances following Japanese B encephalitis.* European Psychiatry, 2003. **18**(6): p. 269-273.

51. Hsu, T.-W., et al., *Risk of Major Mental disorder after severe bacterial Infections in children and adolescents: a Nationwide Longitudinal Study.* Neuropsychobiology, 2022. **81**(6): p. 539-549.

52. Banerjee, B. and K. Petersen, *Psychosis Following Mycoplasma Pneumonia.* Military Medicine, 2009. **174**(9): p. 1001-1004.

53. Cohen, M. and A. Jay, *Psychosis in a Case of Mycoplasma Pneumonia Encephalopathy.* Jefferson Journal of Psychiatry, 1986. **4**(2): p. 10.

54. Sterner, G. and G. Biberfeld, *Central nervous system complications of Mycoplasma pneumoniae infection.* Scandinavian Journal of Infectious Diseases, 1969. **1**(3): p. 203-208.

55. Rahim, M.J. and W.S. Ghazali, *Psychosis secondary to tuberculosis meningitis.* BMJ Case Rep, 2016. **2016**.

56. Gunawardena, S.M., E. McMahon, and A.M. Doherty, *A systematic review of the prevalence of psychosis in people with tuberculosis.* European Psychiatry, 2024. **67**(S1): p. S370-S370.

57. Sa'adah, M., et al., *Cryptococcal meningitis and confusional psychosis. A case report and literature review.* Tropical and geographical medicine, 1995. **47**(5): p. 224-226.

58. Michielsen, P. and M. Arts, *Cryptococcal Meningitis in Acute Onset Psychosis: A Case-Study.* European Psychiatry, 2016. **33**(S1): p. S532-S532.

59. ElemamaliI, A., S. Talyb, and A. Awad, *Neurocysticercosis presenting with psychosis.* New Horizons in Clinical Case Reports, 2017. **1**: p. 23.

60. Carpio, A., et al., *Parasitic diseases of the central nervous system: lessons for clinicians and policy makers.* Expert Rev Neurother, 2016. **16**(4): p. 401-14.

61. Chen, C. and X.-P. Dong, *Epidemiological characteristics of human prion diseases.* Infectious diseases of poverty, 2016. **5**: p. 1-10.

62. Thompson, A., et al., *Behavioral and psychiatric symptoms in prion disease.* American Journal of Psychiatry, 2014. **171**(3): p. 265-274.

63. Appleby, B.S., K.K. Appleby, and P.V. Rabins, *Does the presentation of Creutzfeldt-Jakob disease vary by age or presumed etiology? A meta-analysis of the past 10 years.* J Neuropsychiatry Clin Neurosci, 2007. **19**(4): p. 428-35.

64. Koedel, U., V. Fingerle, and H.-W. Pfister, *Lyme neuroborreliosis—epidemiology, diagnosis and management.* Nature Reviews Neurology, 2015. **11**(8): p. 446-456.

65. Mattingley, D.W. and M.M. Koola, *Association of Lyme Disease and Schizoaffective Disorder, Bipolar Type: Is it Inflammation Mediated?* Indian J Psychol Med, 2015. **37**(2): p. 243-6.

66. Bar, K.-J., et al., *Painful hallucinations and somatic delusions in a patient with the possible diagnosis of neuroborreliosis.* The Clinical journal of pain, 2005. **21**(4): p. 362-363.

67. Pasareanu, A.R., Å. Mygland, and Ø. Kristensen, *A woman in her 50s with manic psychosis.* Tidsskrift for Den norske legeforening, 2012.

68. Hess, A., et al., *Borrelia burgdorferi central nervous system infection presenting as an organic schizophrenialike disorder.* Biological psychiatry, 1999. **45**(6): p. 795.

69. Pfister, H.-W., et al., *Catatonic syndrome in acute severe encephalitis due to Borrelia burgdorferi infection.* Neurology, 1993. **43**(2): p. 433-433.

70. Fallon, B.A. and J.A. Nields, *Lyme disease: a neuropsychiatric illness.* American Journal of Psychiatry, 1994. **151**(11): p. 1571-1583.

71. King, J., et al., *HIV, viral hepatitis and sexually transmissible infections in Australia Annual Surveilance report 2022. The Kirby Institute, UNSW Sydney, Sydney, Australia; 2022*.

72. Nutile, L.M., *Neurosyphilis with psychosis as the primary presentation.* American Journal of Psychiatry Residents' Journal, 2021.

73. Friedrich, F., et al., *Psychosis in neurosyphilis-clinical aspects and implications.* Psychopathology, 2013. **47**(1): p. 3-9.

74. Jarocki, A., et al., *Neurosyphilis-Induced Psychosis in Europe: A Systematic Review of Case Reports.* Pathogens, 2024. **13**(11): p. 959.

75. Nebhinani, N. and S.K. Mattoo, *Psychotic disorders with HIV infection: A review.* German J Psychiatry, 2013. **16**(1): p. 43-48.

76. Sewell, D.D., et al., *HIV-associated psychosis: a study of 20 cases. San Diego HIV Neurobehavioral Research Center Group.* Am J Psychiatry, 1994. **151**(2): p. 237-42.

77. Vogel-Scibilia, S.E., B.H. Mulsant, and M.S. Keshavan, *HIV infection presenting as psychosis: a critique.* Acta Psychiatrica Scandinavica, 1988. **78**(5): p. 652-656.

78. De Ronchi, D., et al., *Psychopathology of first-episode psychosis in HIV-positive persons in comparison to first-episode schizophrenia: a neglected issue.* AIDS care, 2006. **18**(8): p. 872-878.

79. Van Rensburg, B.J. and C. Bracken, *Acute psychiatric in-patients tested for HIV status: A clinical profile.* South African Journal of Psychiatry, 2007. **10**(1).

80. Garg, R.K., et al., *The spectrum of psychiatric manifestations in subacute sclerosing panencephalitis: A systematic review of published case reports and case series.* CNS Spectrums, 2024. **29**(2): p. 87-95.

81. Gutierrez, J., R.S. Issacson, and B.S. Koppel, *Subacute sclerosing panencephalitis: an update.* Developmental Medicine & Child Neurology, 2010. **52**(10): p. 901-907.

82. Prashanth, L., et al., *Subacute sclerosing panencephalitis (SSPE): an insight into the diagnostic errors from a tertiary care university hospital.* Journal of child neurology, 2007. **22**(6): p. 683-688.

83. Alonso, B.L., *Atypical psychosis in a patient with intracranial hypertension: clinical case and review.* European Psychiatry, 2024. **67**(S1): p. S488-S489.

84. Vakis, A.F., et al., *Psychosis-like syndrome associated with intermittent intracranial hypertension caused by a large arachnoid cyst of the left temporal lobe.* British journal of neurosurgery, 2006. **20**(3): p. 156-159.

85. Duggal, H.S., *Idiopathic intracranial hypertension presenting with psychiatric symptoms.* The Journal of Neuropsychiatry and Clinical Neurosciences, 2005. **17**(3): p. 426-427.

86. de Oliveira, M.F., et al., *Psychiatric symptoms are frequent in idiopathic intracranial hypertension patients.* Neurosurgical Review, 2021. **44**(2): p. 1183-1189.

87. Korsbæk, J.J., et al., *Psychiatric comorbidities in patients with idiopathic intracranial hypertension: a prospective cohort study.* Neurology, 2022. **99**(2): p. e199-e208.

88. Zaccaria, V., et al., *A systematic review on the epidemiology of normal pressure hydrocephalus.* Acta Neurologica Scandinavica, 2020. **141**(2): p. 101-114.

89. Vanhala, V., et al., *Prevalence of Schizophrenia in Idiopathic Normal Pressure Hydrocephalus.* Neurosurgery, 2019. **84**(4).

90. Saini, V., L. Guada, and D.R. Yavagal, *Global epidemiology of stroke and access to acute ischemic stroke interventions.* Neurology, 2021. **97**(20_Supplement_2): p. S6-S16.

91. Stangeland, H., V. Orgeta, and V. Bell, *Poststroke psychosis: a systematic review.* Journal of Neurology, Neurosurgery & Psychiatry, 2018. **89**(8): p. 879-885.

92. Almeida, O.P. and J. Xiao, *Mortality associated with incident mental health disorders after stroke.* Australian & New Zealand Journal of Psychiatry, 2007. **41**(3): p. 274-281.

93. Feghali, J., W. Yang, and J. Huang, *Updates in chronic subdural hematoma: epidemiology, etiology, pathogenesis, treatment, and outcome.* World neurosurgery, 2020. **141**: p. 339-345.

94. Kar, S.K., et al., *Psychiatric manifestation of chronic subdural hematoma: the unfolding of mystery in a homeless patient.* Indian Journal of Psychological Medicine, 2015. **37**(2): p. 239-242.

95. Alp, A., et al., *Recurrent Psychotic Depression Associated with Chronic Subdural Haematoma: A Case Report.* Archives of Neuropsychiatry, 2024. **61**(2): p. 180.

96. Singh, T.B., et al., *Chronic subdural haematoma presenting as late onset psychosis.* ASEAN J Psychiatry, 2013. **14**: p. 175-8.

97. Watts, R.A., et al., *Global epidemiology of vasculitis.* Nature reviews rheumatology, 2022. **18**(1): p. 22-34.

98. Fontes, H., et al., *Central nervous system vasculitis presenting as acute psychotic disorder.* European Psychiatry, 2011. **26**(S2): p. 745-745.

99. Gasparinho, R., et al., *Psychosis as the First Manifestation of Granulomatosis with Polyangiitis-A Case Report.* Psychiatria Danubina, 2022. **34**(2): p. 315-317.

100. Latvala, H.M., S.K. Reitan, and A.E. Vaaler, *Giant Cell Arteritis Presenting with Mania, Psychosis, and Cognitive Dysfunction: A Case Report.* Case Reports in Psychiatry, 2023. **2023**(1): p. 7989712.

101. Patel, P., et al., *Neuro-Behçet disease presenting with acute psychosis in an adolescent.* Journal of child neurology, 2014. **29**(9): p. NP86-NP91.

102. Nelson, E.A., et al., *Auditory hallucinations associated with an arteriovenous malformation of the brain.* The Journal of Neuropsychiatry and Clinical Neurosciences, 2022. **34**(1): p. 89-92.

103. Shah, C.C., et al., *Clinically Isolated Syndrome and Frontal Lobe Arteriovenous Malformation Presenting With Behavior Issues.* Journal of Medical Cases, 2022. **14**(1): p. 1.

104. Aleem, A. and M.A. Knesevich, *Schizophrenia-like psychosis associated with vein of Galen malformation: a case report.* The Canadian Journal of Psychiatry, 1987. **32**(3): p. 226-227.

105. Hanine, I., et al., *Seizures, Psychosis, and Cerebral Vascular Malformation: A Rare Chain of Events.* Case Reports in Psychiatry, 2024. **2024**(1): p. 8656715.

106. Villa, N.A.E. and E.D. Espiridion, *Neuropsychiatric Manifestations of Arteriovenous Malformation: A Case of Acute Mania.* Cureus, 2024. **16**(4): p. e58297.

107. Steiner, T.J. and L.J. Stovner, *Global epidemiology of migraine and its implications for public health and health policy.* Nature Reviews Neurology, 2023. **19**(2): p. 109-117.

108. LaBianca, S., et al., *Familial Hemiplegic Migraine and Recurrent Episodes of Psychosis: A Case Report.* Headache, 2015. **55**(7): p. 1004-7.

109. Fuller, G.N., et al., *Migraine madness: recurrent psychosis after migraine.* Journal of Neurology, Neurosurgery & Psychiatry, 1993. **56**(4): p. 416-418.

110. Feely, M., et al., *Episodes of acute confusion or psychosis in familial hemiplegic migraine.* Acta Neurologica Scandinavica, 1982. **65**(4): p. 369-375.

111. Miller, E.E., et al., *Auditory hallucinations associated with migraine: Case series and literature review.* Cephalalgia, 2015. **35**(10): p. 923-930.

112. Spranger, M., et al., *Familial hemiplegic migraine with cerebellar ataxia and paroxysmal psychosis.* European neurology, 1999. **41**(3): p. 150-152.

113. Barros, J., et al., *Psychotic aura symptoms in familial hemiplegic migraine type 2 (ATP1A2).* J Headache Pain, 2012. **13**(7): p. 581-5.

114. Gurney, J.G. and N. Kadan-Lottick, *Brain and other central nervous system tumors: rates, trends, and epidemiology.* Current opinion in oncology, 2001. **13**(3): p. 160-166.

115. Madhusoodanan, S., et al., *Psychiatric aspects of brain tumors: a review.* World journal of psychiatry, 2015. **5**(3): p. 273.

116. Ghandour, F., et al., *Presenting psychiatric and neurological symptoms and signs of brain tumors before diagnosis: a systematic review.* Brain sciences, 2021. **11**(3): p. 301.

117. Corrigan, J.D., A.W. Selassie, and J.A.L. Orman, *The epidemiology of traumatic brain injury.* The Journal of head trauma rehabilitation, 2010. **25**(2): p. 72-80.

118. Fujii, D. and I. Ahmed, *Characteristics of psychotic disorder due to traumatic brain injury: an analysis of case studies in the literature.* J Neuropsychiatry Clin Neurosci, 2002. **14**(2): p. 130-40.

119. Sachdev, P., J.S. Smith, and S. Cathcart, *Schizophrenia-like psychosis following traumatic brain injury: a chart-based descriptive and case-control study.* Psychol Med, 2001. **31**(2): p. 231-9.

120. Kostenniemi, U.J., et al., *Psychiatric disabilities and other long-term consequences of childhood bacterial meningitis.* The Pediatric Infectious Disease Journal, 2021. **40**(1): p. 26-31.

121. Rantakallio, P., et al., *Association between central nervous system infections during childhood and adult onset schizophrenia and other psychoses: a 28-year follow-up.* International journal of epidemiology, 1997. **26**(4): p. 837-843.

122. Dalman, C., et al., *Infections in the CNS during childhood and the risk of subsequent psychotic illness: a cohort study of more than one million Swedish subjects.* American Journal of Psychiatry, 2008. **165**(1): p. 59-65.

123. Leask, S.J., D.J. Done, and T.J. Crow, *Adult psychosis, common childhood infections and neurological soft signs in a national birth cohort.* The British Journal of Psychiatry, 2002. **181**(5): p. 387-392.

124. Khandaker, G.M., et al., *Childhood infection and adult schizophrenia: a meta-analysis of population-based studies.* Schizophrenia research, 2012. **139**(1-3): p. 161-168.

125. Pang, T., R. Atefy, and V. Sheen, *Malformations of cortical development.* The neurologist, 2008. **14**(3): p. 181-191.

126. Ho, C.S.H., et al., *Prevalence of neuropsychiatric symptoms associated with malformations of cortical development.* Epilepsy & Behavior, 2019. **92**: p. 306-310.

127. Kawaguchi, A., et al., *A case of schizophrenia accompanied by lissencephaly.* The Journal of Neuropsychiatry and Clinical Neurosciences, 2013. **25**(4): p. E17-E18.

128. Guerra, M., et al., *Porencephaly in the adult age.* Ann Clin Case Rep, 2021. **6**: p. 1955.

129. Douzenis, A., et al., *Porencephaly and psychosis: a case report and review of the literature.* BMC Psychiatry, 2010. **10**: p. 19.

130. Wilson, J.E., et al., *Delirium.* Nature Reviews Disease Primers, 2020. **6**(1): p. 90.

131. Seritan, A.L., *Advances in the Diagnosis and Management of Psychotic Symptoms in Neurodegenerative Diseases: A Narrative Review.* J Geriatr Psychiatry Neurol, 2023. **36**(6): p. 435-460.

132. Tachibana, M., et al., *Prevalence, clinical features, and risk factors of delusions in patients with delirium.* International Journal of Geriatric Psychiatry, 2022. **37**(6).

133. Webster, R. and S. Holroyd, *Prevalence of Psychotic Symptoms in Delirium.* Psychosomatics, 2000. **41**(6): p. 519-522.

134. Naarding, P., H. Kremer, and F. Zitman, *Huntington's disease: a review of the literature on prevalence and treatment of neuropsychiatric phenomena.* European Psychiatry, 2001. **16**(8): p. 439-445.

135. Rocha, N.P., et al., *The clinical picture of psychosis in manifest huntington's disease: a comprehensive analysis of the Enroll-HD database.* Frontiers in neurology, 2018. **9**: p. 930.

136. Connors, M.H., A. Teixeira-Pinto, and C.T. Loy, *Psychosis and longitudinal outcomes in Huntington disease: the COHORT Study.* Journal of Neurology, Neurosurgery & Psychiatry, 2020. **91**(1): p. 15-20.

137. Tsuang, D., et al., *Familial aggregation of psychotic symptoms in Huntington’s disease.* American Journal of Psychiatry, 2000. **157**(12): p. 1955-1959.

138. Van Duijn, E., E. Kingma, and R. Van Der Mast, *Psychopathology in verified Huntington’s disease gene carriers.* The Journal of neuropsychiatry and clinical neurosciences, 2007. **19**(4): p. 441-448.

139. Ribaï, P., et al., *Psychiatric and Cognitive Difficulties as Indicators of Juvenile Huntington Disease Onset in 29 Patients.* Archives of Neurology, 2007. **64**(6): p. 813-819.

140. Chuo, Y.-P., et al., *Juvenile Huntington's disease presenting as difficult-to-treat seizure and the first episode of psychosis.* General Hospital Psychiatry, 2012. **34**(4): p. 436.e9-436.e11.

141. Sosa-Ortiz, A.L., I. Acosta-Castillo, and M.J. Prince, *Epidemiology of dementias and Alzheimer’s disease.* Archives of medical research, 2012. **43**(8): p. 600-608.

142. Scarmeas, N., et al., *Delusions and hallucinations are associated with worse outcome in Alzheimer disease.* Arch Neurol, 2005. **62**(10): p. 1601-8.

143. Ropacki, S.A. and D.V. Jeste, *Epidemiology of and risk factors for psychosis of Alzheimer’s disease: a review of 55 studies published from 1990 to 2003.* American Journal of Psychiatry, 2005. **162**(11): p. 2022-2030.

144. Kumfor, F., et al., *Examining the presence and nature of delusions in Alzheimer's disease and frontotemporal dementia syndromes.* International Journal of Geriatric Psychiatry, 2022. **37**(3).

145. Devanand, D.P., et al., *Overview of late-onset psychoses.* Int Psychogeriatr, 2024. **36**(1): p. 28-42.

146. Devanand, D.P., et al., *Associations Between Neuropsychiatric Symptoms and Neuropathological Diagnoses of Alzheimer Disease and Related Dementias.* JAMA Psychiatry, 2022. **79**(4): p. 359-367.

147. Pringsheim, T., et al., *The prevalence of Parkinson's disease: a systematic review and meta‐analysis.* Movement disorders, 2014. **29**(13): p. 1583-1590.

148. ffytche, D.H., et al., *The psychosis spectrum in Parkinson disease.* Nature Reviews Neurology, 2017. **13**(2): p. 81-95.

149. Chendo, I., et al., *Frequency and Characteristics of Psychosis in Parkinson's Disease: A Systematic Review and Meta-Analysis.* J Parkinsons Dis, 2022. **12**(1): p. 85-94.

150. Kyle, K. and J.M. Bronstein, *Treatment of psychosis in Parkinson's disease and dementia with Lewy Bodies: A review.* Parkinsonism & Related Disorders, 2020. **75**: p. 55-62.

151. Walker, Z., et al., *Lewy body dementias.* The Lancet, 2015. **386**(10004): p. 1683-1697.

152. Nagahama, Y., et al., *Classification of Psychotic Symptoms in Dementia With Lewy Bodies.* The American Journal of Geriatric Psychiatry, 2007. **15**(11): p. 961-967.

153. O'Brien, J.T. and A. Thomas, *Vascular dementia.* The Lancet, 2015. **386**(10004): p. 1698-1706.

154. Ballard, C., et al., *Anxiety, depression and psychosis in vascular dementia: prevalence and associations.* Journal of affective disorders, 2000. **59**(2): p. 97-106.

155. Onyike, C.U. and J. Diehl-Schmid, *The epidemiology of frontotemporal dementia.* International Review of Psychiatry, 2013. **25**(2): p. 130-137.

156. Xu, L., et al., *Global variation in prevalence and incidence of amyotrophic lateral sclerosis: a systematic review and meta-analysis.* Journal of Neurology, 2020. **267**(4): p. 944-953.

157. Lattante, S., et al., *Defining the genetic connection linking amyotrophic lateral sclerosis (ALS) with frontotemporal dementia (FTD).* Trends in Genetics, 2015. **31**(5): p. 263-273.

158. Devenney, E.M., et al., *Neural mechanisms of psychosis vulnerability and perceptual abnormalities in the ALS‐FTD spectrum.* Annals of Clinical and Translational Neurology, 2021. **8**(8): p. 1576-1591.

159. Chatterjee, A., et al., *A Systematic Review of the Genetics and Pathology of Psychosis in Frontotemporal Dementia.* Canadian Journal of Neurological Sciences, 2023: p. 1-28.

160. Wilcox, A., et al., *Frequency and neural correlates related to psychosis in motor neurone disease.* medRxiv, 2021: p. 2021.03. 31.21253701.

161. Shinagawa, S., et al., *Psychosis in frontotemporal dementia.* Journal of Alzheimer's disease, 2014. **42**(2): p. 485-499.

162. Velakoulis, D., et al., *Frontotemporal dementia presenting as schizophrenia-like psychosis in young people: clinicopathological series and review of cases.* The British Journal of Psychiatry, 2009. **194**(4): p. 298-305.

163. Turner, M.R., et al., *Psychiatric disorders prior to amyotrophic lateral sclerosis.* Annals of neurology, 2016. **80**(6): p. 935-938.

164. Seelen, M., et al., *Prior medical conditions and the risk of amyotrophic lateral sclerosis.* Journal of neurology, 2014. **261**: p. 1949-1956.

165. Taylor, P.N., et al., *Global epidemiology of hyperthyroidism and hypothyroidism.* Nature Reviews Endocrinology, 2018. **14**(5): p. 301-316.

166. Urias-Uribe, L., et al., *Psychosis crisis associated with thyrotoxicosis due to Graves’ disease.* Case reports in psychiatry, 2017. **2017**(1): p. 6803682.

167. Dahale, A.B., et al., *Postpartum psychosis in a woman with Graves' disease: a case report.* General Hospital Psychiatry, 2014. **36**(6): p. 761. e7-761. e8.

168. Brownlie, B.E., et al., *Psychoses associated with thyrotoxicosis - 'thyrotoxic psychosis.' A report of 18 cases, with statistical analysis of incidence.* Eur J Endocrinol, 2000. **142**(5): p. 438-44.

169. Feldman, A.Z., R.T. Shrestha, and J.V. Hennessey, *Neuropsychiatric manifestations of thyroid disease.* Endocrinol Metab Clin North Am, 2013. **42**(3): p. 453-76.

170. Hall, R.C., *Psychiatric effects of thyroid hormone disturbance.* Psychosomatics, 1983. **24**(1): p. 7-11, 15-8.

171. Heinrich, T.W. and G. Grahm, *Hypothyroidism Presenting as Psychosis: Myxedema Madness Revisited.* Prim Care Companion J Clin Psychiatry, 2003. **5**(6): p. 260-266.

172. Haider, A.S., et al., *Autoimmune Schizophrenia? Psychiatric Manifestations of Hashimoto's Encephalitis.* Cureus, 2016. **8**(7): p. e672.

173. Bocchetta, A., et al., *Affective psychosis, Hashimoto's thyroiditis, and brain perfusion abnormalities: case report.* Clinical Practice and Epidemiology in Mental Health, 2007. **3**(1): p. 31.

174. Bharadwaj, B., A. Sugaparaneetharan, and R.P. Rajkumar, *Graves' disease presenting with catatonia: a probable case of encephalopathy associated with autoimmune thyroid disease.* Acta Neuropsychiatrica, 2012. **24**(6): p. 374-379.

175. Menon, V., K. Subramanian, and J.S. Thamizh, *Psychiatric Presentations Heralding Hashimoto's Encephalopathy: A Systematic Review and Analysis of Cases Reported in Literature.* J Neurosci Rural Pract, 2017. **8**(2): p. 261-267.

176. Laurent, C., et al., *Steroid-responsive encephalopathy associated with autoimmune thyroiditis (SREAT): Characteristics, treatment and outcome in 251 cases from the literature.* Autoimmunity Reviews, 2016. **15**(12): p. 1129-1133.

177. Clarke, B.L., et al., *Epidemiology and diagnosis of hypoparathyroidism.* The Journal of Clinical Endocrinology & Metabolism, 2016. **101**(6): p. 2284-2299.

178. GREENE, J.A. and L. Swanson, *Psychosis in hypoparathyroidism; with a report of five cases.* Annals of Internal Medicine, 1941. **14**(7): p. 1233-1236.

179. Nehu Parimi, J.T., et al., *A case of psychosis due to acute hypocalcemia from hypoparathyroidism.* Journal of the Endocrine Society, 2021. **5**(Supplement_1): p. A172-A173.

180. Ang, A.W.-K., S.M. Ko, and C.H. Tan, *Calcium, magnesium, and psychotic symptoms in a girl with idiopathic hypoparathyroidism.* Psychosomatic medicine, 1995. **57**(3): p. 299-302.

181. Minisola, S., et al., *Epidemiology, pathophysiology, and genetics of primary hyperparathyroidism.* Journal of Bone and Mineral Research, 2020. **37**(11): p. 2315-2329.

182. Alarcón, R.D. and J.A. Franceschini, *Hyperparathyroidism and paranoid psychosis: case report and review of the literature.* The British Journal of Psychiatry, 1984. **145**(5): p. 477-486.

183. Park, S. and R. Hieber, *Acute psychosis secondary to suspected hyperparathyroidism: a case report and literature review.* Mental Health Clinician, 2016. **6**(6): p. 304-307.

184. Murphy, R.J., S. Paul, and R. Primelo, *Parathyroid Paranoia: Unveiling Psychosis in Hyperparathyroidism.* Case Reports in Psychiatry, 2024. **2024**(1): p. 8126125.

185. Otsuki, K., et al., *Psychosis in a primary hyperparathyroidism patient with mild hypercalcemia: A case report.* Medicine, 2021. **100**(12): p. e25248.

186. Hakami, O.A., S. Ahmed, and N. Karavitaki, *Epidemiology and mortality of Cushing’s syndrome.* Best practice & research Clinical endocrinology & metabolism, 2021. **35**(1): p. 101521.

187. Mohamed, A.S.M., et al., *Cushing’s disease presenting with psychosis.* Practical Neurology, 2021. **21**(4): p. 351-353.

188. Fujii, Y., et al., *Cushing's syndrome and psychosis: a case report and literature review.* The primary care companion for CNS disorders, 2018. **20**(5): p. 26189.

189. Lin, T.Y., J. Hanna, and W.W. Ishak, *Psychiatric Symptoms in Cushing's Syndrome: A Systematic Review.* Innov Clin Neurosci, 2020. **17**(1-3): p. 30-35.

190. Kelly, W., *Psychiatric aspects of Cushing's syndrome.* QJM: An International Journal of Medicine, 1996. **89**(7): p. 543-552.

191. Husebye, E.S., et al., *Adrenal insufficiency.* The Lancet, 2021. **397**(10274): p. 613-629.

192. Farah, J.d.L., et al., *Severe psychotic disorder as the main manifestation of adrenal insufficiency.* Case Reports in Psychiatry, 2015. **2015**(1): p. 512430.

193. Sanat, Z.M. and M.R. Mohajeri-Tehrani, *Psychotic disorder as the first manifestation of Addison disease: a case report.* International Journal of Endocrinology and Metabolism, 2022. **20**(1).

194. Spiegel, D.R., et al., *A case of psychosis in a patient with secondary adrenal insufficiency: a possible etiological role of a hypocortisolemic-induced increase in proinflammatory cytokines.* Innovations in Clinical Neuroscience, 2017. **14**(9-10): p. 4.

195. McFarland, H.R., *Addison's disease and related psychoses.* Comprehensive Psychiatry, 1963. **4**(2): p. 90-95.

196. Al Subhi, A.R., V. Boyle, and M.S. Elston, *Systematic review: incidence of pheochromocytoma and paraganglioma over 70 years.* Journal of the Endocrine Society, 2022. **6**(9): p. bvac105.

197. Brown, J.S., *Cases of remission of psychosis following resection of pheochromocytoma or paraganglioma.* Schizophrenia Research, 2016. **176**(2): p. 304-306.

198. Mantry, D., et al., *The prevalence and incidence of mental ill‐health in adults with Down syndrome.* Journal of Intellectual Disability Research, 2008. **52**(2): p. 141-155.

199. Dykens, E.M., et al., *Psychiatric disorders in adolescents and young adults with Down syndrome and other intellectual disabilities.* Journal of neurodevelopmental disorders, 2015. **7**: p. 1-8.

200. Ghaziuddin, N., A. Nassiri, and J.H. Miles, *Catatonia in Down syndrome; a treatable cause of regression.* Neuropsychiatric Disease and Treatment, 2015: p. 941-949.

201. Tsiouris, J.A. and W.T. Brown, *Neuropsychiatric Symptoms of Fragile X Syndrome.* CNS Drugs, 2004. **18**(11): p. 687-703.

202. iIndah Winarni, T., et al., *Psychosis and catatonia in fragile X: case report and literature review.* Intractable & Rare Diseases Research, 2015. **4**(3): p. 139-146.

203. Das, P., C.F. Johnston, and S. Hossain, *Schizophrenia in a patient with full mutation of Fragile X gene and intellectual disability: a ‘STEP’towards better understanding.* Psychiatric genetics, 2020. **30**(3): p. 83-86.

204. Groth, K.A., et al., *Klinefelter Syndrome—A Clinical Update.* The Journal of Clinical Endocrinology & Metabolism, 2013. **98**(1): p. 20-30.

205. Cederlöf, M., et al., *Klinefelter syndrome and risk of psychosis, autism and ADHD.* Journal of Psychiatric Research, 2014. **48**(1): p. 128-130.

206. Björlin Avdic, H., et al., *Neurodevelopmental and psychiatric disorders in females with Turner syndrome: a population-based study.* Journal of neurodevelopmental disorders, 2021. **13**: p. 1-9.

207. Prior, T.I., P.S. Chue, and P. Tibbo, *Investigation of Turner syndrome in schizophrenia.* American Journal of Medical Genetics, 2000. **96**(3): p. 373-378.

208. Roser, P. and W. Kawohl, *Turner syndrome and schizophrenia: a further hint for the role of the X-chromosome in the pathogenesis of schizophrenic disorders.* The World Journal of Biological Psychiatry, 2010. **11**(2-2): p. 239-242.

209. McDonald, C., P. Kenna, and T. Larkin, *Retinitis pigmentosa and schizophrenia.* European Psychiatry, 1998. **13**(8): p. 423-426.

210. Waldeck, T., B. Wyszynski, and A. Medalia, *The relationship between Usher's syndrome and psychosis with Capgras syndrome.* Psychiatry, 2001. **64**(3): p. 248-55.

211. Selvakumar, N., et al., *Diagnostic and therapeutic challenges due to psychosis and catatonia in non-syndromic retinitis pigmentosa: A case report.* Indian Journal of Psychological Medicine, 2020. **43**(5): p. 458-459.

212. Salem, H., R. Elkhatib, and T. Pigott, *S246. Psychosis in a Patient With Retinitis Pigmentosa: Beware of D4 Receptor Blockade!* Biological Psychiatry, 2018. **83**(9): p. S443-S444.

213. Shprintzen, R.J., *Velo‐cardio‐facial syndrome: 30 years of study.* Developmental disabilities research reviews, 2008. **14**(1): p. 3-10.

214. Murphy, K.C., L.A. Jones, and M.J. Owen, *High rates of schizophrenia in adults with velo-cardio-facial syndrome.* Archives of general psychiatry, 1999. **56**(10): p. 940-945.

215. Tanham, M., et al., *The effectiveness and tolerability of pharmacotherapy for psychosis in 22q11. 2 Deletion Syndrome: A systematic review.* Australian & New Zealand Journal of Psychiatry, 2024. **58**(5): p. 393-403.

216. Bassett, A.S. and E.W. Chow, *Schizophrenia and 22q11. 2 deletion syndrome.* Current psychiatry reports, 2008. **10**(2): p. 148-157.

217. Gothelf, D., et al., *Clinical characteristics of schizophrenia associated with velo-cardio-facial syndrome.* Schizophrenia research, 1999. **35**(2): p. 105-112.

218. Tanham, M., et al., *The effectiveness and tolerability of pharmacotherapy for psychosis in 22q11.2 Deletion Syndrome: A systematic review.* Aust N Z J Psychiatry, 2024. **58**(5): p. 393-403.

219. Veltman, M.W.M., E.E. Craig, and P.F. Bolton, *Autism spectrum disorders in Prader–Willi and Angelman syndromes: a systematic review.* Psychiatric Genetics, 2005. **15**(4).

220. Aman, L.C.S., et al., *Psychotic illness in people with Prader–Willi syndrome: a systematic review of clinical presentation, course and phenomenology.* Orphanet Journal of Rare Diseases, 2024. **19**(1): p. 69.

221. Aodh, A.M. and A.A. Al-Marshedi, *Williams-Beuren's Syndrome: A Case Report in Prince Sultan Military City, Riyadh, Saudi Arabia 2022.* World Journal of Environmental Biosciences, 2023. **12**(1-2023): p. 20-23.

222. Salgado, H. and L. Martins-Correia, *Williams syndrome and psychosis: a case report.* Journal of Medical Case Reports, 2014. **8**: p. 1-3.

223. Valdes, F., et al., *Brief Report: Major Depressive Disorder with Psychotic Features in Williams Syndrome: A Case Series.* Journal of Autism and Developmental Disorders, 2018. **48**(3): p. 947-952.

224. Rees, E., et al., *Analysis of copy number variations at 15 schizophrenia-associated loci.* The British Journal of Psychiatry, 2014. **204**(2): p. 108-114.

225. Büki, G., K. Hadzsiev, and J. Bene, *Copy Number Variations in Neuropsychiatric Disorders.* International Journal of Molecular Sciences, 2023. **24**(18): p. 13671.

226. Endres, D., et al., *Schizophrenia and Hereditary Polyneuropathy: PMP22 Deletion as a Common Pathophysiological Link?* Frontiers in Psychiatry, 2019. **10**.

227. Grayton, H.M., et al., *Copy number variations in neurodevelopmental disorders.* Progress in Neurobiology, 2012. **99**(1): p. 81-91.

228. Shaikh, T.H., *Copy Number Variation Disorders.* Current Genetic Medicine Reports, 2017. **5**(4): p. 183-190.

229. Filteau, M.-J., et al., *Corpus callosum agenesis and psychosis in Andermann syndrome.* Archives of Neurology, 1991. **48**(12): p. 1275-1280.

230. Fichera, M., et al., *Comorbidities in Friedreich ataxia: incidence and manifestations from early to advanced disease stages.* Neurological Sciences, 2022. **43**(12): p. 6831-6838.

231. Ganos, C., et al., *Psychosis complicating Friedreich ataxia.* Movement Disorders Clinical Practice, 2014. **2**(1): p. 84.

232. Carbone, M.G. and F. Della Rocca, *Neuropsychiatric manifestations of fahr's disease, diagnostic and therapeutic challenge: A case report and a literature review.* Clinical Neuropsychiatry, 2022. **19**(2): p. 121.

233. König, P., *Psychopathological alterations in cases of symmetrical basal ganglia sclerosis.* Biol Psychiatry, 1989. **25**(4): p. 459-68.

234. Saleem, S., et al., *Fahr’s syndrome: literature review of current evidence.* Orphanet journal of rare diseases, 2013. **8**: p. 1-9.

235. Curatolo, P., R. Moavero, and P.J. de Vries, *Neurological and neuropsychiatric aspects of tuberous sclerosis complex.* The Lancet Neurology, 2015. **14**(7): p. 733-745.

236. Pokharel, S., et al., *Tuberous sclerosis complex-associated neuropsychiatric disorder (TAND) in a low-resource setting - From seizure to psychosis: A case report.* Ann Med Surg (Lond), 2020. **60**: p. 734-736.

237. Sekijima, Y., *Hereditary Transthyretin Amyloidosis*. 1993: University of Washington, Seattle, Seattle (WA).

238. Uitti, R.J., et al., *Familial Oculoleptomeningeal Amyloidosis: Report of a New Family With Unusual Features.* Archives of Neurology, 1988. **45**(10): p. 1118-1122.

239. Fattal, O., et al., *Review of the Literature on Major Mental Disorders in Adult Patients With Mitochondrial Diseases.* Psychosomatics, 2006. **47**(1): p. 1-7.

240. Schaefer, A.M., et al., *The epidemiology of mitochondrial disorders—past, present and future.* Biochimica et Biophysica Acta (BBA)-Bioenergetics, 2004. **1659**(2-3): p. 115-120.

241. Anglin, R.E., et al., *The psychiatric manifestations of mitochondrial disorders: a case and review of the literature.* The Journal of clinical psychiatry, 2012. **73**(4): p. 15930.

242. Avşar, P.A., E. Akçay, and E. Gürkaş, *Psychotic attack during the clinical course of megalencephalic leukoencephalopathy with subcortical cysts: a case report.* European Child & Adolescent Psychiatry, 2024.

243. Sexmero, M.M. and J. Blanco, *A late-onset Ornitin Transcabamylase deficiency case as an organic psychosis.* European Psychiatry, 2022. **65**(S1): p. S792-S793.

244. Choi, H.Y., *Adult Onset Ornithine Transcarbamylase Deficiency: A Rare Cause of Psychosis.* BJPsych Open, 2024. **10**(S1): p. S274-S275.

245. Enns, G.M., et al., *Postpartum “psychosis” in mild argininosuccinate synthetase deficiency.* Obstetrics & Gynecology, 2005. **105**(5 Part 2): p. 1244-1246.

246. Higashimoto, T., et al., *Maple syrup urine disease decompensation misdiagnosed as a psychotic event.* Molecular Genetics and Metabolism Reports, 2022. **32**: p. 100886.

247. Maciel, P., et al., *Neuroferritinopathy: missense mutation in FTL causing early-onset bilateral pallidal involvement.* Neurology, 2005. **65**(4): p. 603-605.

248. Mir, P., et al., *Adult‐onset generalized dystonia due to a mutation in the neuroferritinopathy gene.* Movement Disorders: Official Journal of the Movement Disorder Society, 2005. **20**(2): p. 243-245.

249. Bâtie, C.D.d.l., et al., *Acute psychosis in propionic acidemia: 2 case reports.* Journal of child neurology, 2014. **29**(2): p. 274-279.

250. Roze, E., et al., *Neuropsychiatric Disturbances in Presumed Late-Onset Cobalamin C Disease.* Archives of Neurology, 2003. **60**(10): p. 1457-1462.

251. Staretz-Chacham, O., et al., *Psychiatric and behavioral manifestations of lysosomal storage disorders.* American Journal of Medical Genetics Part B: Neuropsychiatric Genetics, 2010. **153B**(7): p. 1253-1265.

252. MacQueen, G.M., P.I. Rosebush, and M.F. Mazurek, *Neuropsychiatric aspects of the adult variant of Tay-Sachs disease.* The Journal of neuropsychiatry and clinical neurosciences, 1998. **10**(1): p. 10-19.

253. Hyde, T.M., J.C. Ziegler, and D.R. Weinberger, *Psychiatric disturbances in metachromatic leukodystrophy: insights into the neurobiology of psychosis.* Archives of neurology, 1992. **49**(4): p. 401-406.

254. Kumperscak, H.G., et al., *Adult metachromatic leukodystrophy: a new mutation in the schizophrenia-like phenotype with early neurological signs.* Psychiatric Genetics, 2007. **17**(2): p. 85-91.

255. van Rappard, D.F., et al., *Slowly progressive psychiatric symptoms: think metachromatic leukodystrophy.* Journal of the American Academy of Child & Adolescent Psychiatry, 2018. **57**(2): p. 74-76.

256. Josephs, K., M. Van Gerpen, and J. Van Gerpen, *Adult onset Niemann-Pick disease type C presenting with psychosis.* Journal of Neurology, Neurosurgery & Psychiatry, 2003. **74**(4): p. 528-529.

257. Shulman, L.M., N.J. David, and W.J. Weiner, *Psychosis as the initial manifestation of adult-onset Niemann-Pick disease type C.* Neurology, 1995. **45**(9): p. 1739-1743.

258. Campo, J.V., et al., *Psychosis as a presentation of physical disease in adolescence: a case of Niemann–Pick disease, type C.* Developmental Medicine & Child Neurology, 1998. **40**(2): p. 126-129.

259. Bonnot, O., et al., *Systematic review of psychiatric signs in Niemann-Pick disease type C.* The World Journal of Biological Psychiatry, 2019.

260. Neil, J.F., R.H. Glew, and S.P. Peters, *Familial psychosis and diverse neurologic abnormalities in adult-onset Gaucher's disease.* Archives of Neurology, 1979. **36**(2): p. 95-99.

261. Akram, S., A. Maqsood, and F. Akram, *Psychosis in Gaucher's Disease.* Psychiatric Annals, 2020. **50**(7): p. 317-320.

262. Tullo, M.G., et al., *The spectrum of neurological and sensory abnormalities in Gaucher disease patients: a multidisciplinary study (SENOPRO).* International Journal of Molecular Sciences, 2023. **24**(10): p. 8844.

263. Imbalzano, G., et al., *Neurological symptoms in adults with Gaucher disease: a systematic review.* Journal of Neurology, 2024: p. 1-11.

264. Seidl, U., et al., *Unusual course of α-mannosidosis with symptoms of paranoid-hallucinatory psychosis.* Der Nervenarzt, 2005. **76**: p. 335-338.

265. Gutschalk, A., et al., *Adult α-mannosidosis: Clinical progression in the absence of demyelination.* Neurology, 2004. **63**(9): p. 1744-1746.

266. Bäckman, M.L., E.T. Aronen, and P.R. Santavuori, *New antidepressive and antipsychotic drugs in juvenile neuronal ceroid lipofuscinoses—a pilot study.* European Journal of Paediatric Neurology, 2001. **5**: p. 163-166.

267. Levinson, A.J. and M.F. Mazurek, *Late-onset adrenoleukodystrophy associated with long-standing psychiatric symptoms.* J Clin Psychiatry, 1999. **60**: p. 460-468.

268. Makkar, H., et al., *Adult-Cerebral X-linked Adrenoleukodystrophy: A Mirage of Psychosis, Mania, and Substance Use.* Psychiatric Annals, 2020. **50**(9): p. 417-420.

269. Fraidakis, M.J., *Psychiatric manifestations in cerebrotendinous xanthomatosis.* Translational Psychiatry, 2013. **3**(9): p. e302-e302.

270. Berginer, V.M., et al., *Psychiatric disorders in patients with cerebrotendinous xanthomatosis.* Am J Psychiatry, 1988. **145**(3): p. 354-7.

271. Ellencweig, N., N. Schoenfeld, and Z. Zemishlany, *Acute intermittent porphyria: psychosis as the only clinical manifestation.* Israel Journal of Psychiatry, 2006. **43**(1): p. 52.

272. Duque-Serrano, L., et al., *Psychiatric aspects of acute porphyria: a comprehensive review.* Current psychiatry reports, 2018. **20**: p. 1-7.

273. Kumar, B., *Acute intermittent porphyria presenting solely with psychosis: a case report and discussion.* Psychosomatics, 2012. **53**(5): p. 494-498.

274. Zimbrean, P.C. and M.L. Schilsky, *Psychiatric aspects of Wilson disease: a review.* General hospital psychiatry, 2014. **36**(1): p. 53-62.

275. Litwin, T., et al., *Psychiatric manifestations in Wilson’s disease: possibilities and difficulties for treatment.* Therapeutic advances in psychopharmacology, 2018. **8**(7): p. 199-211.

276. Cox, D.W., *A screening test for Wilson's disease and its application to psychiatric patients.* Can Med Assoc J, 1967. **96**(2): p. 83-6.

277. Brumm, V.L., D. Bilder, and S.E. Waisbren, *Psychiatric symptoms and disorders in phenylketonuria.* Molecular Genetics and Metabolism, 2010. **99**: p. S59-S63.

278. Bilder, D.A., et al., *Psychiatric symptoms in adults with phenylketonuria.* Molecular Genetics and Metabolism, 2013. **108**(3): p. 155-160.

279. Bilder, D.A., et al., *Neuropsychiatric comorbidities in adults with phenylketonuria: A retrospective cohort study.* Molecular Genetics and Metabolism, 2017. **121**(1): p. 1-8.

280. Hector, M. and J.R. Burton, *What are the psychiatric manifestations of vitamin B12 deficiency?* J Am Geriatr Soc, 1988. **36**(12): p. 1105-12.

281. Green, R., et al., *Vitamin B12 deficiency.* Nature reviews Disease primers, 2017. **3**(1): p. 1-20.

282. Parikh, D. and S. Panse, *Pellagra induced psychosis: A rare presentation.* International Journal of Research in Medical Sciences, 2019. **7**(4): p. 1364-1366.

283. Prakash, R., et al., *Rapid resolution of delusional parasitosis in pellagra with niacin augmentation therapy.* General hospital psychiatry, 2008. **30**(6): p. 581-584.

284. Palm, A., et al., *Incidence and mortality of alcohol‐related dementia and Wernicke‐Korsakoff syndrome: A nationwide register study.* International Journal of Geriatric Psychiatry, 2022. **37**(8).

285. Arts, N.J., S.J. Walvoort, and R.P. Kessels, *Korsakoff’s syndrome: a critical review.* Neuropsychiatric disease and treatment, 2017: p. 2875-2890.

286. THOMSON, A.D. and E.J. MARSHALL, *THE NATURAL HISTORY AND PATHOPHYSIOLOGY OF WERNICKE'S ENCEPHALOPATHY AND KORSAKOFF'S PSYCHOSIS.* Alcohol and Alcoholism, 2005. **41**(2): p. 151-158.

287. Harper, C., M. Giles, and R. Finlay-Jones, *Clinical signs in the Wernicke-Korsakoff complex: a retrospective analysis of 131 cases diagnosed at necropsy.* Journal of Neurology, Neurosurgery & Psychiatry, 1986. **49**(4): p. 341-345.

288. Gerridzen, I.J., et al., *Prevalence and severity of behavioural symptoms in patients with Korsakoff syndrome and other alcohol‐related cognitive disorders: A systematic review.* International journal of geriatric psychiatry, 2017. **32**(3): p. 256-273.

289. Kales, S.N., et al., *Paranoid Psychosis after Exposure to Cyanide.* Archives of Environmental Health: An International Journal, 1997. **52**(3): p. 245-246.

290. Rutchik, J. and M.H. Ratner, *Is it Possible for Late-Onset Schizophrenia to Masquerade as Manganese Psychosis?* Journal of Occupational and Environmental Medicine, 2018. **60**(4).

291. Mason, L.H., M.J. Mathews, and D.Y. Han, *Neuropsychiatric symptom assessments in toxic exposure.* Psychiatr Clin North Am, 2013. **36**(2): p. 201-8.

292. WU, H.E., et al., *An Unusual Case of Acute Psychosis With Obsessive-Compulsive Features Following Arsenic Poisoning.* Journal of Psychiatric Practice®, 2017. **23**(5): p. 382-385.

293. Huang, X., et al., *Mercury poisoning: a case of a complex neuropsychiatric illness.* American Journal of Psychiatry, 2014. **171**(12): p. 1253-1256.

294. Fagala, G.E. and C.L. Wigg, *Psychiatric Manifestations of Mercury Poisoning.* Journal of the American Academy of Child & Adolescent Psychiatry, 1992. **31**(2): p. 306-311.

295. Goldbloom, D. and G. Chouinard, *Schizophreniform psychosis associated with chronic industrial toluene exposure: case report.* The journal of clinical psychiatry, 1985. **46**(8): p. 350-351.

296. Stein, Y., et al., *Exposure and susceptibility: Schizophrenia in a young man following prolonged high exposures to organic solvents.* NeuroToxicology, 2010. **31**(5): p. 603-607.

297. Kohlmeier, R.E., *Chronic Lead Poisoning: Induced Psychosis in an Adult?* The American Journal of Forensic Medicine and Pathology, 2002. **23**(1): p. 101.

298. Vorvolakos, T., S. Arseniou, and M. Samakouri, *There is no safe threshold for lead exposure: Α literature review.* Psychiatriki, 2016. **27**(3): p. 204-214.

299. Magoub, N. and M. Tahir, *Is it medication-induced psychosis or prodromal psychosis unmasked by medication?* The Journal of Neuropsychiatry and Clinical Neurosciences, 2011. **23**(3): p. E13-E14.

300. Reinhardt, M.M. and C.I. Cohen, *Late-Life Psychosis: Diagnosis and Treatment.* Current Psychiatry Reports, 2015. **17**(2): p. 1.

301. Wood, K.A., et al., *Drug-Induced Psychosis and Depression in the Elderly.* Psychiatric Clinics of North America, 1988. **11**(1): p. 167-193.

302. Barateau, L., et al., *Narcolepsy.* Journal of Sleep Research, 2022. **31**(4): p. e13631.

303. Hanin, C., et al., *Narcolepsy and psychosis: A systematic review.* Acta Psychiatrica Scandinavica, 2021. **144**(1): p. 28-41.

304. Fortuyn, H.A.D., et al., *Psychotic symptoms in narcolepsy: phenomenology and a comparison with schizophrenia.* General Hospital Psychiatry, 2009. **31**(2): p. 146-154.

305. Arnulf, I., et al., *Kleine–Levin syndrome: a systematic review of 186 cases in the literature.* Brain, 2005. **128**(12): p. 2763-2776.

306. Luks, A.M., E.R. Swenson, and P. Bärtsch, *Acute high-altitude sickness.* European Respiratory Review, 2017. **26**(143).

307. Hüfner, K., et al., *Isolated high altitude psychosis, delirium at high altitude, and high altitude cerebral edema: are these diagnoses valid?* Frontiers in Psychiatry, 2023. **14**: p. 1221047.

**Appendix 3: Type of Evidence used in Organic Psychoses Scoping Review**

|  | **Type of Evidence** |
| --- | --- |
| Epilepsy | Systematic review |
| Multiple Sclerosis | Systematic review (of case reports) |
| Neuromyelitis optica | Case report |
| ADEM | Systematic review (of cohorts) |
| Autoimmune encephalitis | Cohort |
| Systemic Lupus Erythematosus | Cohort |
| Sjogren's syndrome | Case series |
| Anti-phospholipid syndrome | Systematic review (of case reports) |
| Neurosarcoidosis | Case report |
| Acute infectious encephalitis | Systematic review (TB), case series (mycoplasma), cohort (bacterial infections – sequelae) |
| Prion (CJD) disease | Cohort |
| Neuroborreliosis | Narrative review including cases |
| Syphilis | Systematic review (of cases) |
| HIV | Systematic review (of cases) |
| Subacute sclerosing panencephalitis | Systematic review (of cases and case series) |
| Normal pressure hydrocephalus | Cohort |
| Acute stroke | Systematic review (of cohort) |
| Chronic subdural haematoma | Case report |
| Vasculitis | Case report |
| AV malformation | Case report |
| Migraine | Case report |
| Cerebral neoplasm | Systematic review (of case reports) |
| Traumatic Brain Injury | Systematic review (of case reports) |
| Malformation of cortical development | Cohort |
| Porencephaly | Case report |
| Delirium | Systematic review |
| Huntington's Disease | Systematic review |
| Alzheimer’s Disease | Systematic review |
| Parkinson's Disease | Systematic review |
| Lewy Body Disease | Cohort |
| Vascular dementia | Cohort |
| ALS/Frontotemporal dementia | Systematic review |
| Hyperthyroidism | Cohort |
| Hypothyroidism | Narrative review including case series and case reports |
| EAAT (autoimmune thyroid encephalopathy) | Systematic review |
| Hypoparathyroidism | Case series |
| Hyperparathyroidism | Case report |
| Cushing's Syndrome | Systematic review |
| Adrenal insufficiency | Case report |
| Phaeochromocytoma | Case report |
| Down Syndrome | Cohort study |
| Fragile X Syndrome | Case report |
| Klinefelter Syndrome | Case control |
| Turner Syndrome | Case report |
| Retinitis Pigmentosa/Usher Syndrome | Case report |
| 22q11.2 deletion (Velocardiofacial Syndrome) | Case control |
| Prader Willi Syndrome | Systematic review |
| Williams-Beuren Syndrome | Case series |
| Other copy number variation associations | Case control study |
| Andermann Syndrome | Case control study |
| Friedreich ataxia | Cohort study |
| Fahr's disease | Case report |
| Tuberous Sclerosis | Case report |
| Amyloidosis | Case report |
| Mitochondrial disorders | Systematic review (of case reports) |
| Megalencephalic leukoencephalopathy | Case report |
| Ornithine transcarbamylase deficiency | Case report |
| Citrullinaemia type I (Arginosuccinate synthetase deficiency) | Case report |
| Maple syrup urine disease | Case report |
| Neuroferritinopathy | Case report |
| Propionic acidemia | Case report |
| Cobalamin C disease | Case report |
| Tay-Sachs disease | Narrative review with case reports |
| Metachromatic leukodystrophy | Narrative review with case reports |
| Niemann-Pick Disease Type C | Systematic review |
| Gaucher disease | Case series (one family) |
| α-Mannosidosis | Case series |
| Neuronal ceroid lipofuscinoses | Case series |
| X-Adrenoleukodystrophy late onset | Case report |
| CerebroTendinous Xanthomatosis | Case series |
| Porphyrias | Case report |
| Wilson’s disease | Systematic review |
| Phenylketonuria | Cohort |
| B12/Cobalamin deficiency | Narrative review (includes case series and cohort studies) |
| Pellagra/Niacin/B3 deficiency | Case report |
| Wernicke-Korsakoff/Thiamine/B1 deficiency | Systematic review |
| Cyanide toxicity | Case report |
| Manganese toxicity | Case report |
| Arsenic toxicity | Case report |
| Mercury toxicity | Case report |
| Toluene and other solvents toxicity | Case report |
| Lead toxicity | Case report |
| Narcolepsy | Systematic review |
| Klein Levin Syndrome | Systematic review |
| High altitude cerebral oedema | Case report |

**Appendix 4. Included and Excluded Guidelines**

| **Guideline** | **Year** | **Inclusion/Exclusion** |
| --- | --- | --- |
| **International/Country not specified** |  |  |
| International consensus on late onset schizophrenia [310] | 2000 | Excluded: Insufficient information - recommends physical examination for all and neuroimaging where available |
| Expert consensus optimising pharmacotherapy, Kane et al. [311] | 2003 | Excluded: Insufficient information - noted risks of obesity, diabetes, HIV and syphilis |
| International early psychosis association writing group [312] | 2005 | Excluded: Insufficient information – recommends ruling out organic causes |
| Guidelines for early course of schizophrenia, Keshavan et al. [313] | 2006 | Included |
| International college of neuropsychopharmacology (CINP) [314, 315] | 2011 | Excluded: Insufficient information – recommends excluding somatic causes of psychosis |
| “Meta-Guidelines”, Stahl et al. [316] | 2013 | Included |
| The Office of the United Nations High Commissioner for Refugees (UNHCR) [317] | 2017 | Excluded: Insufficient information |
| World Federation of Societies of Biological Psychiatry (WFSBP) [318-324] | 2005/06/12/13/15/17 | 2017 Included |
| Consensus on treatment resistant schizophrenia [325] | 2019 | Excluded: Insufficient information |
| First Episode Psychosis Medical Workup, Skikic and Arriola [326] | 2020 | Included |
| Treatment Response and Resistance in Psychosis working group (TRRIP) [327-329] | 2017/20/23 | Excluded: Insufficient information |
|  |  |  |
| **Africa** |  |  |
| The South African Society of Psychiatrists [330] | 2007 | Included |
|  |  |  |
| **Asia** |  |  |
| Royal College of Psychiatrists of Thailand [331] | 2000 | Excluded: Insufficient information |
| Singapore Ministry of Health [332] | 2011 | Excluded: Insufficient information – recommends to exclude physical causes of psychosis |
| Asia Pacific Region consensus [333] | 2016 | Excluded: Insufficient information – recommends regular metabolic screening |
| Indian Psychiatric Society [334, 335] | 2017/19 | 2019 Included |
| Korean College of Neuropsychopharmacology and the Korean Society for Schizophrenia Research [336, 337] | 2019/20 | Excluded: Referenced WFSBP [321] assessment schedule with no changes. |
| Japanese Society of Neuropsychopharmacology [338, 339] | 2021 | Excluded: Insufficient information – recommends metabolic screening/physical health monitoring |
| Ministry of Health, Malaysia [340] | 2021 | Included |
| Psychiatry Society of Chinese Medical Association [341] | 2023 | Excluded: Guideline currently not published |
|  |  |  |
| **Australia and New Zealand** |  |  |
| Australian Clinical Guidelines for Early Psychosis [342, 343] | 2011/16 | 2016 Included |
| Royal Australian and New Zealand College of Psychiatrists (RANZCP) [344, 345] | 2005/16 | 2016 Included |
|  |  |  |
| **Europe** |  |  |
| Sistema Nazionale Linee Guida (SNLG; Italian National Guidelines System) [346] | 2008 | Excluded: Insufficient information – recommends CT/MRI brain when clinically indicated |
| National Steering Group Multidisciplinary Guideline Development in Mental Health Care, Holland [347, 348] | 2005/12 | Excluded: Insufficient information – directs physical exam and investigations recommendations to Comprehensive Care of Schizophrenia, Textbook of Clinical Management |
| Spanish Societies of Psychiatry and Biological Psychiatry [349] | 2012 | Included |
| French Association for Biological Psychiatry and Neuropsychopharmacology (AFPBN) [350, 351] | 2006/13 | 2013 Included |
| Scottish Intercollegiate Guidelines Network [352] | 2013 | Included |
| National Institute for Health and Care Excellence (NICE) [353] | 2014 | Included |
| The Danish Health and Medicines Authority [354] | 2015 | Excluded: insufficient information |
| The Estonian Psychiatrists Association [355, 356] | 2016 | Excluded: insufficient information |
| Greek Ministry of Health [357] | 2018 | Included |
| National Board of Health and Welfare, Sweden [358] | 2018 | Excluded: insufficient information – Recommended regular checks of metabolic risk factors with blood tests and physical examination |
| German Association for Psychiatry, Psychotherapy and Psychosomatics, DGPPN [359] | 2019 | Included |
| British Association of Psychopharmacology [360-362] | 2011/16/20 | 2020 Included |
| Finnish Medical Association and the Finnish Psychiatry Association [363] | 2020 | Included |
| European Psychiatric Association [364-366] | 2015/21/21 | Excluded: insufficient information |
| Polish Psychiatric Association [367, 368] | 2019/22 | 2019 Included, insufficient information in 2022 guideline |
|  |  |  |
| **America** |  |  |
| Mount Sinai Conference Consensus [369] | 2004 | Included |
| Texas Medication Algorithm Project (TMAP) [370] | 2007 | Excluded: insufficient information |
| The Schizophrenia Patient Outcomes Research Team (PORT) [371, 372] | 2004/10 | Excluded: insufficient information – recommended routine metabolic monitoring |
| Veterans Affairs Quality Enhancement Research Initiative (QUERI) [373] | 2011 | Excluded: insufficient information – recommends routine metabolic monitoring with HbA1c, BMI, lipids measurement |
| American Academy of Child and Adolescent Psychiatry (AACAP) [374, 375] | 2000/13 | 2013 Included |
| Canadian Psychiatric Association [376-380] | 2005/2017 | 2017 Included – note over multiple publications |
| American association of community psychiatrists [381] | 2017 | Excluded: insufficient information |
| American Psychiatric Association (APA) [382-384] | 2004/09/20 | 2020 Included |
| Florida Best Practice expert panel [385] | 2020 | Excluded: insufficient information – recommended physical health assessment |
| Columbia University [386] | 2023 | Included |
|  |  |  |
| The University of Chile High-risk Intervention Program [387, 388] | 2011/19 | 2019 Included |
| Academia Nacional de Medicina de México [389] | 2021 | Excluded: insufficient information |

**References for Included/Excluded Guidelines**

1. Tricco, A.C., et al., *PRISMA Extension for Scoping Reviews (PRISMA-ScR): Checklist and Explanation.* Ann Intern Med, 2018. **169**(7): p. 467-473.

2. Page, M.J., et al., *PRISMA 2020 explanation and elaboration: updated guidance and exemplars for reporting systematic reviews.* BMJ, 2021. **372**: p. n160.

3. Fiest, K.M., et al., *Prevalence and incidence of epilepsy: a systematic review and meta-analysis of international studies.* Neurology, 2017. **88**(3): p. 296-303.

4. Clancy, M.J., et al., *The prevalence of psychosis in epilepsy; a systematic review and meta-analysis.* BMC Psychiatry, 2014. **14**: p. 75.

5. Wotton, C.J. and M.J. Goldacre, *Coexistence of schizophrenia and epilepsy: record-linkage studies.* Epilepsia, 2012. **53**(4): p. e71-4.

6. Agrawal, N. and M. Mula, *Treatment of psychoses in patients with epilepsy: an update.* Ther Adv Psychopharmacol, 2019. **9**: p. 2045125319862968.

7. Mula, M., *Epilepsy-induced behavioral changes during the ictal phase.* Epilepsy Behav, 2014. **30**: p. 14-6.

8. Perven, G. and N.K. So, *Epileptic auras: phenomenology and neurophysiology.* Epileptic Disorders, 2015. **17**(4): p. 349-362.

9. Elliott, B., E. Joyce, and S. Shorvon, *Delusions, illusions and hallucinations in epilepsy: 2. Complex phenomena and psychosis.* Epilepsy Research, 2009. **85**(2): p. 172-186.

10. Forouhari, A., et al., *Multiple sclerosis epidemiology in Asia and Oceania; A systematic review and meta-analysis.* Multiple Sclerosis and Related Disorders, 2021. **54**: p. 103119.

11. Pugliatti, M., S. Sotgiu, and G. Rosati, *The worldwide prevalence of multiple sclerosis.* Clinical neurology and neurosurgery, 2002. **104**(3): p. 182-191.

12. Marrie, R.A., et al., *The incidence and prevalence of psychiatric disorders in multiple sclerosis: a systematic review.* Mult Scler, 2015. **21**(3): p. 305-17.

13. Camara-Lemarroy, C.R., et al., *The varieties of psychosis in multiple sclerosis: A systematic review of cases.* Mult Scler Relat Disord, 2017. **12**: p. 9-14.

14. Sabe, M. and O. Sentissi, *Psychotic symptoms prior or concomitant to diagnosis of multiple sclerosis: a systematic review of case reports and case series.* Int J Psychiatry Clin Pract, 2022. **26**(3): p. 287-293.

15. Papp, V., et al., *Worldwide incidence and prevalence of neuromyelitis optica: a systematic review.* Neurology, 2021. **96**(2): p. 59-77.

16. Alam, A., et al., *Neuromyelitis optica presenting with psychiatric symptoms and catatonia: a case report.* General Hospital Psychiatry, 2015. **37**(3): p. 274.e1-274.e2.

17. Moore, P., et al., *Cognitive and psychiatric comorbidities in neuromyelitis optica.* Journal of the Neurological Sciences, 2016. **360**: p. 4-9.

18. Hor, J.Y. and K. Fujihara, *Epidemiology of myelin oligodendrocyte glycoprotein antibody-associated disease: a review of prevalence and incidence worldwide.* Frontiers in Neurology, 2023. **14**: p. 1260358.

19. Zhou, J., et al., *An unusual case of anti-MOG CNS demyelination with concomitant mild anti-NMDAR encephalitis.* J Neuroimmunol, 2018. **320**: p. 107-110.

20. von Zedtwitz, K., et al., *Anti-MOG autoantibody-associated schizophreniform psychosis.* Acta neuropsychiatrica, 2022. **34**(1): p. 47-54.

21. Zahid, A., et al., *First Episode Psychosis and Status Epilepticus as Presenting Symptom of FLAIR-hyperintense Lesions in Anti-MOG-associated Encephalitis with Seizures (FLAMES).* Neurology, 2024. **103**(7_Supplement_1): p. S47-S47.

22. Cheng, X., et al., *Anti-Myelin Oligodendrocyte Glycoprotein Antibody-Associated Meningitis with Psychotic Symptoms.* Authorea Preprints, 2024.

23. Li, K., et al., *Clinical presentation and outcomes of acute disseminated Encephalomyelitis in adults worldwide: systematic review and meta-analysis.* Frontiers in Immunology, 2022. **13**: p. 870867.

24. Nasr, J.T., M.R. Andriola, and P.K. Coyle, *Adem: literature review and case report of acute psychosis presentation.* Pediatric Neurology, 2000. **22**(1): p. 8-18.

25. Endres, D., et al., *Autoimmune encephalitis as a differential diagnosis of schizophreniform psychosis: clinical symptomatology, pathophysiology, diagnostic approach, and therapeutic considerations.* European archives of psychiatry and clinical neuroscience, 2020. **270**: p. 803-818.

26. Dalmau, J., et al., *An update on anti-NMDA receptor encephalitis for neurologists and psychiatrists: mechanisms and models.* The Lancet Neurology, 2019. **18**(11): p. 1045-1057.

27. Kayser, M.S., et al., *Frequency and characteristics of isolated psychiatric episodes in anti–N-methyl-d-aspartate receptor encephalitis.* JAMA Neurol, 2013. **70**(9): p. 1133-9.

28. Dalmau, J. and F. Graus, *Diagnostic criteria for autoimmune encephalitis: utility and pitfalls for antibody-negative disease.* The Lancet Neurology, 2023. **22**(6): p. 529-540.

29. Steiner, J., et al., *Autoimmune encephalitis with psychosis: Warning signs, step-by-step diagnostics and treatment.* The World Journal of Biological Psychiatry, 2020. **21**(4): p. 241-254.

30. Graus, F., et al., *A clinical approach to diagnosis of autoimmune encephalitis.* Lancet Neurol, 2016. **15**(4): p. 391-404.

31. Rees, F., et al., *The worldwide incidence and prevalence of systemic lupus erythematosus: a systematic review of epidemiological studies.* Rheumatology, 2017. **56**(11): p. 1945-1961.

32. Appenzeller, S., F. Cendes, and L.T.L. Costallat, *Acute psychosis in systemic lupus erythematosus.* Rheumatology international, 2008. **28**: p. 237-243.

33. Pego-Reigosa, J.M. and D.A. Isenberg, *Psychosis due to systemic lupus erythematosus: characteristics and long-term outcome of this rare manifestation of the disease.* Rheumatology, 2008. **47**(10): p. 1498-1502.

34. Abrol, E., et al., *Psychosis in systemic lupus erythematosus (SLE): 40-year experience of a specialist centre.* Rheumatology, 2021. **60**(12): p. 5620-5629.

35. Hanly, J.G., et al., *Psychosis in systemic lupus erythematosus: results from an international inception cohort study.* Arthritis & rheumatology, 2019. **71**(2): p. 281-289.

36. Qin, B., et al., *Epidemiology of primary Sjögren's syndrome: a systematic review and meta-analysis.* Annals of the rheumatic diseases, 2015. **74**(11): p. 1983-1989.

37. Hammett, E.K., et al., *Adolescent Sjogren’s syndrome presenting as psychosis: a case series.* Pediatric Rheumatology, 2020. **18**: p. 1-8.

38. Pt, S., et al., *Primary Sjogren's syndrome presenting as acute psychosis with catatonic symptoms.* Indian Journal of Psychiatry, 2022. **64**(Suppl 3).

39. Lin, C.-E., *One patient with Sjogren’s syndrome presenting schizophrenia-like symptoms.* Neuropsychiatric Disease and Treatment, 2016: p. 661-663.

40. Ruiz-Irastorza, G., et al., *Antiphospholipid syndrome.* The Lancet, 2010. **376**(9751): p. 1498-1509.

41. Hallab, A., et al., *Association of psychosis with antiphospholipid antibody syndrome: A systematic review of clinical studies.* General Hospital Psychiatry, 2018. **50**: p. 137-147.

42. Rossides, M., et al., *Sarcoidosis: Epidemiology and clinical insights.* Journal of internal medicine, 2023. **293**(6): p. 668-680.

43. Van Hoye, G., et al., *Case report: Psychosis with catatonia in an adult man: a presentation of neurosarcoidosis.* Frontiers in Psychiatry, 2024. **15**: p. 1276744.

44. Blanco, L.S., et al., *Psychotic disorder of organic etiology, in the context of sarcoidosis. A case report.* European Psychiatry, 2017. **41**(S1): p. s836-s837.

45. Bona, J.R., et al., *Neurosarcoidosis as a cause of refractory psychosis: a complicated case report.* American Journal of Psychiatry, 1998. **155**(8): p. 1106-1108.

46. Celebi, A., et al., *A case of isolated neurosarcoidosis associated with psychosis.* Neurosciences Journal, 2013. **18**(1): p. 70-73.

47. Duerlund, L.S., H. Nielsen, and J. Bodilsen, *Current epidemiology of infectious encephalitis: a narrative review.* Clinical Microbiology and Infection, 2024.

48. Zunt, J.R., et al., *Global, regional, and national burden of meningitis, 1990–2016: a systematic analysis for the Global Burden of Disease Study 2016.* The Lancet Neurology, 2018. **17**(12): p. 1061-1082.

49. Oommen, K.J., P.C. Johnson, and C.G. Ray, *Herpes simplex type 2 virus encephalitis presenting as psychosis.* The American Journal of Medicine, 1982. **73**(3): p. 445-448.

50. Torrey, E.F., *Functional psychoses and viral encephalitis.* Integrative Psychiatry, 1986. **4**(4): p. 224-230.

51. Ariza-Varón, M., et al., *Psychosis associated with suspected SARS-CoV-2 encephalitis with response to steroids: a case report.* Infectious Diseases, 2022. **54**(2): p. 152-156.

52. Monnet, F.P., *Behavioural disturbances following Japanese B encephalitis.* European Psychiatry, 2003. **18**(6): p. 269-273.

53. Hsu, T.-W., et al., *Risk of Major Mental disorder after severe bacterial Infections in children and adolescents: a Nationwide Longitudinal Study.* Neuropsychobiology, 2022. **81**(6): p. 539-549.

54. Banerjee, B. and K. Petersen, *Psychosis Following Mycoplasma Pneumonia.* Military Medicine, 2009. **174**(9): p. 1001-1004.

55. Cohen, M. and A. Jay, *Psychosis in a Case of Mycoplasma Pneumonia Encephalopathy.* Jefferson Journal of Psychiatry, 1986. **4**(2): p. 10.

56. Sterner, G. and G. Biberfeld, *Central nervous system complications of Mycoplasma pneumoniae infection.* Scandinavian Journal of Infectious Diseases, 1969. **1**(3): p. 203-208.

57. Rahim, M.J. and W.S. Ghazali, *Psychosis secondary to tuberculosis meningitis.* BMJ Case Rep, 2016. **2016**.

58. Gunawardena, S.M., E. McMahon, and A.M. Doherty, *A systematic review of the prevalence of psychosis in people with tuberculosis.* European Psychiatry, 2024. **67**(S1): p. S370-S370.

59. Sa'adah, M., et al., *Cryptococcal meningitis and confusional psychosis. A case report and literature review.* Tropical and geographical medicine, 1995. **47**(5): p. 224-226.

60. Michielsen, P. and M. Arts, *Cryptococcal Meningitis in Acute Onset Psychosis: A Case-Study.* European Psychiatry, 2016. **33**(S1): p. S532-S532.

61. ElemamaliI, A., S. Talyb, and A. Awad, *Neurocysticercosis presenting with psychosis.* New Horizons in Clinical Case Reports, 2017. **1**: p. 23.

62. Carpio, A., et al., *Parasitic diseases of the central nervous system: lessons for clinicians and policy makers.* Expert Rev Neurother, 2016. **16**(4): p. 401-14.

63. Chen, C. and X.-P. Dong, *Epidemiological characteristics of human prion diseases.* Infectious diseases of poverty, 2016. **5**: p. 1-10.

64. Thompson, A., et al., *Behavioral and psychiatric symptoms in prion disease.* American Journal of Psychiatry, 2014. **171**(3): p. 265-274.

65. Appleby, B.S., K.K. Appleby, and P.V. Rabins, *Does the presentation of Creutzfeldt-Jakob disease vary by age or presumed etiology? A meta-analysis of the past 10 years.* J Neuropsychiatry Clin Neurosci, 2007. **19**(4): p. 428-35.

66. Koedel, U., V. Fingerle, and H.-W. Pfister, *Lyme neuroborreliosis—epidemiology, diagnosis and management.* Nature Reviews Neurology, 2015. **11**(8): p. 446-456.

67. Mattingley, D.W. and M.M. Koola, *Association of Lyme Disease and Schizoaffective Disorder, Bipolar Type: Is it Inflammation Mediated?* Indian J Psychol Med, 2015. **37**(2): p. 243-6.

68. Bar, K.-J., et al., *Painful hallucinations and somatic delusions in a patient with the possible diagnosis of neuroborreliosis.* The Clinical journal of pain, 2005. **21**(4): p. 362-363.

69. Pasareanu, A.R., Å. Mygland, and Ø. Kristensen, *A woman in her 50s with manic psychosis.* Tidsskrift for Den norske legeforening, 2012.

70. Hess, A., et al., *Borrelia burgdorferi central nervous system infection presenting as an organic schizophrenialike disorder.* Biological psychiatry, 1999. **45**(6): p. 795.

71. Pfister, H.-W., et al., *Catatonic syndrome in acute severe encephalitis due to Borrelia burgdorferi infection.* Neurology, 1993. **43**(2): p. 433-433.

72. Fallon, B.A. and J.A. Nields, *Lyme disease: a neuropsychiatric illness.* American Journal of Psychiatry, 1994. **151**(11): p. 1571-1583.

73. King, J., et al., *HIV, viral hepatitis and sexually transmissible infections in Australia Annual Surveilance report 2022. The Kirby Institute, UNSW Sydney, Sydney, Australia; 2022*.

74. Nutile, L.M., *Neurosyphilis with psychosis as the primary presentation.* American Journal of Psychiatry Residents' Journal, 2021.

75. Friedrich, F., et al., *Psychosis in neurosyphilis-clinical aspects and implications.* Psychopathology, 2013. **47**(1): p. 3-9.

76. Jarocki, A., et al., *Neurosyphilis-Induced Psychosis in Europe: A Systematic Review of Case Reports.* Pathogens, 2024. **13**(11): p. 959.

77. Nebhinani, N. and S.K. Mattoo, *Psychotic disorders with HIV infection: A review.* German J Psychiatry, 2013. **16**(1): p. 43-48.

78. Sewell, D.D., et al., *HIV-associated psychosis: a study of 20 cases. San Diego HIV Neurobehavioral Research Center Group.* Am J Psychiatry, 1994. **151**(2): p. 237-42.

79. Vogel-Scibilia, S.E., B.H. Mulsant, and M.S. Keshavan, *HIV infection presenting as psychosis: a critique.* Acta Psychiatrica Scandinavica, 1988. **78**(5): p. 652-656.

80. De Ronchi, D., et al., *Psychopathology of first-episode psychosis in HIV-positive persons in comparison to first-episode schizophrenia: a neglected issue.* AIDS care, 2006. **18**(8): p. 872-878.

81. Van Rensburg, B.J. and C. Bracken, *Acute psychiatric in-patients tested for HIV status: A clinical profile.* South African Journal of Psychiatry, 2007. **10**(1).

82. Garg, R.K., et al., *The spectrum of psychiatric manifestations in subacute sclerosing panencephalitis: A systematic review of published case reports and case series.* CNS Spectrums, 2024. **29**(2): p. 87-95.

83. Gutierrez, J., R.S. Issacson, and B.S. Koppel, *Subacute sclerosing panencephalitis: an update.* Developmental Medicine & Child Neurology, 2010. **52**(10): p. 901-907.

84. Prashanth, L., et al., *Subacute sclerosing panencephalitis (SSPE): an insight into the diagnostic errors from a tertiary care university hospital.* Journal of child neurology, 2007. **22**(6): p. 683-688.

85. Alonso, B.L., *Atypical psychosis in a patient with intracranial hypertension: clinical case and review.* European Psychiatry, 2024. **67**(S1): p. S488-S489.

86. Vakis, A.F., et al., *Psychosis-like syndrome associated with intermittent intracranial hypertension caused by a large arachnoid cyst of the left temporal lobe.* British journal of neurosurgery, 2006. **20**(3): p. 156-159.

87. Duggal, H.S., *Idiopathic intracranial hypertension presenting with psychiatric symptoms.* The Journal of Neuropsychiatry and Clinical Neurosciences, 2005. **17**(3): p. 426-427.

88. de Oliveira, M.F., et al., *Psychiatric symptoms are frequent in idiopathic intracranial hypertension patients.* Neurosurgical Review, 2021. **44**(2): p. 1183-1189.

89. Korsbæk, J.J., et al., *Psychiatric comorbidities in patients with idiopathic intracranial hypertension: a prospective cohort study.* Neurology, 2022. **99**(2): p. e199-e208.

90. Zaccaria, V., et al., *A systematic review on the epidemiology of normal pressure hydrocephalus.* Acta Neurologica Scandinavica, 2020. **141**(2): p. 101-114.

91. Vanhala, V., et al., *Prevalence of Schizophrenia in Idiopathic Normal Pressure Hydrocephalus.* Neurosurgery, 2019. **84**(4).

92. Saini, V., L. Guada, and D.R. Yavagal, *Global epidemiology of stroke and access to acute ischemic stroke interventions.* Neurology, 2021. **97**(20_Supplement_2): p. S6-S16.

93. Stangeland, H., V. Orgeta, and V. Bell, *Poststroke psychosis: a systematic review.* Journal of Neurology, Neurosurgery & Psychiatry, 2018. **89**(8): p. 879-885.

94. Almeida, O.P. and J. Xiao, *Mortality associated with incident mental health disorders after stroke.* Australian & New Zealand Journal of Psychiatry, 2007. **41**(3): p. 274-281.

95. Feghali, J., W. Yang, and J. Huang, *Updates in chronic subdural hematoma: epidemiology, etiology, pathogenesis, treatment, and outcome.* World neurosurgery, 2020. **141**: p. 339-345.

96. Kar, S.K., et al., *Psychiatric manifestation of chronic subdural hematoma: the unfolding of mystery in a homeless patient.* Indian Journal of Psychological Medicine, 2015. **37**(2): p. 239-242.

97. Alp, A., et al., *Recurrent Psychotic Depression Associated with Chronic Subdural Haematoma: A Case Report.* Archives of Neuropsychiatry, 2024. **61**(2): p. 180.

98. Singh, T.B., et al., *Chronic subdural haematoma presenting as late onset psychosis.* ASEAN J Psychiatry, 2013. **14**: p. 175-8.

99. Watts, R.A., et al., *Global epidemiology of vasculitis.* Nature reviews rheumatology, 2022. **18**(1): p. 22-34.

100. Fontes, H., et al., *Central nervous system vasculitis presenting as acute psychotic disorder.* European Psychiatry, 2011. **26**(S2): p. 745-745.

101. Gasparinho, R., et al., *Psychosis as the First Manifestation of Granulomatosis with Polyangiitis-A Case Report.* Psychiatria Danubina, 2022. **34**(2): p. 315-317.

102. Latvala, H.M., S.K. Reitan, and A.E. Vaaler, *Giant Cell Arteritis Presenting with Mania, Psychosis, and Cognitive Dysfunction: A Case Report.* Case Reports in Psychiatry, 2023. **2023**(1): p. 7989712.

103. Patel, P., et al., *Neuro-Behçet disease presenting with acute psychosis in an adolescent.* Journal of child neurology, 2014. **29**(9): p. NP86-NP91.

104. Nelson, E.A., et al., *Auditory hallucinations associated with an arteriovenous malformation of the brain.* The Journal of Neuropsychiatry and Clinical Neurosciences, 2022. **34**(1): p. 89-92.

105. Shah, C.C., et al., *Clinically Isolated Syndrome and Frontal Lobe Arteriovenous Malformation Presenting With Behavior Issues.* Journal of Medical Cases, 2022. **14**(1): p. 1.

106. Aleem, A. and M.A. Knesevich, *Schizophrenia-like psychosis associated with vein of Galen malformation: a case report.* The Canadian Journal of Psychiatry, 1987. **32**(3): p. 226-227.

107. Hanine, I., et al., *Seizures, Psychosis, and Cerebral Vascular Malformation: A Rare Chain of Events.* Case Reports in Psychiatry, 2024. **2024**(1): p. 8656715.

108. Villa, N.A.E. and E.D. Espiridion, *Neuropsychiatric Manifestations of Arteriovenous Malformation: A Case of Acute Mania.* Cureus, 2024. **16**(4): p. e58297.

109. Steiner, T.J. and L.J. Stovner, *Global epidemiology of migraine and its implications for public health and health policy.* Nature Reviews Neurology, 2023. **19**(2): p. 109-117.

110. LaBianca, S., et al., *Familial Hemiplegic Migraine and Recurrent Episodes of Psychosis: A Case Report.* Headache, 2015. **55**(7): p. 1004-7.

111. Fuller, G.N., et al., *Migraine madness: recurrent psychosis after migraine.* Journal of Neurology, Neurosurgery & Psychiatry, 1993. **56**(4): p. 416-418.

112. Feely, M., et al., *Episodes of acute confusion or psychosis in familial hemiplegic migraine.* Acta Neurologica Scandinavica, 1982. **65**(4): p. 369-375.

113. Miller, E.E., et al., *Auditory hallucinations associated with migraine: Case series and literature review.* Cephalalgia, 2015. **35**(10): p. 923-930.

114. Spranger, M., et al., *Familial hemiplegic migraine with cerebellar ataxia and paroxysmal psychosis.* European neurology, 1999. **41**(3): p. 150-152.

115. Barros, J., et al., *Psychotic aura symptoms in familial hemiplegic migraine type 2 (ATP1A2).* J Headache Pain, 2012. **13**(7): p. 581-5.

116. Gurney, J.G. and N. Kadan-Lottick, *Brain and other central nervous system tumors: rates, trends, and epidemiology.* Current opinion in oncology, 2001. **13**(3): p. 160-166.

117. Madhusoodanan, S., et al., *Psychiatric aspects of brain tumors: a review.* World journal of psychiatry, 2015. **5**(3): p. 273.

118. Ghandour, F., et al., *Presenting psychiatric and neurological symptoms and signs of brain tumors before diagnosis: a systematic review.* Brain sciences, 2021. **11**(3): p. 301.

119. Corrigan, J.D., A.W. Selassie, and J.A.L. Orman, *The epidemiology of traumatic brain injury.* The Journal of head trauma rehabilitation, 2010. **25**(2): p. 72-80.

120. Fujii, D. and I. Ahmed, *Characteristics of psychotic disorder due to traumatic brain injury: an analysis of case studies in the literature.* J Neuropsychiatry Clin Neurosci, 2002. **14**(2): p. 130-40.

121. Sachdev, P., J.S. Smith, and S. Cathcart, *Schizophrenia-like psychosis following traumatic brain injury: a chart-based descriptive and case-control study.* Psychol Med, 2001. **31**(2): p. 231-9.

122. Kostenniemi, U.J., et al., *Psychiatric disabilities and other long-term consequences of childhood bacterial meningitis.* The Pediatric Infectious Disease Journal, 2021. **40**(1): p. 26-31.

123. Rantakallio, P., et al., *Association between central nervous system infections during childhood and adult onset schizophrenia and other psychoses: a 28-year follow-up.* International journal of epidemiology, 1997. **26**(4): p. 837-843.

124. Dalman, C., et al., *Infections in the CNS during childhood and the risk of subsequent psychotic illness: a cohort study of more than one million Swedish subjects.* American Journal of Psychiatry, 2008. **165**(1): p. 59-65.

125. Leask, S.J., D.J. Done, and T.J. Crow, *Adult psychosis, common childhood infections and neurological soft signs in a national birth cohort.* The British Journal of Psychiatry, 2002. **181**(5): p. 387-392.

126. Khandaker, G.M., et al., *Childhood infection and adult schizophrenia: a meta-analysis of population-based studies.* Schizophrenia research, 2012. **139**(1-3): p. 161-168.

127. Pang, T., R. Atefy, and V. Sheen, *Malformations of cortical development.* The neurologist, 2008. **14**(3): p. 181-191.

128. Ho, C.S.H., et al., *Prevalence of neuropsychiatric symptoms associated with malformations of cortical development.* Epilepsy & Behavior, 2019. **92**: p. 306-310.

129. Kawaguchi, A., et al., *A case of schizophrenia accompanied by lissencephaly.* The Journal of Neuropsychiatry and Clinical Neurosciences, 2013. **25**(4): p. E17-E18.

130. Guerra, M., et al., *Porencephaly in the adult age.* Ann Clin Case Rep, 2021. **6**: p. 1955.

131. Douzenis, A., et al., *Porencephaly and psychosis: a case report and review of the literature.* BMC Psychiatry, 2010. **10**: p. 19.

132. Wilson, J.E., et al., *Delirium.* Nature Reviews Disease Primers, 2020. **6**(1): p. 90.

133. Seritan, A.L., *Advances in the Diagnosis and Management of Psychotic Symptoms in Neurodegenerative Diseases: A Narrative Review.* J Geriatr Psychiatry Neurol, 2023. **36**(6): p. 435-460.

134. Tachibana, M., et al., *Prevalence, clinical features, and risk factors of delusions in patients with delirium.* International Journal of Geriatric Psychiatry, 2022. **37**(6).

135. Webster, R. and S. Holroyd, *Prevalence of Psychotic Symptoms in Delirium.* Psychosomatics, 2000. **41**(6): p. 519-522.

136. Naarding, P., H. Kremer, and F. Zitman, *Huntington's disease: a review of the literature on prevalence and treatment of neuropsychiatric phenomena.* European Psychiatry, 2001. **16**(8): p. 439-445.

137. Rocha, N.P., et al., *The clinical picture of psychosis in manifest huntington's disease: a comprehensive analysis of the Enroll-HD database.* Frontiers in neurology, 2018. **9**: p. 930.

138. Connors, M.H., A. Teixeira-Pinto, and C.T. Loy, *Psychosis and longitudinal outcomes in Huntington disease: the COHORT Study.* Journal of Neurology, Neurosurgery & Psychiatry, 2020. **91**(1): p. 15-20.

139. Tsuang, D., et al., *Familial aggregation of psychotic symptoms in Huntington’s disease.* American Journal of Psychiatry, 2000. **157**(12): p. 1955-1959.

140. Van Duijn, E., E. Kingma, and R. Van Der Mast, *Psychopathology in verified Huntington’s disease gene carriers.* The Journal of neuropsychiatry and clinical neurosciences, 2007. **19**(4): p. 441-448.

141. Ribaï, P., et al., *Psychiatric and Cognitive Difficulties as Indicators of Juvenile Huntington Disease Onset in 29 Patients.* Archives of Neurology, 2007. **64**(6): p. 813-819.

142. Chuo, Y.-P., et al., *Juvenile Huntington's disease presenting as difficult-to-treat seizure and the first episode of psychosis.* General Hospital Psychiatry, 2012. **34**(4): p. 436.e9-436.e11.

143. Sosa-Ortiz, A.L., I. Acosta-Castillo, and M.J. Prince, *Epidemiology of dementias and Alzheimer’s disease.* Archives of medical research, 2012. **43**(8): p. 600-608.

144. Scarmeas, N., et al., *Delusions and hallucinations are associated with worse outcome in Alzheimer disease.* Arch Neurol, 2005. **62**(10): p. 1601-8.

145. Ropacki, S.A. and D.V. Jeste, *Epidemiology of and risk factors for psychosis of Alzheimer’s disease: a review of 55 studies published from 1990 to 2003.* American Journal of Psychiatry, 2005. **162**(11): p. 2022-2030.

146. Kumfor, F., et al., *Examining the presence and nature of delusions in Alzheimer's disease and frontotemporal dementia syndromes.* International Journal of Geriatric Psychiatry, 2022. **37**(3).

147. Devanand, D.P., et al., *Overview of late-onset psychoses.* Int Psychogeriatr, 2024. **36**(1): p. 28-42.

148. Devanand, D.P., et al., *Associations Between Neuropsychiatric Symptoms and Neuropathological Diagnoses of Alzheimer Disease and Related Dementias.* JAMA Psychiatry, 2022. **79**(4): p. 359-367.

149. Pringsheim, T., et al., *The prevalence of Parkinson's disease: a systematic review and meta‐analysis.* Movement disorders, 2014. **29**(13): p. 1583-1590.

150. ffytche, D.H., et al., *The psychosis spectrum in Parkinson disease.* Nature Reviews Neurology, 2017. **13**(2): p. 81-95.

151. Chendo, I., et al., *Frequency and Characteristics of Psychosis in Parkinson's Disease: A Systematic Review and Meta-Analysis.* J Parkinsons Dis, 2022. **12**(1): p. 85-94.

152. Kyle, K. and J.M. Bronstein, *Treatment of psychosis in Parkinson's disease and dementia with Lewy Bodies: A review.* Parkinsonism & Related Disorders, 2020. **75**: p. 55-62.

153. Walker, Z., et al., *Lewy body dementias.* The Lancet, 2015. **386**(10004): p. 1683-1697.

154. Nagahama, Y., et al., *Classification of Psychotic Symptoms in Dementia With Lewy Bodies.* The American Journal of Geriatric Psychiatry, 2007. **15**(11): p. 961-967.

155. O'Brien, J.T. and A. Thomas, *Vascular dementia.* The Lancet, 2015. **386**(10004): p. 1698-1706.

156. Ballard, C., et al., *Anxiety, depression and psychosis in vascular dementia: prevalence and associations.* Journal of affective disorders, 2000. **59**(2): p. 97-106.

157. Onyike, C.U. and J. Diehl-Schmid, *The epidemiology of frontotemporal dementia.* International Review of Psychiatry, 2013. **25**(2): p. 130-137.

158. Xu, L., et al., *Global variation in prevalence and incidence of amyotrophic lateral sclerosis: a systematic review and meta-analysis.* Journal of Neurology, 2020. **267**(4): p. 944-953.

159. Lattante, S., et al., *Defining the genetic connection linking amyotrophic lateral sclerosis (ALS) with frontotemporal dementia (FTD).* Trends in Genetics, 2015. **31**(5): p. 263-273.

160. Devenney, E.M., et al., *Neural mechanisms of psychosis vulnerability and perceptual abnormalities in the ALS‐FTD spectrum.* Annals of Clinical and Translational Neurology, 2021. **8**(8): p. 1576-1591.

161. Chatterjee, A., et al., *A Systematic Review of the Genetics and Pathology of Psychosis in Frontotemporal Dementia.* Canadian Journal of Neurological Sciences, 2023: p. 1-28.

162. Wilcox, A., et al., *Frequency and neural correlates related to psychosis in motor neurone disease.* medRxiv, 2021: p. 2021.03. 31.21253701.

163. Shinagawa, S., et al., *Psychosis in frontotemporal dementia.* Journal of Alzheimer's disease, 2014. **42**(2): p. 485-499.

164. Velakoulis, D., et al., *Frontotemporal dementia presenting as schizophrenia-like psychosis in young people: clinicopathological series and review of cases.* The British Journal of Psychiatry, 2009. **194**(4): p. 298-305.

165. Turner, M.R., et al., *Psychiatric disorders prior to amyotrophic lateral sclerosis.* Annals of neurology, 2016. **80**(6): p. 935-938.

166. Seelen, M., et al., *Prior medical conditions and the risk of amyotrophic lateral sclerosis.* Journal of neurology, 2014. **261**: p. 1949-1956.

167. Taylor, P.N., et al., *Global epidemiology of hyperthyroidism and hypothyroidism.* Nature Reviews Endocrinology, 2018. **14**(5): p. 301-316.

168. Urias-Uribe, L., et al., *Psychosis crisis associated with thyrotoxicosis due to Graves’ disease.* Case reports in psychiatry, 2017. **2017**(1): p. 6803682.

169. Dahale, A.B., et al., *Postpartum psychosis in a woman with Graves' disease: a case report.* General Hospital Psychiatry, 2014. **36**(6): p. 761. e7-761. e8.

170. Brownlie, B.E., et al., *Psychoses associated with thyrotoxicosis - 'thyrotoxic psychosis.' A report of 18 cases, with statistical analysis of incidence.* Eur J Endocrinol, 2000. **142**(5): p. 438-44.

171. Feldman, A.Z., R.T. Shrestha, and J.V. Hennessey, *Neuropsychiatric manifestations of thyroid disease.* Endocrinol Metab Clin North Am, 2013. **42**(3): p. 453-76.

172. Hall, R.C., *Psychiatric effects of thyroid hormone disturbance.* Psychosomatics, 1983. **24**(1): p. 7-11, 15-8.

173. Heinrich, T.W. and G. Grahm, *Hypothyroidism Presenting as Psychosis: Myxedema Madness Revisited.* Prim Care Companion J Clin Psychiatry, 2003. **5**(6): p. 260-266.

174. Haider, A.S., et al., *Autoimmune Schizophrenia? Psychiatric Manifestations of Hashimoto's Encephalitis.* Cureus, 2016. **8**(7): p. e672.

175. Bocchetta, A., et al., *Affective psychosis, Hashimoto's thyroiditis, and brain perfusion abnormalities: case report.* Clinical Practice and Epidemiology in Mental Health, 2007. **3**(1): p. 31.

176. Bharadwaj, B., A. Sugaparaneetharan, and R.P. Rajkumar, *Graves' disease presenting with catatonia: a probable case of encephalopathy associated with autoimmune thyroid disease.* Acta Neuropsychiatrica, 2012. **24**(6): p. 374-379.

177. Menon, V., K. Subramanian, and J.S. Thamizh, *Psychiatric Presentations Heralding Hashimoto's Encephalopathy: A Systematic Review and Analysis of Cases Reported in Literature.* J Neurosci Rural Pract, 2017. **8**(2): p. 261-267.

178. Laurent, C., et al., *Steroid-responsive encephalopathy associated with autoimmune thyroiditis (SREAT): Characteristics, treatment and outcome in 251 cases from the literature.* Autoimmunity Reviews, 2016. **15**(12): p. 1129-1133.

179. Clarke, B.L., et al., *Epidemiology and diagnosis of hypoparathyroidism.* The Journal of Clinical Endocrinology & Metabolism, 2016. **101**(6): p. 2284-2299.

180. GREENE, J.A. and L. Swanson, *Psychosis in hypoparathyroidism; with a report of five cases.* Annals of Internal Medicine, 1941. **14**(7): p. 1233-1236.

181. Nehu Parimi, J.T., et al., *A case of psychosis due to acute hypocalcemia from hypoparathyroidism.* Journal of the Endocrine Society, 2021. **5**(Supplement_1): p. A172-A173.

182. Ang, A.W.-K., S.M. Ko, and C.H. Tan, *Calcium, magnesium, and psychotic symptoms in a girl with idiopathic hypoparathyroidism.* Psychosomatic medicine, 1995. **57**(3): p. 299-302.

183. Minisola, S., et al., *Epidemiology, pathophysiology, and genetics of primary hyperparathyroidism.* Journal of Bone and Mineral Research, 2020. **37**(11): p. 2315-2329.

184. Alarcón, R.D. and J.A. Franceschini, *Hyperparathyroidism and paranoid psychosis: case report and review of the literature.* The British Journal of Psychiatry, 1984. **145**(5): p. 477-486.

185. Park, S. and R. Hieber, *Acute psychosis secondary to suspected hyperparathyroidism: a case report and literature review.* Mental Health Clinician, 2016. **6**(6): p. 304-307.

186. Murphy, R.J., S. Paul, and R. Primelo, *Parathyroid Paranoia: Unveiling Psychosis in Hyperparathyroidism.* Case Reports in Psychiatry, 2024. **2024**(1): p. 8126125.

187. Otsuki, K., et al., *Psychosis in a primary hyperparathyroidism patient with mild hypercalcemia: A case report.* Medicine, 2021. **100**(12): p. e25248.

188. Hakami, O.A., S. Ahmed, and N. Karavitaki, *Epidemiology and mortality of Cushing’s syndrome.* Best practice & research Clinical endocrinology & metabolism, 2021. **35**(1): p. 101521.

189. Mohamed, A.S.M., et al., *Cushing’s disease presenting with psychosis.* Practical Neurology, 2021. **21**(4): p. 351-353.

190. Fujii, Y., et al., *Cushing's syndrome and psychosis: a case report and literature review.* The primary care companion for CNS disorders, 2018. **20**(5): p. 26189.

191. Lin, T.Y., J. Hanna, and W.W. Ishak, *Psychiatric Symptoms in Cushing's Syndrome: A Systematic Review.* Innov Clin Neurosci, 2020. **17**(1-3): p. 30-35.

192. Kelly, W., *Psychiatric aspects of Cushing's syndrome.* QJM: An International Journal of Medicine, 1996. **89**(7): p. 543-552.

193. Husebye, E.S., et al., *Adrenal insufficiency.* The Lancet, 2021. **397**(10274): p. 613-629.

194. Farah, J.d.L., et al., *Severe psychotic disorder as the main manifestation of adrenal insufficiency.* Case Reports in Psychiatry, 2015. **2015**(1): p. 512430.

195. Sanat, Z.M. and M.R. Mohajeri-Tehrani, *Psychotic disorder as the first manifestation of Addison disease: a case report.* International Journal of Endocrinology and Metabolism, 2022. **20**(1).

196. Spiegel, D.R., et al., *A case of psychosis in a patient with secondary adrenal insufficiency: a possible etiological role of a hypocortisolemic-induced increase in proinflammatory cytokines.* Innovations in Clinical Neuroscience, 2017. **14**(9-10): p. 4.

197. McFarland, H.R., *Addison's disease and related psychoses.* Comprehensive Psychiatry, 1963. **4**(2): p. 90-95.

198. Al Subhi, A.R., V. Boyle, and M.S. Elston, *Systematic review: incidence of pheochromocytoma and paraganglioma over 70 years.* Journal of the Endocrine Society, 2022. **6**(9): p. bvac105.

199. Brown, J.S., *Cases of remission of psychosis following resection of pheochromocytoma or paraganglioma.* Schizophrenia Research, 2016. **176**(2): p. 304-306.

200. Mantry, D., et al., *The prevalence and incidence of mental ill‐health in adults with Down syndrome.* Journal of Intellectual Disability Research, 2008. **52**(2): p. 141-155.

201. Dykens, E.M., et al., *Psychiatric disorders in adolescents and young adults with Down syndrome and other intellectual disabilities.* Journal of neurodevelopmental disorders, 2015. **7**: p. 1-8.

202. Ghaziuddin, N., A. Nassiri, and J.H. Miles, *Catatonia in Down syndrome; a treatable cause of regression.* Neuropsychiatric Disease and Treatment, 2015: p. 941-949.

203. Tsiouris, J.A. and W.T. Brown, *Neuropsychiatric Symptoms of Fragile X Syndrome.* CNS Drugs, 2004. **18**(11): p. 687-703.

204. iIndah Winarni, T., et al., *Psychosis and catatonia in fragile X: case report and literature review.* Intractable & Rare Diseases Research, 2015. **4**(3): p. 139-146.

205. Das, P., C.F. Johnston, and S. Hossain, *Schizophrenia in a patient with full mutation of Fragile X gene and intellectual disability: a ‘STEP’towards better understanding.* Psychiatric genetics, 2020. **30**(3): p. 83-86.

206. Groth, K.A., et al., *Klinefelter Syndrome—A Clinical Update.* The Journal of Clinical Endocrinology & Metabolism, 2013. **98**(1): p. 20-30.

207. Cederlöf, M., et al., *Klinefelter syndrome and risk of psychosis, autism and ADHD.* Journal of Psychiatric Research, 2014. **48**(1): p. 128-130.

208. Björlin Avdic, H., et al., *Neurodevelopmental and psychiatric disorders in females with Turner syndrome: a population-based study.* Journal of neurodevelopmental disorders, 2021. **13**: p. 1-9.

209. Prior, T.I., P.S. Chue, and P. Tibbo, *Investigation of Turner syndrome in schizophrenia.* American Journal of Medical Genetics, 2000. **96**(3): p. 373-378.

210. Roser, P. and W. Kawohl, *Turner syndrome and schizophrenia: a further hint for the role of the X-chromosome in the pathogenesis of schizophrenic disorders.* The World Journal of Biological Psychiatry, 2010. **11**(2-2): p. 239-242.

211. McDonald, C., P. Kenna, and T. Larkin, *Retinitis pigmentosa and schizophrenia.* European Psychiatry, 1998. **13**(8): p. 423-426.

212. Waldeck, T., B. Wyszynski, and A. Medalia, *The relationship between Usher's syndrome and psychosis with Capgras syndrome.* Psychiatry, 2001. **64**(3): p. 248-55.

213. Selvakumar, N., et al., *Diagnostic and therapeutic challenges due to psychosis and catatonia in non-syndromic retinitis pigmentosa: A case report.* Indian Journal of Psychological Medicine, 2020. **43**(5): p. 458-459.

214. Salem, H., R. Elkhatib, and T. Pigott, *S246. Psychosis in a Patient With Retinitis Pigmentosa: Beware of D4 Receptor Blockade!* Biological Psychiatry, 2018. **83**(9): p. S443-S444.

215. Shprintzen, R.J., *Velo‐cardio‐facial syndrome: 30 years of study.* Developmental disabilities research reviews, 2008. **14**(1): p. 3-10.

216. Murphy, K.C., L.A. Jones, and M.J. Owen, *High rates of schizophrenia in adults with velo-cardio-facial syndrome.* Archives of general psychiatry, 1999. **56**(10): p. 940-945.

217. Tanham, M., et al., *The effectiveness and tolerability of pharmacotherapy for psychosis in 22q11. 2 Deletion Syndrome: A systematic review.* Australian & New Zealand Journal of Psychiatry, 2024. **58**(5): p. 393-403.

218. Bassett, A.S. and E.W. Chow, *Schizophrenia and 22q11. 2 deletion syndrome.* Current psychiatry reports, 2008. **10**(2): p. 148-157.

219. Gothelf, D., et al., *Clinical characteristics of schizophrenia associated with velo-cardio-facial syndrome.* Schizophrenia research, 1999. **35**(2): p. 105-112.

220. Tanham, M., et al., *The effectiveness and tolerability of pharmacotherapy for psychosis in 22q11.2 Deletion Syndrome: A systematic review.* Aust N Z J Psychiatry, 2024. **58**(5): p. 393-403.

221. Veltman, M.W.M., E.E. Craig, and P.F. Bolton, *Autism spectrum disorders in Prader–Willi and Angelman syndromes: a systematic review.* Psychiatric Genetics, 2005. **15**(4).

222. Aman, L.C.S., et al., *Psychotic illness in people with Prader–Willi syndrome: a systematic review of clinical presentation, course and phenomenology.* Orphanet Journal of Rare Diseases, 2024. **19**(1): p. 69.

223. Aodh, A.M. and A.A. Al-Marshedi, *Williams-Beuren's Syndrome: A Case Report in Prince Sultan Military City, Riyadh, Saudi Arabia 2022.* World Journal of Environmental Biosciences, 2023. **12**(1-2023): p. 20-23.

224. Salgado, H. and L. Martins-Correia, *Williams syndrome and psychosis: a case report.* Journal of Medical Case Reports, 2014. **8**: p. 1-3.

225. Valdes, F., et al., *Brief Report: Major Depressive Disorder with Psychotic Features in Williams Syndrome: A Case Series.* Journal of Autism and Developmental Disorders, 2018. **48**(3): p. 947-952.

226. Rees, E., et al., *Analysis of copy number variations at 15 schizophrenia-associated loci.* The British Journal of Psychiatry, 2014. **204**(2): p. 108-114.

227. Büki, G., K. Hadzsiev, and J. Bene, *Copy Number Variations in Neuropsychiatric Disorders.* International Journal of Molecular Sciences, 2023. **24**(18): p. 13671.

228. Endres, D., et al., *Schizophrenia and Hereditary Polyneuropathy: PMP22 Deletion as a Common Pathophysiological Link?* Frontiers in Psychiatry, 2019. **10**.

229. Grayton, H.M., et al., *Copy number variations in neurodevelopmental disorders.* Progress in Neurobiology, 2012. **99**(1): p. 81-91.

230. Shaikh, T.H., *Copy Number Variation Disorders.* Current Genetic Medicine Reports, 2017. **5**(4): p. 183-190.

231. Filteau, M.-J., et al., *Corpus callosum agenesis and psychosis in Andermann syndrome.* Archives of Neurology, 1991. **48**(12): p. 1275-1280.

232. Fichera, M., et al., *Comorbidities in Friedreich ataxia: incidence and manifestations from early to advanced disease stages.* Neurological Sciences, 2022. **43**(12): p. 6831-6838.

233. Ganos, C., et al., *Psychosis complicating Friedreich ataxia.* Movement Disorders Clinical Practice, 2014. **2**(1): p. 84.

234. Carbone, M.G. and F. Della Rocca, *Neuropsychiatric manifestations of fahr's disease, diagnostic and therapeutic challenge: A case report and a literature review.* Clinical Neuropsychiatry, 2022. **19**(2): p. 121.

235. König, P., *Psychopathological alterations in cases of symmetrical basal ganglia sclerosis.* Biol Psychiatry, 1989. **25**(4): p. 459-68.

236. Saleem, S., et al., *Fahr’s syndrome: literature review of current evidence.* Orphanet journal of rare diseases, 2013. **8**: p. 1-9.

237. Curatolo, P., R. Moavero, and P.J. de Vries, *Neurological and neuropsychiatric aspects of tuberous sclerosis complex.* The Lancet Neurology, 2015. **14**(7): p. 733-745.

238. Pokharel, S., et al., *Tuberous sclerosis complex-associated neuropsychiatric disorder (TAND) in a low-resource setting - From seizure to psychosis: A case report.* Ann Med Surg (Lond), 2020. **60**: p. 734-736.

239. Sekijima, Y., *Hereditary Transthyretin Amyloidosis*. 1993: University of Washington, Seattle, Seattle (WA).

240. Uitti, R.J., et al., *Familial Oculoleptomeningeal Amyloidosis: Report of a New Family With Unusual Features.* Archives of Neurology, 1988. **45**(10): p. 1118-1122.

241. Fattal, O., et al., *Review of the Literature on Major Mental Disorders in Adult Patients With Mitochondrial Diseases.* Psychosomatics, 2006. **47**(1): p. 1-7.

242. Schaefer, A.M., et al., *The epidemiology of mitochondrial disorders—past, present and future.* Biochimica et Biophysica Acta (BBA)-Bioenergetics, 2004. **1659**(2-3): p. 115-120.

243. Anglin, R.E., et al., *The psychiatric manifestations of mitochondrial disorders: a case and review of the literature.* The Journal of clinical psychiatry, 2012. **73**(4): p. 15930.

244. Avşar, P.A., E. Akçay, and E. Gürkaş, *Psychotic attack during the clinical course of megalencephalic leukoencephalopathy with subcortical cysts: a case report.* European Child & Adolescent Psychiatry, 2024.

245. Sexmero, M.M. and J. Blanco, *A late-onset Ornitin Transcabamylase deficiency case as an organic psychosis.* European Psychiatry, 2022. **65**(S1): p. S792-S793.

246. Choi, H.Y., *Adult Onset Ornithine Transcarbamylase Deficiency: A Rare Cause of Psychosis.* BJPsych Open, 2024. **10**(S1): p. S274-S275.

247. Enns, G.M., et al., *Postpartum “psychosis” in mild argininosuccinate synthetase deficiency.* Obstetrics & Gynecology, 2005. **105**(5 Part 2): p. 1244-1246.

248. Higashimoto, T., et al., *Maple syrup urine disease decompensation misdiagnosed as a psychotic event.* Molecular Genetics and Metabolism Reports, 2022. **32**: p. 100886.

249. Maciel, P., et al., *Neuroferritinopathy: missense mutation in FTL causing early-onset bilateral pallidal involvement.* Neurology, 2005. **65**(4): p. 603-605.

250. Mir, P., et al., *Adult‐onset generalized dystonia due to a mutation in the neuroferritinopathy gene.* Movement Disorders: Official Journal of the Movement Disorder Society, 2005. **20**(2): p. 243-245.

251. Bâtie, C.D.d.l., et al., *Acute psychosis in propionic acidemia: 2 case reports.* Journal of child neurology, 2014. **29**(2): p. 274-279.

252. Roze, E., et al., *Neuropsychiatric Disturbances in Presumed Late-Onset Cobalamin C Disease.* Archives of Neurology, 2003. **60**(10): p. 1457-1462.

253. Staretz-Chacham, O., et al., *Psychiatric and behavioral manifestations of lysosomal storage disorders.* American Journal of Medical Genetics Part B: Neuropsychiatric Genetics, 2010. **153B**(7): p. 1253-1265.

254. MacQueen, G.M., P.I. Rosebush, and M.F. Mazurek, *Neuropsychiatric aspects of the adult variant of Tay-Sachs disease.* The Journal of neuropsychiatry and clinical neurosciences, 1998. **10**(1): p. 10-19.

255. Hyde, T.M., J.C. Ziegler, and D.R. Weinberger, *Psychiatric disturbances in metachromatic leukodystrophy: insights into the neurobiology of psychosis.* Archives of neurology, 1992. **49**(4): p. 401-406.

256. Kumperscak, H.G., et al., *Adult metachromatic leukodystrophy: a new mutation in the schizophrenia-like phenotype with early neurological signs.* Psychiatric Genetics, 2007. **17**(2): p. 85-91.

257. van Rappard, D.F., et al., *Slowly progressive psychiatric symptoms: think metachromatic leukodystrophy.* Journal of the American Academy of Child & Adolescent Psychiatry, 2018. **57**(2): p. 74-76.

258. Josephs, K., M. Van Gerpen, and J. Van Gerpen, *Adult onset Niemann-Pick disease type C presenting with psychosis.* Journal of Neurology, Neurosurgery & Psychiatry, 2003. **74**(4): p. 528-529.

259. Shulman, L.M., N.J. David, and W.J. Weiner, *Psychosis as the initial manifestation of adult-onset Niemann-Pick disease type C.* Neurology, 1995. **45**(9): p. 1739-1743.

260. Campo, J.V., et al., *Psychosis as a presentation of physical disease in adolescence: a case of Niemann–Pick disease, type C.* Developmental Medicine & Child Neurology, 1998. **40**(2): p. 126-129.

261. Bonnot, O., et al., *Systematic review of psychiatric signs in Niemann-Pick disease type C.* The World Journal of Biological Psychiatry, 2019.

262. Neil, J.F., R.H. Glew, and S.P. Peters, *Familial psychosis and diverse neurologic abnormalities in adult-onset Gaucher's disease.* Archives of Neurology, 1979. **36**(2): p. 95-99.

263. Akram, S., A. Maqsood, and F. Akram, *Psychosis in Gaucher's Disease.* Psychiatric Annals, 2020. **50**(7): p. 317-320.

264. Tullo, M.G., et al., *The spectrum of neurological and sensory abnormalities in Gaucher disease patients: a multidisciplinary study (SENOPRO).* International Journal of Molecular Sciences, 2023. **24**(10): p. 8844.

265. Imbalzano, G., et al., *Neurological symptoms in adults with Gaucher disease: a systematic review.* Journal of Neurology, 2024: p. 1-11.

266. Seidl, U., et al., *Unusual course of α-mannosidosis with symptoms of paranoid-hallucinatory psychosis.* Der Nervenarzt, 2005. **76**: p. 335-338.

267. Gutschalk, A., et al., *Adult α-mannosidosis: Clinical progression in the absence of demyelination.* Neurology, 2004. **63**(9): p. 1744-1746.

268. Bäckman, M.L., E.T. Aronen, and P.R. Santavuori, *New antidepressive and antipsychotic drugs in juvenile neuronal ceroid lipofuscinoses—a pilot study.* European Journal of Paediatric Neurology, 2001. **5**: p. 163-166.

269. Levinson, A.J. and M.F. Mazurek, *Late-onset adrenoleukodystrophy associated with long-standing psychiatric symptoms.* J Clin Psychiatry, 1999. **60**: p. 460-468.

270. Makkar, H., et al., *Adult-Cerebral X-linked Adrenoleukodystrophy: A Mirage of Psychosis, Mania, and Substance Use.* Psychiatric Annals, 2020. **50**(9): p. 417-420.

271. Fraidakis, M.J., *Psychiatric manifestations in cerebrotendinous xanthomatosis.* Translational Psychiatry, 2013. **3**(9): p. e302-e302.

272. Berginer, V.M., et al., *Psychiatric disorders in patients with cerebrotendinous xanthomatosis.* Am J Psychiatry, 1988. **145**(3): p. 354-7.

273. Ellencweig, N., N. Schoenfeld, and Z. Zemishlany, *Acute intermittent porphyria: psychosis as the only clinical manifestation.* Israel Journal of Psychiatry, 2006. **43**(1): p. 52.

274. Duque-Serrano, L., et al., *Psychiatric aspects of acute porphyria: a comprehensive review.* Current psychiatry reports, 2018. **20**: p. 1-7.

275. Kumar, B., *Acute intermittent porphyria presenting solely with psychosis: a case report and discussion.* Psychosomatics, 2012. **53**(5): p. 494-498.

276. Zimbrean, P.C. and M.L. Schilsky, *Psychiatric aspects of Wilson disease: a review.* General hospital psychiatry, 2014. **36**(1): p. 53-62.

277. Litwin, T., et al., *Psychiatric manifestations in Wilson’s disease: possibilities and difficulties for treatment.* Therapeutic advances in psychopharmacology, 2018. **8**(7): p. 199-211.

278. Cox, D.W., *A screening test for Wilson's disease and its application to psychiatric patients.* Can Med Assoc J, 1967. **96**(2): p. 83-6.

279. Brumm, V.L., D. Bilder, and S.E. Waisbren, *Psychiatric symptoms and disorders in phenylketonuria.* Molecular Genetics and Metabolism, 2010. **99**: p. S59-S63.

280. Bilder, D.A., et al., *Psychiatric symptoms in adults with phenylketonuria.* Molecular Genetics and Metabolism, 2013. **108**(3): p. 155-160.

281. Bilder, D.A., et al., *Neuropsychiatric comorbidities in adults with phenylketonuria: A retrospective cohort study.* Molecular Genetics and Metabolism, 2017. **121**(1): p. 1-8.

282. Hector, M. and J.R. Burton, *What are the psychiatric manifestations of vitamin B12 deficiency?* J Am Geriatr Soc, 1988. **36**(12): p. 1105-12.

283. Green, R., et al., *Vitamin B12 deficiency.* Nature reviews Disease primers, 2017. **3**(1): p. 1-20.

284. Parikh, D. and S. Panse, *Pellagra induced psychosis: A rare presentation.* International Journal of Research in Medical Sciences, 2019. **7**(4): p. 1364-1366.

285. Prakash, R., et al., *Rapid resolution of delusional parasitosis in pellagra with niacin augmentation therapy.* General hospital psychiatry, 2008. **30**(6): p. 581-584.

286. Palm, A., et al., *Incidence and mortality of alcohol‐related dementia and Wernicke‐Korsakoff syndrome: A nationwide register study.* International Journal of Geriatric Psychiatry, 2022. **37**(8).

287. Arts, N.J., S.J. Walvoort, and R.P. Kessels, *Korsakoff’s syndrome: a critical review.* Neuropsychiatric disease and treatment, 2017: p. 2875-2890.

288. THOMSON, A.D. and E.J. MARSHALL, *THE NATURAL HISTORY AND PATHOPHYSIOLOGY OF WERNICKE'S ENCEPHALOPATHY AND KORSAKOFF'S PSYCHOSIS.* Alcohol and Alcoholism, 2005. **41**(2): p. 151-158.

289. Harper, C., M. Giles, and R. Finlay-Jones, *Clinical signs in the Wernicke-Korsakoff complex: a retrospective analysis of 131 cases diagnosed at necropsy.* Journal of Neurology, Neurosurgery & Psychiatry, 1986. **49**(4): p. 341-345.

290. Gerridzen, I.J., et al., *Prevalence and severity of behavioural symptoms in patients with Korsakoff syndrome and other alcohol‐related cognitive disorders: A systematic review.* International journal of geriatric psychiatry, 2017. **32**(3): p. 256-273.

291. Kales, S.N., et al., *Paranoid Psychosis after Exposure to Cyanide.* Archives of Environmental Health: An International Journal, 1997. **52**(3): p. 245-246.

292. Rutchik, J. and M.H. Ratner, *Is it Possible for Late-Onset Schizophrenia to Masquerade as Manganese Psychosis?* Journal of Occupational and Environmental Medicine, 2018. **60**(4).

293. Mason, L.H., M.J. Mathews, and D.Y. Han, *Neuropsychiatric symptom assessments in toxic exposure.* Psychiatr Clin North Am, 2013. **36**(2): p. 201-8.

294. WU, H.E., et al., *An Unusual Case of Acute Psychosis With Obsessive-Compulsive Features Following Arsenic Poisoning.* Journal of Psychiatric Practice®, 2017. **23**(5): p. 382-385.

295. Huang, X., et al., *Mercury poisoning: a case of a complex neuropsychiatric illness.* American Journal of Psychiatry, 2014. **171**(12): p. 1253-1256.

296. Fagala, G.E. and C.L. Wigg, *Psychiatric Manifestations of Mercury Poisoning.* Journal of the American Academy of Child & Adolescent Psychiatry, 1992. **31**(2): p. 306-311.

297. Goldbloom, D. and G. Chouinard, *Schizophreniform psychosis associated with chronic industrial toluene exposure: case report.* The journal of clinical psychiatry, 1985. **46**(8): p. 350-351.

298. Stein, Y., et al., *Exposure and susceptibility: Schizophrenia in a young man following prolonged high exposures to organic solvents.* NeuroToxicology, 2010. **31**(5): p. 603-607.

299. Kohlmeier, R.E., *Chronic Lead Poisoning: Induced Psychosis in an Adult?* The American Journal of Forensic Medicine and Pathology, 2002. **23**(1): p. 101.

300. Vorvolakos, T., S. Arseniou, and M. Samakouri, *There is no safe threshold for lead exposure: Α literature review.* Psychiatriki, 2016. **27**(3): p. 204-214.

301. Magoub, N. and M. Tahir, *Is it medication-induced psychosis or prodromal psychosis unmasked by medication?* The Journal of Neuropsychiatry and Clinical Neurosciences, 2011. **23**(3): p. E13-E14.

302. Reinhardt, M.M. and C.I. Cohen, *Late-Life Psychosis: Diagnosis and Treatment.* Current Psychiatry Reports, 2015. **17**(2): p. 1.

303. Wood, K.A., et al., *Drug-Induced Psychosis and Depression in the Elderly.* Psychiatric Clinics of North America, 1988. **11**(1): p. 167-193.

304. Barateau, L., et al., *Narcolepsy.* Journal of Sleep Research, 2022. **31**(4): p. e13631.

305. Hanin, C., et al., *Narcolepsy and psychosis: A systematic review.* Acta Psychiatrica Scandinavica, 2021. **144**(1): p. 28-41.

306. Fortuyn, H.A.D., et al., *Psychotic symptoms in narcolepsy: phenomenology and a comparison with schizophrenia.* General Hospital Psychiatry, 2009. **31**(2): p. 146-154.

307. Arnulf, I., et al., *Kleine–Levin syndrome: a systematic review of 186 cases in the literature.* Brain, 2005. **128**(12): p. 2763-2776.

308. Luks, A.M., E.R. Swenson, and P. Bärtsch, *Acute high-altitude sickness.* European Respiratory Review, 2017. **26**(143).

309. Hüfner, K., et al., *Isolated high altitude psychosis, delirium at high altitude, and high altitude cerebral edema: are these diagnoses valid?* Frontiers in Psychiatry, 2023. **14**: p. 1221047.

310. Howard, R., et al., *Late-onset schizophrenia and very-late-onset schizophrenia-like psychosis: an international consensus. The International Late-Onset Schizophrenia Group.* Am J Psychiatry, 2000. **157**(2): p. 172-8.

311. Kane, J.M., et al., *The expert consensus guideline series. Optimizing pharmacologic treatment of psychotic disorders. Introduction: methods, commentary, and summary.* J Clin Psychiatry, 2003. **64 Suppl 12**: p. 5-19.

312. *International clinical practice guidelines for early psychosis.* Br J Psychiatry Suppl, 2005. **48**: p. s120-4.

313. Keshavan, M.S., M. Roberts, and D. Wittmann, *Guidelines for clinical treatment of early course schizophrenia.* Curr Psychiatry Rep, 2006. **8**(4): p. 329-34.

314. Leucht S, A.C., Fleischhacker WW, Kapur S, Stroup S, van Os J, Correll CU *CINP Schizophrenia Guideline* 2011.

315. Leucht, S., et al., *Evidence-based pharmacotherapy of schizophrenia.* Int J Neuropsychopharmacol, 2011. **14**(2): p. 269-84.

316. Stahl, S.M., et al., *“Meta-guidelines” for the management of patients with schizophrenia.* CNS Spectr, 2013. **18**(3): p. 150-162.

317. Ostuzzi, G., et al., *Mapping the evidence on pharmacological interventions for non-affective psychosis in humanitarian non-specialised settings: a UNHCR clinical guidance.* BMC Med, 2017. **15**(1): p. 197.

318. Falkai, P., et al., *World Federation of Societies of Biological Psychiatry (WFSBP) guidelines for biological treatment of schizophrenia, Part 1: acute treatment of schizophrenia.* World J Biol Psychiatry, 2005. **6**(3): p. 132-91.

319. Falkai, P., et al., *World Federation of Societies of Biological Psychiatry (WFSBP) guidelines for biological treatment of schizophrenia, part 2: long-term treatment of schizophrenia.* World J Biol Psychiatry, 2006. **7**(1): p. 5-40.

320. Hasan, A., et al., *World Federation of Societies of Biological Psychiatry (WFSBP) Guidelines for Biological Treatment of Schizophrenia, part 1: update 2012 on the acute treatment of schizophrenia and the management of treatment resistance.* World J Biol Psychiatry, 2012. **13**(5): p. 318-78.

321. Hasan, A., et al., *World Federation of Societies of Biological Psychiatry (WFSBP) guidelines for biological treatment of schizophrenia, part 2: update 2012 on the long-term treatment of schizophrenia and management of antipsychotic-induced side effects.* World J Biol Psychiatry, 2013. **14**(1): p. 2-44.

322. Hasan, A., et al., *World Federation of Societies of Biological Psychiatry (WFSBP) Guidelines for Biological Treatment of Schizophrenia. Part 3: Update 2015 Management of special circumstances: Depression, Suicidality, substance use disorders and pregnancy and lactation.* World J Biol Psychiatry, 2015. **16**(3): p. 142-70.

323. Hasan, A., et al., *World Federation of Societies of Biological Psychiatry (WFSBP) guidelines for biological treatment of schizophrenia - a short version for primary care.* Int J Psychiatry Clin Pract, 2017. **21**(2): p. 82-90.

324. Hasan, A., et al., *[National and international schizophrenia guidelines. Update 2013 regarding recommendations about antipsychotic pharmacotherapy].* Nervenarzt, 2013. **84**(11): p. 1359-60, 1362-4, 1366-8.

325. Kane, J.M., et al., *Clinical Guidance on the Identification and Management of Treatment-Resistant Schizophrenia.* J Clin Psychiatry, 2019. **80**(2).

326. Skikic, M. and J.A. Arriola, *First Episode Psychosis Medical Workup: Evidence-Informed Recommendations and Introduction to a Clinically Guided Approach.* Child Adolesc Psychiatr Clin N Am, 2020. **29**(1): p. 15-28.

327. Howes, O.D., et al., *Treatment-Resistant Schizophrenia: Treatment Response and Resistance in Psychosis (TRRIP) Working Group Consensus Guidelines on Diagnosis and Terminology.* Am J Psychiatry, 2017. **174**(3): p. 216-229.

328. Wagner, E., et al., *Clozapine Combination and Augmentation Strategies in Patients With Schizophrenia -Recommendations From an International Expert Survey Among the Treatment Response and Resistance in Psychosis (TRRIP) Working Group.* Schizophr Bull, 2020. **46**(6): p. 1459-1470.

329. Wagner, E., et al., *Clozapine Optimization: A Delphi Consensus Guideline From the Treatment Response and Resistance in Psychosis Working Group.* Schizophr Bull, 2023. **49**(4): p. 962-972.

330. Emsley, R., et al., *The South African Society of Psychiatrists (SASOP) Treatment Guidlelines for Psychiatric Disorders.* 2013, 2013. **19**(3).

331. Disayavanish, C., et al., *Guideline for the pharmacotherapy of treatment-resistant schizophrenia. Royal College of Psychiatrists of Thailand.* J Med Assoc Thai, 2000. **83**(6): p. 579-89.

332. Verma, S., et al., *Ministry of Health clinical practice guidelines: schizophrenia.* Singapore Med J, 2011. **52**(7): p. 521-5; quiz 526.

333. Lo, T.L., et al., *Recommendations for the optimal care of patients with recent-onset psychosis in the Asia-Pacific region.* Asia Pac Psychiatry, 2016. **8**(2): p. 154-71.

334. Grover, S., et al., *Clinical Practice Guidelines for Management of Schizophrenia.* Indian J Psychiatry, 2017. **59**(Suppl 1): p. S19-s33.

335. Grover, S. and A. Avasthi, *Clinical Practice Guidelines for the Management of Schizophrenia in Children and Adolescents.* Indian J Psychiatry, 2019. **61**(Suppl 2): p. 277-293.

336. Lee, J.S., et al., *Korean Medication Algorithm for Schizophrenia 2019, Second Revision: Treatment of Psychotic Symptoms.* Clin Psychopharmacol Neurosci, 2020. **18**(3): p. 386-394.

337. Yun, J.-Y., et al., *Korean Treatment Guideline on Pharmacotherapy of Co-existing Symptoms and Antipsychotics-related Side Effects in Patients with Schizophrenia.* kjsr, 2019. **22**(2): p. 21-33.

338. Sakurai, H., et al., *Pharmacological Treatment of Schizophrenia: Japanese Expert Consensus.* Pharmacopsychiatry, 2021. **54**(2): p. 60-67.

339. Neuropsychopharmacology, J.S.o., *Japanese Society of Neuropsychopharmacology: “Guideline for Pharmacological Therapy of Schizophrenia”.* Neuropsychopharmacology Reports, 2021. **41**(3): p. 266-324.

340. Ministry of Health, M. *Management of Schizophrenia (Second Edition)*. 5/9/24]; Available from: <https://www.moh.gov.my/moh/resources/Main%20Banner/2021/Okt/Draft_CPG_Management_of_Schizophrenia_(Second_Edition)_for_Reviewers.pdf>.

341. *Guidelines for schizophrenia Prevention and Treatment in China*. 2023 3/9/24]; Available from: <http://guidelines-registry.cn/guid/2386>.

342. Petrakis, M., et al., *Fidelity to clinical guidelines using a care pathway in the treatment of first episode psychosis.* J Eval Clin Pract, 2011. **17**(4): p. 722-8.

343. *Early Psychosis Guidelines Writing Group and EPPIC National Support Program, Australian Clinical Guidelines for Early Psychosis*. 2016 1/4/2024]; 2nd edition update:[

344. Galletly, C., et al., *Royal Australian and New Zealand College of Psychiatrists clinical practice guidelines for the management of schizophrenia and related disorders.* Aust N Z J Psychiatry, 2016. **50**(5): p. 410-72.

345. *Royal Australian and New Zealand College of Psychiatrists clinical practice guidelines for the treatment of schizophrenia and related disorders.* Aust N Z J Psychiatry, 2005. **39**(1-2): p. 1-30.

346. De Masi, S., et al., *The Italian guidelines for early intervention in schizophrenia: development and conclusions.* Early Interv Psychiatry, 2008. **2**(4): p. 291-302.

347. Castelein, S., et al., *[Dutch guideline on Schizophrenia 2012: basic care within the areas of psychosocial interventions and nursing care].* Tijdschr Psychiatr, 2013. **55**(9): p. 707-14.

348. *Landelijke Stuurgroep Multidisciplinaire Richtlijnontwikkeling in de GGZMultidisciplinaire Richtlijn Schizofrenie [Multidisciplinairy Guideline Schizophrenia]*. 2005; Available from: <https://www.gerdierx.nl/wp-content/uploads/2012/02/Multidisciplinaire-richtlijn-Schizofrenie.pdf>.

349. Bobes-García, J., et al., *Delphi consensus on the physical health of patients with schizophrenia: evaluation of the recommendations of the Spanish Societies of Psychiatry and Biological Psychiatry by a panel of experts.* Actas Esp Psiquiatr, 2012. **40**(3): p. 114-28.

350. Petitjean, F., *[Therapeutic guidelines in schizophrenias].* Encephale, 2006. **32**(5 Pt 3): p. S855-7.

351. Llorca, P.M., et al., *Guidelines for the use and management of long-acting injectable antipsychotics in serious mental illness.* BMC Psychiatry, 2013. **13**: p. 340.

352. *Scottish Intercollegiate Guidelines Network (SIGN), Management of schizophrenia*. SIGN publication no. 131 2013 1/4/24]; Available from: <http://www.sign.ac.uk>.

353. *Psychosis and schizophrenia in adults: prevention and management. London: National Institute for Health and Care Excellence (NICE)*. 2014 12/8/24]; Available from: (NICE Clinical Guidelines, No. 178.) Available from: <https://www.ncbi.nlm.nih.gov/books/NBK555203/>.

354. Baandrup, L., et al., *Treatment of adult patients with schizophrenia and complex mental health needs - A national clinical guideline.* Nord J Psychiatry, 2016. **70**(3): p. 231-40.

355. Lass, J., A. Männik, and J.S. Bell, *Pharmacotherapy of first episode psychosis in Estonia: comparison with national and international treatment guidelines.* J Clin Pharm Ther, 2008. **33**(2): p. 165-73.

356. Männik, A., J. Lass, and J.S. Bell, *Esmase skisofreenia farmakoteraapia Eestis: võrdlus kohaliku ja rahvusvaheliste ravijuhistega.* Eesti Arst, 2008.

357. Hadjulis, M., et al., *[Clinical guidelines for the management of schizophrenia: Pharmacological and psychological interventions (III)].* Psychiatriki, 2018. **29**(4): p. 303-315.

358. von Malortie, S., et al., *[New national guidelines for the treatment of schizophrenia in Sweden].* Lakartidningen, 2019. **116**.

359. German Society for Psychiatry and Psychotherapy, P.a.N.D.e., *S3 guideline schizophrenia. Short Version*. March 15, 2019.

360. Barnes, T.R., *Evidence-based guidelines for the pharmacological treatment of schizophrenia: recommendations from the British Association for Psychopharmacology.* J Psychopharmacol, 2011. **25**(5): p. 567-620.

361. Cooper, S.J., et al., *BAP guidelines on the management of weight gain, metabolic disturbances and cardiovascular risk associated with psychosis and antipsychotic drug treatment.* J Psychopharmacol, 2016. **30**(8): p. 717-48.

362. Barnes, T.R., et al., *Evidence-based guidelines for the pharmacological treatment of schizophrenia: Updated recommendations from the British Association for Psychopharmacology.* J Psychopharmacol, 2020. **34**(1): p. 3-78.

363. *Schizophrenia: current care guidelines*. 2020 15/4/2024]; Available from: <https://www.kaypahoito.fi/hoi35050>.

364. Galderisi, S., et al., *Identification and management of cardiometabolic risk in subjects with schizophrenia spectrum disorders: A Delphi expert consensus study.* Eur Psychiatry, 2021. **64**(1): p. e7.

365. Galderisi, S., et al., *EPA guidance on treatment of negative symptoms in schizophrenia.* Eur Psychiatry, 2021. **64**(1): p. e21.

366. Schmidt, S.J., et al., *EPA guidance on the early intervention in clinical high risk states of psychoses.* Eur Psychiatry, 2015. **30**(3): p. 388-404.

367. Janas-Kozik, M., et al., *Polish Psychiatric Association diagnostic and therapeutic management guidelines for patients with early-onset schizophrenia.* Psychiatr Pol, 2022. **56**(4): p. 675-695.

368. Wichniak, A., et al., *Metabolic risk reduction in patients with schizophrenia treated with antipsychotics: recommendations of the Polish Psychiatric Association.* Psychiatr Pol, 2019. **53**(6): p. 1191-1218.

369. Marder, S.R., et al., *Physical health monitoring of patients with schizophrenia.* Am J Psychiatry, 2004. **161**(8): p. 1334-49.

370. Moore, T.A., et al., *The Texas Medication Algorithm Project antipsychotic algorithm for schizophrenia: 2006 update.* J Clin Psychiatry, 2007. **68**(11): p. 1751-62.

371. Lehman, A.F., et al., *Practice guideline for the treatment of patients with schizophrenia, second edition.* Am J Psychiatry, 2004. **161**(2 Suppl): p. 1-56.

372. Kreyenbuhl, J., et al., *The Schizophrenia Patient Outcomes Research Team (PORT): updated treatment recommendations 2009.* Schizophr Bull, 2010. **36**(1): p. 94-103.

373. Young, A.S., et al., *Routine outcomes monitoring to support improving care for schizophrenia: report from the VA Mental Health QUERI.* Community Ment Health J, 2011. **47**(2): p. 123-35.

374. *AACAP official action. Summary of the practice parameters for the assessment and treatment of children and adolescents with schizophrenia. American Academy of Child and Adolescent Psychiatry.* J Am Acad Child Adolesc Psychiatry, 2000. **39**(12): p. 1580-2.

375. McClellan, J. and S. Stock, *Practice parameter for the assessment and treatment of children and adolescents with schizophrenia.* J Am Acad Child Adolesc Psychiatry, 2013. **52**(9): p. 976-90.

376. Abidi, S., et al., *Canadian Guidelines for the Pharmacological Treatment of Schizophrenia Spectrum and Other Psychotic Disorders in Children and Youth.* Can J Psychiatry, 2017. **62**(9): p. 635-647.

377. Addington, D., et al., *Canadian Practice Guidelines for Comprehensive Community Treatment for Schizophrenia and Schizophrenia Spectrum Disorders.* Can J Psychiatry, 2017. **62**(9): p. 662-672.

378. Addington, D., et al., *Canadian Guidelines for the Assessment and Diagnosis of Patients with Schizophrenia Spectrum and Other Psychotic Disorders.* Can J Psychiatry, 2017. **62**(9): p. 594-603.

379. Canadian Psychiatric, A., *CLINICAL PRACTICE GUIDELINES: Treatment of Schizophrenia.* Canadian Journal of Psychiatry, suppl. Clinical Practice Guidelines Treatment of Schizophrenia, 2005. **50**(13): p. 7S-57S.

380. Pringsheim, T., et al., *Physical Health and Drug Safety in Individuals with Schizophrenia.* The Canadian Journal of Psychiatry, 2017. **62**(9): p. 673-683.

381. (AACP), A.A.o.C.P. *Clinical Tips Series, Long Acting Antipsychotic Medications*. 2017 15/4/2024]; Available from: <https://drive.google.com/file/d/1unigjmjFJkqZMbaZ_ftdj8oqog49awZs/view>.

382. Association, A.P., *Practice guideline for the treatment of patients with schizophrenia.* Am J Psychiatry, 2004. **161**(2): p. 1-57.

383. Association, A.P., *The American Psychiatric Association practice guideline for the treatment of patients with schizophrenia*. 2020: American Psychiatric Pub.

384. Dixon, L., D. Perkins, and C. Calmes, *Guideline watch (September 2009): practice guideline for the treatment of patients with schizophrenia.* Arlington: American Psychiatric Association, 2009.

385. Program, F.M.D.T.M. *2019–2020 Florida Best Practice Psychotherapeutic Medication Guidelines for Adults*. 2020 15/4/2024]; Available from: <https://floridabhcenter.org/wp-content/uploads/2021/04/2019-Psychotherapeutic-Medication-Guidelines-for-Adults-with-References_06-04-20.pdf>.

386. Jonokuchi, A.J., et al., *Approach to New-Onset Psychosis in Pediatrics: A Review of Current Practice and an Interdisciplinary Consensus-Driven Clinical Pathway at a Single-Center Institution.* Journal of child neurology, 2023. **38**(3-4): p. 216-222.

387. Markkula, N., R. Alvarado, and A. Minoletti, *Adherence to guidelines and treatment compliance in the Chilean national program for first-episode schizophrenia.* Psychiatr Serv, 2011. **62**(12): p. 1463-9.

388. Gaspar, P.A., et al., *Early psychosis detection program in Chile: a first step for the South American challenge in psychosis research.* Early intervention in psychiatry, 2019. **13**(2): p. 328-334.

389. Escamilla-Orozco, R.I., et al., *Tratamiento de la esquizofrenia en México: recomendaciones de un panel de expertos.* Gac Med Mex, 2021. **157**(Supl 4): p. S1-s12.

**Appendix 5: Table: Included Guideline Findings**

| **Guideline** | **Year** | **Routine** | **As Clinically Indicated** |
| --- | --- | --- | --- |
| Expert consensus optimising pharmacotherapy, Kane et al. | 2003 | Glucose, Lipids, Prolactin, HIV, ECG |  |
| Mount Sinai Conference Consensus | 2004 | HbA1c, Glucose, Lipids, | Prolactin, ECG |
| Guidelines for early course of schizophrenia, Keshavan et al. | 2006 | FBC, E/LFT, Glucose, Lipids, TSH, UDS | EEG, Neuroimaging |
| Spanish Societies of Psychiatry and Biological Psychiatry | 2012 | FBC, E/LFT, Glucose, Lipids, Prolactin, ECG | HBV, HCV, HIV, Syphilis |
| American Academy of Child and Adolescent Psychiatry | 2013 | FBC, E/LFT, Glucose, Lipids, TSH | Copper, Karyotyping, EEG, Neuroimaging, CSF |
| French Association for Biological Psychiatry and Neuropsychopharmacology | 2013 | FBC, E/LFT, Glucose, Lipids, BHCG, ECG | TSH, Prolactin, EEG |
| Scottish Intercollegiate Guidelines Network | 2013 | HbA1c, Glucose, Lipids, | Prolactin, ECG |
| The South African Society of Psychiatrists | 2013 | FBC, E/LFT, HbA1c, Glucose, Lipids, HIV, Syphilis, UDS | ECG, EEG, Neuroimaging |
| "Meta-Guidelines", Stahl et.al. | 2013 | FBC, E/LFT, HbA1c, Glucose, Lipids, TSH, HCV, HIV, Syphilis | Prolactin, Heavy metals, BHCG, ECG, UDS, EEG, Neuroimaging |
| Orygen, Australian Clinical Guidelines for Early Psychosis | 2016 | FBC, E/LFT, Glucose, Lipids, ANA, CRP/ESR, TSH, Vitamins, Prolactin, Copper, HIV, Syphilis, UDS, Neuroimaging (MRI) | HCV, Heavy metals, Karyotyping, ECG, EEG, CSF |
| Royal Australian and New Zealand College of Psychiatrists | 2016 | FBC, E/LFT, Glucose, Lipids, CRP/ESR, Neuronal Ab, HBV, HCV, ECG, UDS, Neuroimaging (MRI) | HIV, Syphilis, EEG |
| World Federation of Societies of Biological Psychiatry | 2017 | FBC, Glucose, Lipids, BHCG, ECG, UDS, EEG, Neuroimaging | CSF |
| Canadian Psychiatric Association | 2017 | HbA1c, Glucose, Lipids | Prolactin, Karyotyping, ECG, Neuroimaging |
| Greek Ministry of Health | 2018 | FBC, E/LFT, HbA1C, Glucose, Lipids, Prolactin | ECG |
| The University of Chile High-risk Intervention Program | 2019 | E/LFT, Glucose, Lipids, TSH | HIV, Syphilis, ECG, UDS, EEG, Neuroimaging |
| German Association for Psychiatry, Psychotherapy and Psychosomatics | 2019 | FBC, E/LFT, HbA1c, Glucose, CRP/ESR, TSH, BHCG, ECG, UDS, Neuroimaging (MRI) | ANA, Vitamins, Copper, Neuronal Abs, HBV, HCV, HIV, Syphilis, EEG, CSF |
| Indian Psychiatric Society | 2019 | FBC, E/LFT, Glucose, Lipids, TSH, EEG, Neuroimaging (MRI) | Vitamins, Prolactin, Copper |
| Polish Psychiatric Association | 2019 | FBC, E/LFT, Glucose, Lipids, Prolactin, ECG |  |
| American Psychiatric Association | 2020 | FBC, E/LFT, HbA1c, Glucose, Lipids, TSH, BHCG | Prolactin, Karyotyping, ECG, UDS, EEG, Neuroimaging |
| British Association of Psychopharmacology | 2020 | E/LFT, HbA1c, Glucose, Lipids, CRP/ESR, TSH, Prolactin, ECG, UDS | ANA, Vitamins, Neuronal Abs, Heavy metals, EEG, Neuroimaging, CSF |
| First Episode Psychosis Medical Workup, Skikic and Arriola | 2020 | FBC, E/LFT, HbA1c, Glucose, Lipids, TSH, Vitamins, Copper, HIV, Syphilis, BHCG, ECG, UDS, EEG | ANA, Neuronal Abs, Heavy metals, Karyotyping, Neuroimaging, CSF |
| Finnish Medical Association and the Finnish Psychiatry Association | 2020 | FBC, E/LFT, HbA1c, Glucose, Lipids, CRP/ESR, TSH, ECG, UDS, Neuroimaging (MRI) | Neuronal Abs, BHCG, EEG |
| Ministry for Health, Malaysia | 2021 | FBC, E/LFT, Glucose, Lipids, Prolactin, ECG |  |
| National Institute for Health and Care Excellence | 2021 | HbA1c, Glucose, Lipids, Prolactin | ECG |
| Columbia University | 2023 | FBC, E/LFT, HbA1c, Lipids, ANA, CRP/ESR, TSH, Vitamins, Prolactin, UDS, Neuroimaging | Glucose, Copper, Neuronal Abs, HCV, HIV, Syphilis, Heavy metals, BHCG, EEG, CSF |
